# Supplementary material for: Effect of multiple risk behaviours in adolescence on educational attainment at age 16 years: a UK birth cohort study
Source: BMJ Open. 2018 Jul 30;8(7):e020182. doi: 10.1136/bmjopen-2017-020182 (PMC6067358; doi:10.1136/bmjopen-2017-020182)
Supplement: Supplementary file 1 [file bmjopen-2017-020182supp001.pdf]

Supplementary material table 1: Assessment for confounding, univariable associations with the exposure and outcome variables

	MRBs	Capped GCSE	Five or more GCSEs A*-C
**Gender ref: male**			
Female	-0.06 (-0.18, 0.06) p=0.343	10.83 (6.85, 14.81) p<0.001	1.32 (1.17, 1.49) p<0.001
**Season of birth ref: autumn**			
Winter	-0.04 (-0.23, 0.15) p=0.700	-4.18 (-10.35, 1.99) p=0.184	0.95 (0.79, 1.16) p=0.637
Spring	0.04 (-0.13, 0.20) p=0.674	-10.37 (-15.69, -5.05) p<0.001	0.78 (0.67, 0.92) p=0.004
Summer	0.26 (-0.13, 0.18) p=0.744	-13.34 (-18.40, -8.28) p<0.001	0.83 (0.71, 0.97) p=0.018
**Maternal education ref: degree**			
A level	0.17 (-0.02, 0.36) p=0.074	-35.19 (-41.13, -29.25) p<0.001	0.18 (0.12, 0.27) p<0.001
O level	0.41 (0.23, 0.59) p<0.001	-57.89 (-63.55, -52.23) p<0.001	0.09 (0.06, 0.14) p<0.001
< O level	0.49 (0.28, 0.69) p<0.001	-90.81 (-96.95, -84.68) p<0.001	0.04 (0.25, 0.06) p<0.001
**Parental socio-economic position ref: professional**			
Managerial and technical	0.43 (0.25, 0.61) p<0.001	-35.76 (-41.46, -30.05) p<0.001	0.29 (0.21, 0.39) p<0.001
Skilled non-man	0.47 (0.28, 0.67) p<0.001	-58.39 (-64.63, -52.15) p<0.001	0.15 (0.11, 0.21) p<0.001
Skilled man, part or unskilled	0.83 (0.60, 1.05) p<0.001	-88.71 (-95.76, -81.64) p<0.001	0.07 (0.05, 0.09) p<0.001

**Housing tenure ref: mortgage/owned**			
Private rent	0 .38 (0.14, 0.61) p=0.002	-20.07 (-27.50, -12.64) p<0.001	0.59 (0.47, 0.74) p<0.001
Subsidised rent	0 .75 (0.53, 0.97) p<0.001	-71.86 (-78.71, -65.01) p<0.001	0.18 (0.15, 0.74) p<0.001
**Income ref: high**			
Mid high	0.05 (-0.14, 0.24) p=0.615	-12.17 (-18.09, -6.26) p<0.001	0.62 (0.47, 0.80) p<0.001
Middle	0.17 (-0.02, 0.36) p=0.084	-32.02 (-37.98, -26.07) p<0.001	0.34 (0.26, 0.43) p<0.001
Mid low	0.35 (0.15, 0.55) p<0.001	-45.62 (-51.78, -39.46) p<0.001	0.23 (0.18, 0.29) p<0.001
Low	0.43 (0.23, 0.64) p<0.001	-74.31 (-80.75, -67.87) p<0.001	0.13 (0.10, 0.16) p<0.001
**FSM ref: never FSM**			
Ever FSM	0.97 (0.74, 1.21) p<0.001	-68.52 (-75.74, -61.30) p<0.001	0.22 (0.18, 0.27) p<0.001
**IQ at age 8**	-.0009 (-.0010, -.0006) p=0.009	2.69 (2.58, 2.79) p<0.001	1.09 (1.09, 1.10) p<0.001
**KS2 educational attainment**	-0.01 (-0.01, -0.01) p<0.001	0.26 (0.259, 0 .278) p<0.001	1.01 (1.01, 1.01) p<0.001
**SEN ref: no SEN**			
School Action	0.76 (-0.18, 0.33) p=0.555	-64.63 (-73.46, -55.80) p<0.001	0.19 (0.15, 0.25) p<0.001
School Action Plus	0.30 (-0.16, 0.77) p=0.201	-82.35 (-97.93, -66.78) p<0.001	0.13 (0.09, 0.21) p<0.001
Statement of SEN	-0.39 (-0.90, 0.12) p=0.134	-133.76 (-152.96, -114.56) p<0.001	0.11 (0.06, 0.19) p<0.001

Supplementary material table 2: Associations between total MRB score and educational outcomes, capped GCSE score and gaining five or more GCSEs at grade A*-C (complete case analysis)

	Complete Case Unadjusted (n=1,617)	Complete Case Adjusted[1] (n=1,617)	Complete Case Adjusted[2] (n=1,617)	Complete Case Adjusted[3] (n=1,617)
**Capped GCSE**	-6.12 (-7.42, -4.83) p<0.001	-6.13 (-7.42, -4.84) p<0.001	-4.82 (-6.00, -3.64) p<0.001	-4.48 (-5.31, -3.64) p<0.001
**5 A*-C**	0.81 (0.76–0.87) p<0.001	0.81 (0.75–0.87) p<0.001	0.84 (0.77-0.90) p<0.001	0.75 (0.68–0.83) p<0.001

[1] Adjusted for sex and season of birth.
[2] Adjusted for sex, season of birth maternal education, parental social class, household income, tenure and FSM.
[3] Adjusted for sex, season of birth maternal education, parental social class, household income, tenure FSM, IQ at age 8 and previous educational attainment at key stage 2.

Supplementary material table 3: Associations between separate MRB and educational outcomes, capped GCSE score and gaining five or more GCSEs at grade A*-C

	Capped GCSE score	Five or more GCSEs A*-C
**Car passenger risk**	-19.90 (-25.13, -14.67)	0.68 (0.58, 0.80)
	P<0.001	P<0.001
**Moped risk**	-35.18 (-41.51, -28.85)	0.42 (0.35, 0.51)
	P<0.001	P<0.001
**Cycle helmet risk**	-5.41 (-10.93, 0.12)	0.79 (0.68, 0.94)
	P=0.055	P=0.007
**Drug/solvent use**	-23.65 (-36.29, -10.99)	0.81 (0.57, 1.14)
	P<0.001	p=0.225
**Cannabis use**	-20.59 (-28.76, -12.42)	0.71 (0.56, 0.89)
	P<0.001	P=0.003
**Tobacco smoking**	-57.40 (-63.86, -50.94)	0.30 (0.25, 0.36)
	P<0.001	P<0.001
**Hazardous alcohol use**	-5.56 (-10.69, -0.43)	0.91 (0.77, 1.06)
	P=0.034	P=0.228
**Combined sexual risk**	-28.57 (-34.42, -22.72)	0.55 (0.47, 0.65)
	P<0.001	P<0.001
**Self-harm**	-12.24 (-18.17, -6.31)	0.83 (0.70, 0.99)
	P<0.001	P=0.041

**Criminal/Antisocial behaviour**	-22.76 (-27.2, -18.32)	0.58 (0.50, 0.66)
	P<0.001	P<0.001
**Physical inactivity**	-0.24 (-5.80, 5.33)	1.10 (0.92, 1.31)
	P=0.934	P=0.284
**TV viewing**	-23.97 (-29.82, -18.11)	0.60 (0.50, 0.72)
	P<0.001	P<0.001
