# Supplementary material for: Effect of multiple risk behaviours in adolescence on educational attainment at age 16 years: a UK birth cohort study
Source: BMJ Open. 2018 Jul 30;8(7):e020182. doi: 10.1136/bmjopen-2017-020182 (PMC6067358; doi:10.1136/bmjopen-2017-020182)
Supplement: Author's manuscript [file bmjopen-2017-020182.draft_revisions.pdf]

BMJ Open is committed to open peer review. As part of this commitment we make the peer review history of every article we publish publicly available.

When an article is published we post the peer reviewers' comments and the authors' responses online. We also post the versions of the paper that were used during peer review. These are the versions that the peer review comments apply to.

The versions of the paper that follow are the versions that were submitted during the peer review process. They are not the versions of record or the final published versions. They should not be cited or distributed as the published version of this manuscript.

BMJ Open is an open access journal and the full, final, typeset and author-corrected version of record of the manuscript is available on our site with no access controls, subscription charges or pay-per-view fees (http://bmjopen.bmj.com).

If you have any questions on BMJ Open's open peer review process please email info.bmjopen@bmj.com

BMJ Open

The effect of multiple risk behaviours in adolescence on educational attainment at age 16 years: a UK birth cohort study

Journal:	*BMJ Open*
Manuscript ID	bmjopen-2017-020182
Article Type:	Research
Date Submitted by the Author:	18-Oct-2017

Complete List of Authors:	Wright, Caroline; University of Bristol, Bristol Medical School<br>Kipping, Ruth; University of Bristol, Bristol Medical School<br>Hickman, M; University of Bristol, Bristol Medical School<br>Campbell, Rona; University of Bristol, Bristol Medical School<br>Heron, Jon; University of Bristol, Bristol Medical School

**Primary Subject Heading**:	Epidemiology
Secondary Subject Heading:	Public health
Keywords:	multiple risk behaviours, adolescence, educational attainment, ALSPAC, UK birth cohort study

SCHOLARONE™ Manuscripts

The effect of multiple risk behaviours in adolescence on educational attainment at age 16 years: a UK birth cohort study

Caroline Wright,[1] Ruth Kipping,[1] Matthew Hickman,[1] Rona Campbell,[1] and Jon Heron.[1]

Correspondence to: Caroline Wright, Population Health Sciences, Bristol Medical School, University of Bristol, BF4, Barley House, Oakfield Grove, Bristol, BS8 2BN

caroline.wright@bristol.ac.uk or +44 (0)117 3314011

For peer review only

[1] Population Health Sciences, Bristol Medical School, University of Bristol, Oakfield House, Bristol, BS82BN, UK.

For peer review only - http://bmjopen.bmj.com/site/about/guidelines.xhtml

Abstract

Objectives: To explore the association between adolescent multiple risk behaviours (MRB) and educational attainment.

Design: Prospective population based UK birth cohort study.

Setting: Avon Longitudinal Study of Parents and Children (ALSPAC), a UK birth cohort of children born in 1991-92.

Participants: Data on some or all MRB measures were available for 5,401 ALSPAC participants who attended a clinic at age 15 years and/or completed a detailed questionnaire at age 16 years. Multiple imputation was used to account for missing data.

Primary outcome measures: Capped General Certificate of Secondary Education (GCSE) score and odds of attaining five or more GCSE examinations at grades A*-C. Both outcome measures come from the National Pupil Database (NPD) and were linked to the ALSPAC data.

Results: Engagement in MRB was strongly associated with poorer educational attainment. Each additional risk equated to -6.31 (95% CI -7.03, -5.58, p<0.001) in capped GCSE score, equivalent to a one grade reduction, or reduced odds of attaining five or more A*-C grades of 23% (OR 0·77, 95% CI: 0·74-0·81, p<0.001). The average cohort member engaged in 3·24 MRB and therefore have an associated reduction in GCSE score equivalent to three and a half grades in one examination, or reduced odds of attaining five or more A*-C grades of 75%.

Conclusion: Engagement in adolescent MRB is strongly associated with poorer educational attainment at 16 years. Preventing MRB could improve educational attainment and thereby directly and indirectly improve longer term health.

Keywords: multiple risk behaviours, adolescence, educational attainment, ALSPAC, UK birth cohort study

For peer review only - http://bmjopen.bmj.com/site/about/guidelines.xhtml

Strengths and limitations of this study

- This is the first longitudinal study in the UK to examine the association between multiple health risk behaviours and educational attainment.
- Owing to the scale and richness of the ALSPAC data, we have been able to control for a wide range of confounders, including socio-economic measures, previous educational attainment, and prior ability.
- The risk behaviours were all reduced to binary variables in order to construct the total MRB score, which leads to a loss of information.
- By summing the risk behaviours to create a measure of total MRBs, we attribute equal weight to each behaviour, however, we have found no strong evidence for an alternative classification of MRB in terms of clusters of specific MRBs or latent classes. [1]
- There is considerable missing data for the exposure and confounder variables which reduces power and may introduce bias, however, there is no missing data on either outcome measure, and although missingness is related to MRB our imputed analyses are similar to the complete case analysis.

Sources of funding

ALSPAC receives core support from the UK Medical Research Council and the Wellcome Trust (grant reference 092731) and the University of Bristol. This work was undertaken with the support of The Centre for the Development and Evaluation of Complex Interventions for Public Health Improvement (DECIPHer), a UKCRC Public Health Research Centre of Excellence. Joint funding (MR/KO232331/1) from the British Heart Foundation, Cancer Research UK, Economic and Social Research Council, Medical Research Council, the Welsh Government and the Wellcome Trust, under the auspices of the UK Clinical Research Collaboration, is gratefully acknowledged.

For peer review only - http://bmjopen.bmj.com/site/about/guidelines.xhtml

Competing interests

The authors declare that there are no competing interests.

Introduction

Health risk behaviours such as smoking, alcohol consumption, physical inactivity, and unhealthy eating are prevalent during adolescence [2,3] and have also been shown to co-occur during this period. [4-6] A growing body of evidence suggests that these behaviours are strongly associated, some causally, with adverse health outcomes in later life, including chronic health conditions, morbidity, and premature mortality. [6-8]

Successful completion of compulsory education is important to an individual's well-being and lifelong opportunities. Those with lower educational attainment are more likely to smoke,[9] be overweight, and have poor physical and mental health outcomes.[10] They also experience reduced employment opportunities and earning potential,[10-12] while successful completion of compulsory education is strongly associated with increased aspirations and life satisfaction[13] and those with college degrees or higher are the most likely to engage in healthy behaviours.[14]

Many studies consider the effects of single health risk behaviours and educational outcomes: obesity,[4,15] smoking tobacco,[5,16,17] using cannabis,[16-18] drinking alcohol,[5,17] self-harm,[19] physical inactivity,[20] and screen based behaviours involving TV, internet, or computer games[21] are all associated with poorer educational outcomes. Less studies consider multiple risk behaviours simultaneously,[2,22-24] fewer consider a large number of heterogeneous risk behaviours concurrently.[25-27] However, no UK studies have examined engagement in multiple risk behaviours and educational outcomes. Using data from the ALSPAC cohort in England, we aimed to investigate the association of multiple risk behaviours during adolescence and educational attainment at age 16.

For peer review only - http://bmjopen.bmj.com/site/about/guidelines.xhtml

Methods

Sample

Data were drawn from the Avon Longitudinal Study of Parents and Children (ALSPAC), an ongoing prospective observational population-based study investigating the effects of a wide range of influences on health and development across the life course. Pregnant women residing in the old administrative county of Avon, who had an estimated date of delivery between 1 April 1991 and 31 December 1992, were invited to participate. The initial study cohort consisted of 14,062 live born children of whom 13,988 singletons or twins were still alive at 12 months of age. A small number of participants withdrew from the study (n=24).[28] Those who were neither enrolled nor part of the original core ALSPAC sample were excluded from our analyses, along with any triplets or quadruplets whose identity, because of their rarity, would be compromised. Compared to the 1991 UK National Census data, the sample showed a slightly higher proportion of house owner-occupiers and a smaller proportion of mothers from ethnic minorities. Further, children enrolled in ALSPAC were on average more educated at 16 compared to the national average, more likely to be White (reflecting the ethnic composition of the area from which the sample was drawn) and less likely to claim eligibility for free school meals (an indicator of low income in the UK).[28] The study website contains details of all the data that are available through a fully searchable data dictionary: http://www.bris.ac.uk/alspac/researchers/data-access/data-dictionary/.[29] Ethical approval for the study was obtained from the ALSPAC Ethics and Law Committee and local Research Ethics Committees.

Linkage between ALSPAC and National Pupil Database

The National Pupil Database (NPD) is a pupil level data source which matches pupil and school characteristic data to pupil level attainment data in England. The Fischer Trust completed the linkage between the NPD and ALSPAC data in 2002. It is only mandatory for

For peer review only - http://bmjopen.bmj.com/site/about/guidelines.xhtml

schools following the National Curriculum to contribute to the NPD. Independent schools may provide attainment data on a voluntary basis.

Exposure measure

Multiple risk behaviours at age 16

Measures of participation in thirteen distinct risk behaviours at the ages of 15 and 16 years were derived from participants' responses at two ALSPAC data collections during their late teens (see Table 1). The first was a self-completed questionnaire issued during a clinic attended at age 15 (median age 15 years and 5 months) and the second comprised responses to a postal questionnaire administered at age 16 (median age 16 years and 7 months). These behaviours represent a breadth of domains of social and health risk including: sexual health, substance use, self-harm, vehicle related injury risk, criminal and antisocial behaviour (ASB), and physical inactivity. The derivation of each behaviour is discussed in more detail in an earlier paper.[22] For the purposes of the analyses reported here a total number of risk-behaviours score from 0 to 13 was derived for each participant.

Outcome measures

Educational attainment at age 16

Pupils in England aged between 14 and 16 complete compulsory schooling and take their GCSE (or equivalent) examinations at key stage four (KS4). At the time, the ALSPAC cohort were in school, UK law stated that pupils were to remain in compulsory education until the age of 16, so unlike A-Levels, GCSEs are one of very few occasions in a young person's life when their educational attainment is assessed along with most of their peers. Two outcomes relating to KS4 educational attainment were used in the analysis. Achieving five or more A*-C grades at GCSE was chosen because it is a minimum requirement for many post-16 education and training courses and as such represents an important threshold for young people to exceed. The second outcome takes the individual scores for each GCSE, which are

For peer review only - http://bmjopen.bmj.com/site/about/guidelines.xhtml

calculated as A*=58 through to G=16 and U=0. This general attainment score is then capped by choosing a pupil's eight best grades. Referred to as the capped GCSE score it is seen as preferable to a total GCSE score because: (i) it represents the same measure that is used in the published value-added school league tables, which have become an important measure of the quality of education provision; and (ii) this measure is considered fairer than the total (uncapped) score since it moderates the scores of pupils who score highly merely by taking more examinations.

Possible Confounders

We adjusted for a number of potential confounders: sex, month of birth, parental social class, maternal education, household income, housing tenure during pregnancy and claiming eligibility for a free school meal. We additionally controlled for IQ score at age eight years and key stage two educational attainment in order to account for reverse causality between early educational performance and engagement in MRBs. Analysis of confounders and both the exposure and outcomes variables was conducted and can be found in the supplementary material.

Missing data

Of the starting sample of 13,954 subjects (enrolled cohort, singletons and twins alive at one year): 2,618 (18·8%) did not have a linked education record for KS4 and were excluded from the analysis on that basis. There are a number of reasons for this type of missingness: they may not have the linked data from the national pupil database (NPD), the participant may have withheld consent, or the participant may have been attending a school that does not follow the National Curriculum i.e. an independent school. Independent school education is of particular interest in this case because of its prominence in Bristol and Avon. Between 2006 and 2009 (when the ALSPAC cohort would have taken their GCSE examinations), the percentage of pupils educated in independent schools in England remained stable at

For peer review only - http://bmjopen.bmj.com/site/about/guidelines.xhtml

approximately 7%. In Bristol, it ranged between 15·1% in 2006/07 and 13·4% in 2008/09. However, with no way of confirming that those with missing attainment and school type data were independently educated, and no alternative identifier of independent school status, we were unable to conduct a sensitivity analysis with this respect. Our sample analysis is therefore less representative of Bristol at the time, but more generalizable to the overall population, where independent schooling is less common. Of the 11,336 subjects with education outcome data, 5,401 had some or all of their MRB information and this became the imputation sample upon which our analysis is based. Additional analysis regarding those with complete data (n=1,617) is available in the supplementary material (see Figure 1).

Multivariate imputation by chained equations was carried out using the 'ice' routine in Stata version 14. This approach is based on the Missing At Random (MAR) assumption, i.e. that any differences between the missing and observed values, can be explained by differences in the observed data[30]. Indicators of socio-economic status, sex and prior measures of criminal and anti-social activity, substance use, and educational attainment were included in the imputation routine as auxiliary variables to reduce bias by improving the precision of the imputation model. Monte Carlo errors were used to compare the results obtained when imputing 25, 100, 250, and 500 data sets.[31] Imputed results shown have been pooled across the 500 data sets.

Sample descriptive statistics

Compared to the imputation sample the complete case sample had better educational outcomes, a higher mean IQ score and a lower mean total MRB score. The percentage of females, and all indicators of advantageous socio-economic position were over-represented in the complete case sample when compared with the imputation sample (see Table 2).

For peer review only - http://bmjopen.bmj.com/site/about/guidelines.xhtml

Associations between confounders and exposure variables

We found that lower maternal education, lower parental social class, living in privately rented or subsidised housing, having a lower income, and claiming eligibility for free school meals (FSM) were all positively associated with the total number of multiple risk behaviours at age 16. IQ at age 8 and higher previous educational attainment at key stage two were negatively associated with total MRB score. There was no association between sex, season of birth, or SEN and MRB score.

Lower maternal education, lower parental social class, living in privately rented or subsidised housing, having a lower income, and claiming eligibility for free school meals (FSM) were all associated with lower educational attainment at age 16. This was true for both educational outcomes. Being born in the spring or summer compared with the autumn was associated with lower capped GCSE score, but had no effect on gaining five or more A*-C grades at GCSE. Having higher previous attainment levels at key stage two and a higher IQ at age 8 were associated with better educational outcomes at age 16 and being female was associated with an improved grade point average equivalent to more than one and a half grades. Details of these analyses can be found in the supplementary material.

Statistical analysis

Linear regression was used to examine associations between total MRB and the continuous outcome, capped GCSE score and logistic regression was used for the binary outcome, gaining five or more GCSE grades A*-C. Analyses were conducted on the complete case and imputed data the latter of which constituted the main analysis. We ran unadjusted models for both outcomes followed by a sequence of models adjusted for: (i) sex and season of birth, (ii) sex, season of birth, maternal education, parental social class, household income, tenure and FSM, and (iii) sex, season of birth maternal education, parental social class, household income, tenure, FSM, IQ, and previous educational attainment. We tested for non-linearity

For peer review only - http://bmjopen.bmj.com/site/about/guidelines.xhtml

between exposure and outcome using the 'nlcheck' function in Stata, however, found no evidence that the linearity assumption had been violated. We also tested for interactions between sex and MRB; and each of the socio-economic indicators and MRB, in terms of their effect on educational outcomes, but found no strong evidence of any associations (p values range from 0·047 for housing tenure to 0·827 for FSM). All analyses were conducted in Stata version 14.

Results

Associations between total MRB and educational outcomes

In the ALSPAC cohort at age 16 years, the total number of multiple risk behaviours engaged in was strongly associated with lower educational attainment (see Table 3). In the unadjusted models, for every additional risk behaviour a young person engaged in, the capped GCSE score decreased on average 9·17 points, equivalent to a grade and a half in one GCSE examination. Similarly, the odds of attaining five or more A*-C grades were reduced by 18% for each additional risk behaviour.

The association between multiple risk behaviours and capped GCSE score did not change markedly with the inclusion of sex and season of birth in the models. However, a more substantial reduction in the association was seen with the inclusion of the socio-economic indicators (maternal education, parental social class, household income, housing tenure, and free school meal status). The final and fully adjusted model, which additionally included IQ at age 8 and previous educational attainment, shows that for each additional multiple risk behaviour, the participant will see a reduction in capped GCSE score of 6·31 points, which is equivalent to just more than one grade in one GCSE examination. The mean number of risk behaviours engaged in was 3·24, which means that on average young people from this cohort have a reduced GCSE score of 20·44 points, associated with their risk taking. This is

For peer review only - http://bmjopen.bmj.com/site/about/guidelines.xhtml

equivalent to a reduction of nearly three and a half grades in one GCSE examination, or one grade lower in three examinations.

The negative association between engaging in MRBs and gaining five or more GCSEs between A*-C, as demonstrated in the preliminary analysis, remained large in the adjusted models. Indeed, after adjusting for all the confounders, the magnitude of this effect increased and the odds of attaining five or more A*-C grades was reduced by 23% for each additional risk behaviour engaged in, although it should be noted that the confidence intervals do overlap with the previous model.

There were contrasting patterns of association between the separate risk behaviours and educational attainment. Smoking had the largest deleterious association and those who smoked scored an average of 57·40 capped GCSE points less than those who did not smoke. This would be equivalent to getting an ungraded U classification instead of an A* in one GCSE examination, or put differently getting one grade lower in nine separate GCSE examinations. Smokers were also 70% less likely to gain five or more A*-C grades at GCSE compared to non-smokers at age 16. Not wearing a cycle helmet, hazardous alcohol use, and physical inactivity had no association with educational attainment. Details of these analyses can be found in the supplementary material.

Discussion

In our analysis of the ALSPAC cohort, adolescents with a greater number of health risk behaviours had poorer educational outcomes at age 16. While the fully adjusted models for both the complete case and the imputed datasets showed some attenuation in the estimates for MRB with respect capped GCSE score, the effect remained strong despite adjusting for a wide range of confounders. The fully adjusted model showed a reduction in the association between capped GCSE score of 6.31 points, which would be equivalent to a reduction of

For peer review only - http://bmjopen.bmj.com/site/about/guidelines.xhtml

more than one grade in one GCSE examination, for each additional MRB engaged in. A similarly adverse association with a young person's odds of gaining five or more GCSEs between A*-C was also observed with the odds of attaining five or more A*-C grades at GCSE reduced by 23% for each additional risk behaviour. We also corroborated associations between a wide range of individual MRB and educational outcomes at age 16.

This is the first longitudinal study in the UK to examine the association between a multiple health risk behaviours and educational attainment. Current research in this area often considers single risk behaviours or small 'clusters' of risks, but none to our knowledge consider a large number, of heterogeneous risks simultaneously. Owing to the scale and richness of the ALSPAC data, we have been able to control for a wide range of confounders, including socio-economic measures, previous educational attainment, and prior ability.

However, there are several limitations to our analysis. Firstly, the risk behaviours were all reduced to binary variables in order to construct the total MRB score, which leads to a loss of information. It is possible that individual behaviours showing no relationship with educational attainment in this study would show associations if examined using a different classification of risk behaviour (e.g. hazardous alcohol use and physical inactivity), but we think it unlikely to have an impact on relationship between number of MRB and outcomes. Secondly, by summing the risk behaviours to create a measure of total MRBs, we attribute equal weight to each behaviour, however, we have found no strong evidence for an alternative classification of MRB in terms of clusters of specific MRBs or latent classes. Thirdly, there is considerable missing data on confounders and MRB which reduces power and may introduce bias. However, there is no missing data on either outcome measure, and although missingness is related to MRB our imputed analyses are similar to the complete case analysis and we do not expect there to be substantial bias.

For peer review only - http://bmjopen.bmj.com/site/about/guidelines.xhtml

The findings from this research build on and are consistent with current studies which have shown positive associations between single risk behaviours or small numbers of similar risks and poorer educational attainment [4,5,15-21,32-34]. They also echo findings from a US study which found strong evidence of associations between higher educational attainment and membership to the most-healthy cluster of adolescents. Although that study did not show the same dose-response relationship that we have found [14].

Engagement in multiple health risk behaviours during adolescence is strongly associated with lower educational attainment at age 16 years. Although we have not shown a causal association between exposure and outcome measures, our findings demonstrate for the first time that multiple health risk behaviours act as an important predictor of adverse educational outcomes, over and above a wide range of confounders including intellectual ability (IQ) at age 8 years and previous educational attainment. This finding could aid the identification and targeting of young people at risk of under-achieving during their compulsory education. Further, by showing a dose-response relationship between the two, we have shown the importance of intervening in and reducing each and every risk behaviour. Preventing multiple risk behaviours during adolescence could improve educational attainment and thereby directly and indirectly improve longer term health outcomes.

This research has not been able to establish the direction of the association between MRB and educational attainment, which would provide a valuable focus for future work in this area. Future work with this cohort would enable investigation of repeated measures of MRB at different time points throughout childhood and adolescence, in order to explore if there is any difference in the association between MRB and educational outcomes according to timing of MRB. Further research is also required to identify the early-life antecedents that are associated with adolescent MRB, as this would facilitate effective early intervention of those with the highest risk of engaging in harmful MRB.

For peer review only - http://bmjopen.bmj.com/site/about/guidelines.xhtml

Contributorship Statement

CW, RK, MH, RC and JH conceived and designed the study. CW and JH carried out the study, including acquiring and analysing the data. CW and JH interpreted the data. CW drafted the manuscript. RK, MH, RC and JH critiqued the manuscript for important intellectual content. All authors have read and approved the final version of the manuscript. CW serves as guarantor. MH and RC are senior investigators for the National Institute for Health Research. CW and JH had full access to all of the data and can take responsibility for the integrity of the data and the accuracy of the data analysis. CW, RK, MH, RC and JH had access to the statistical outputs, reports, and tables. All researchers are independent of the funding bodies.

Data sharing statement

The ALSPAC policy on data sharing is available at www.bristol.ac.uk/alspac. To discuss access to ALSPAC data, please contact the ALSPAC executive team on alspac-exec@bristol.ac.uk.

Acknowledgements

We are extremely grateful to all the families who took part in this study, the midwives for their help in recruiting them, and the whole ALSPAC team, which includes interviewers, computer and laboratory technicians, clerical workers, research scientists, volunteers, managers, receptionists and nurses. The UK Medical Research Council and the Wellcome Trust (Grant ref: 102215/2/13/2) and the University of Bristol provide core support for ALSPAC.

REFERENCES

1. Heron J, Campbell R, Hickman M, Kipping RR. Latent class analysis of multiple risk behaviour in adolescents in the ALSPAC cohort. *BMC Public Health* under review.
2. de Winter AF, Leenke V, Verhulst FC, Vollebergh WAM, Reijneveld SA. Longitudinal patterns and predictors of multiple health risk behaviors among adolescents: The TRAILS study. *Prev Med* 2015; **84**: 76-82.
3. Mays D, Peshkin BN, Sharff ME, et al. Correlates of adherence to a telephone-based multiple health behavior change cancer preventive intervention for teens: the Healthy for Life Program (HELP). *Health Education and Behavior* 2012; **39**(1): 18-26.
4. Booth JN, Tomporowski PD, Boyle JME, et al. Obesity impairs academic attainment in adolescence: findings from ALSPAC, a UK cohort. *Int J Obes (Lond)* 2014; **38**(10): 1335-42.
5. Latvala A, Rose RJ, Pulkkinen L, Dick DM, Korhonen T, Kaprio J. Drinking, smoking, and educational achievement: cross-lagged associations from adolescence to adulthood. *Drug and Alcohol Dependence* 2014; **137**: 106-13.
6. McCullough ML, Patel AV, Kushi LH, et al. Following cancer prevention guidelines reduces risk of cancer, cardiovascular disease, and all-cause mortality. *Cancer Epidemiology, Biomarkers & Prevention* 2011; **20**(6): 1089-97.
7. Kvaavik E, Batty D, Ursin G, Huxley R, Gale C. Influence of Individual and Combined Health Behaviors on Total and Cause-Specific Mortality in Men and Women. *Archives of Internal Medicine* 2010; **170**(8): 711-8.
8. Connor J. Alcohol consumption as a cause of cancer. *Addiction* 2016; **112**(2): 222–8.
9. Gilman SE, Martin LT, Abrams DB, et al. Educational attainment and cigarette smoking: a causal association? *Int J Epidemiol* 2008; **37**(3): 615-24.
10. Marmot M. Fair society healthy lives full report. 2010.
11. Ward JL, Viner RM. Secondary Education and Health Outcomes in Young People from the Cape Area Panel Study (CAPS). *PLoS One* 2016; **11**(6): 1-14.
12. ONS. Full Report - Graduates in the UK Labour. 2013.
13. Education Df. Youth Cohort Study & Longitudinal study of young people in England: The Activities and Experiences of 19 year olds: England 2010. In: Education Df, editor.; 2011.
14. Skalamera J, Hummer RA. Educational attainment and the clustering of health-related behavior among U.S. young adults. *Prev Med* 2016; **84**: 83-9.
15. Caird J, Kavanagh J, Oliver K, et al. Childhood obesity and educational attainment a systematic review. London: EPPI-Centre, Social Science Research Unit, Institute of Education, University of London: Institute of Education, University of London, 2011.
16. Stiby AI, Hickman M, Munafo MR, Heron J, Yip VL, Macleod J. Adolescent cannabis and tobacco use and educational outcomes at age 16: birth cohort study. *Addiction* 2015; **110**(4): 658-68.
17. Silins E, Fergusson DM, Patton GC, et al. Adolescent substance use and educational attainment: An integrative data analysis comparing cannabis and alcohol from three Australasian cohorts. *Drug and Alcohol Dependence* 2015; **156**: 90-6.
18. Verweij KJ, Huizink AC, Agrawal A, Martin NG, Lynskey MT. Is the relationship between early-onset cannabis use and educational attainment causal or due to common liability? *Drug and Alcohol Dependence* 2013; **133**(2): 580-6.
19. Mars B, Heron J, Crane C, et al. Clinical and social outcomes of adolescent self harm: population based birth cohort study. *BMJ* 2014; **349**: 1-13.

For peer review only - http://bmjopen.bmj.com/site/about/guidelines.xhtml

20. Booth JN, Leary SD, Joinson C, et al. Associations between objectively measured physical activity and academic attainment in adolescents from a UK cohort. *British Journal of Sports Medicine* 2014; **48**(3): 265-70.
21. Corder K, Atkin AJ, Bamber DJ, et al. Revising on the run or studying on the sofa: prospective associations between physical activity, sedentary behaviour, and exam results in British adolescents. *International Journal of Behavioral Nutrition and Physical Activity* 2015; **12**: 106.
22. Kipping RR, Smith M, Heron J, Hickman M, Campbell R. Multiple risk behaviour in adolescence and socio-economic status: findings from a UK birth cohort. *European Journal of Public Health* 2015; **25**(1): 44-9.
23. Alzahrani SG, Watt RG, Sheiham A, Aresu M, Tsakos G. Patterns of clustering of six health-compromising behaviours in Saudi adolescents. *BMC Public Health* 2014; **14**: 1215.
24. de Looze M, Ter Bogt TFM, Raaijmakers QAW, Pickett W, Kuntsche E, Vollebergh WAM. Cross-national evidence for the clustering and psychosocial correlates of adolescent risk behaviours in 27 countries. *European Journal of Public Health* 2015; **25**(1): 50-6.
25. Busch V, Van Stel HF, Schrijvers AJP, de Leeuw JRJ. Clustering behaviours outcomes Dutch adolescents. *BMC Public Health* 2013; **13**: 1-11.
26. Noel H, Denny S, Farrant B, et al. Clustering of adolescent health concerns: a latent class analysis of school students in New Zealand. *Journal of Paediatrics and Child Health* 2013; **49**(11): 935-41.
27. van Nieuwenhuijzen M, Junger M, Velderman MK, et al. Clustering of health-compromising behavior and delinquency in adolescents and adults in the Dutch population. *Prev Med* 2009; **48**(6): 572-8.
28. Boyd A, Golding J, Macleod J, et al. Cohort Profile: the 'children of the 90s'-the index offspring of the Avon Longitudinal Study of Parents and Children. *Int J Epidemiol* 2013; **42**(1): 111-27.
29. ALSPAC. Avon Longitudinal Study of Parents and Children. ALSPAC: Data Dictionary. 2016.
30. Sterne J, White J, Carlin J, et al. Multiple imputation for missing data in epidemiological and clinical research: potential and pitfalls. *BMJ* 2009; **338**: b2393.
31. White IR, Royston P, Wood AM. Multiple imputation using chained equations: Issues and guidance for practice. *Stat Med* 2011; **30**(4): 377-99.
32. Grant JD, Scherrer JF, Lynskey MT, et al. Associations of alcohol, nicotine, cannabis, and drug use/dependence with educational attainment: evidence from cotwin-control analyses. *Alcohol Clin Exp Res* 2012; **36**(8): 1412-20.
33. Horwood LJ, Fergusson DM, Hayatbakhsh MR, et al. Cannabis use and educational achievement: findings from three Australasian cohort studies. *Drug Alcohol Depend* 2010; **110**(3): 247-53.
34. Staff J, Patrick ME, Loken E, Maggs J. Teenage Alcohol Use and Educational Attainment. *Journal of Studies on Alcohol and Drugs* 2008; **69**(6): 848-58.

For peer review only - http://bmjopen.bmj.com/site/about/guidelines.xhtml

Table 1: Multiple risk behaviours (MRB) and their derivation

Health risk behaviour	Definition / how derived
Physical inactivity	Young person (YP) has typically over the past year exercised <5 times per week.
TV viewing	YP spent 3 or more hours watching television on average per day across the week.
Car passenger risk	YP had been in a car passenger at least once in their lifetime where the driver (a) had consumed alcohol or (b) did not have a valid licence, or (c) the YP chose not to wear a seat belt last time travelled in a car, van, or taxi.
Cycle helmet use	If the YP reported that they had last ridden a bicycle within the previous four weeks and they had not worn a helmet on the most recent occasion.
Scooter risk	YP has driven a motorbike/ scooter off road, or without a licence on a public road at least once.
Criminal/Antisocial behaviour	YP reported that at least once in the past year they had undertaken at least one of the following 7 offences: carried a weapon; physically hurt someone on purpose; stolen something; sold illicit substances to another person; damaged property belonging to someone else either by using graffiti, setting fire to it or destroying or damaging it in another fashion; subjected someone to verbal or physical racial abuse; or been rude/rowdy in a public place.
Hazardous alcohol consumption	In the past year had scored eight or more on the Alcohol Use Disorders Identification Test (AUDIT) indicating hazardous alcohol consumption.
Regular tobacco smoking	Has ever smoked and is regularly smoking by currently smoking at least one cigarette per week.

For peer review only - http://bmjopen.bmj.com/site/about/guidelines.xhtml

Cannabis use	Those who reported using cannabis "sometimes but less often than once a week" or more regular use were classified as occasional users.
Illicit drug/solvent use	In the year since their 15th birthday, YP had either been a regular user (i.e. used five or more times) of one or more illicit drugs (excluding cannabis) including amphetamines, ecstasy, LSD, cocaine, ketamine or inhalants including aerosols, gas, solvents and poppers.
Self-harm	Young people who said they had purposely hurt themselves in some way in their lifetime.
Penetrative sex before age 16	YP reported having had penetrative sex in the preceding year and that they were under 16 at the time.
Unprotected sex	Penetrative sex without the use of contraception on the last occasion they had had sex in the past year

Sources of information:

Age 15 years clinic: criminal and antisocial behaviour, penetrative sex prior to age 16, and unprotected sex.

Age 16 years questionnaire: physical inactivity, TV viewing, car passenger risk, cycle helmet use, scooter risk, hazardous alcohol drinking, regular smoking, illicit drug/solvent use, and self-harm.

For peer review only

For peer review only - http://bmjopen.bmj.com/site/about/guidelines.xhtml

Table 2: Sample descriptive statistics

		Imputation sample	Complete case sample
		**5,401**	**1,617**
		**% (SE)**	**n (%)**
Educational Outcomes	n=5,401		
Capped GCSE score: Mean (SE)		350 (1·01)	376 (1·31)
Five or more A*-C grades at GCSE		74·4% (SE=0·6)	1420 (87·8%)
Four or less A*-C grades at GCSE		25·6% (SE=0·6)	197 (12·2%)
Exposure	n=2,264		
MRB total: Mean (SE)		3.24 (0·31)	3.01 (0·05)
Sex	n=5,401		
Female		55·5% (SE=0·7)	975 (60·3%)
Male		44·5% (SE=0·7)	642 (39·7%
Season of birth	n=5,401		
Autumn		33·9% (SE=0·6)	542 (33·5%)
Winter		14·8% (SE=0·5)	222 (13·7%)
Spring		23·4% (SE=0·6)	382 (23·6%)
Summer		27·9% (SE=0·6)	471 (29·1%)
Maternal education	n=5,214		
Degree		14·9% (SE=0·5)	311 (19·2%)
A level		25·9% (SE=0·6)	479 (29·6%)
O level		34·3% (SE=0·7)	596 (36·9%)
< O level		21·5% (SE=0·6)	231 (14·3%)
Parental socio-economic position	n=4,970		
Professional		14·1% (SE=0·5)	291 (18·0%)
Managerial and technical		41·2% (SE=0·7)	761 (47·1%)

For peer review only - http://bmjopen.bmj.com/site/about/guidelines.xhtml

Skilled non-man		23·6% (SE=0·6)	398 (24·6%)
Skilled man, part or unskilled		13·2% (SE=0·5)	167 (10·3%)
Housing tenure	n=5,227		
Mortgage/owned		84·7% (SE=0·5)	1.444 (89·3%)
Private rent		7·3% (SE=0·4)	88 (5·4%)
Subsidised rent		8·6% (SE=0·4)	85 (5·3%)
Income	n=4,809		
High		20.86% (SE=0·6)	364 (22·5%)
Mid high		21.96% (SE=0·6)	424 (26·2%)
Middle		21.34% (SE=0·6)	364 (22·5%)
Mid low		19.25% (SE=0·6)	292 (18·1%)
Low		16.58% (SE=0·5)	173 (10·7%)
Free School Meals (FSM)	n=5,401		
Ever FSM		7·7% (SE=0·4)	65 (4·0%)
Never FSM		92·3% (SE=0·4)	1,552 (96·0%)
Special Educational Needs (SEN)	n=5,075		
No SEN		84·7% (SE=0·4)	1,522 (94·1%)
School Action		6·0% (SE=0·4)	68 (4·2%)
School Action Plus		1·9% (SE=0·2)	18 (1·1%)
Statement of SEN		1·4% (SE=0·2)	9 (0·6%)
Previous educational attainment/ability			
IQ at age 8: Mean (SE)	n=4,370	105 (0·24)	109 (0·37)
KS2 educational attainment: Mean (SE)	n=4,753	830 (2·76)	901 (3·94)

For peer review only - http://bmjopen.bmj.com/site/about/guidelines.xhtml

Table 3: Associations between total MRB score and capped GCSE score and odds of gaining five or more GCSEs at grade A*-C

	Unadjusted (n=5,401)	Model 1 (n=5,401)	Model 2 (n=5,401)	Model 3 (n=5,401)
**Capped GCSE**	-9·17 (-10·25, -8·10) p<0·001	-9·12 (-10·19, -8·05) p<0·001	-6·90 (-7·86, -5·94) p<0·001	-6·31 (-7·03, -5·58) p<0·001
**5 A*-C**	0·82 (0·79-0·85) p<0·001	0·82 (0·79-0·85) p<0·001	0·84 (0·81-0·87) p<0·001	0·77 (0·74-0·81) p<0·001

Model 1: adjusted for sex and season of birth

Model 2: adjusted for sex, season of birth maternal education, parental social class, household income, tenure, and FSM.

Model 3: adjusted for sex, season of birth maternal education, parental social class, household income, tenure, FSM, IQ, and previous educational attainment.

For peer review only - http://bmjopen.bmj.com/site/about/guidelines.xhtml

The effect of multiple risk behaviours in adolescence on educational attainment at age 16 years: a UK birth cohort study

Caroline Wright,[1] Ruth Kipping,[1] Matthew Hickman,[1] Rona Campbell,[1] and Jon Heron.[1]

Correspondence to: Caroline Wright, Population Health Sciences, Bristol Medical School, University of Bristol, BF4, Barley House, Oakfield Grove, Bristol, BS8 2BN

caroline.wright@bristol.ac.uk or +44 (0)117 3314011

Formatted: Foo color: Auto

Formatted: Foo color: Auto

Formatted: Foo color: Auto

Formatted: Foo color: Auto

For peer review only

[1] Population Health Sciences, Bristol Medical School, University of Bristol, Oakfield House, Bristol, BS82BN, UK.

Abstract

Objectives: To explore the association between adolescent multiple risk behaviours (MRB) and educational attainment.

Design: Prospective population based UK birth cohort study.

Setting: Avon Longitudinal Study of Parents and Children (ALSPAC), a UK birth cohort of children born in 1991-92.

Participants: Data on some or all MRB measures were available for 5,401 ALSPAC participants who attended a clinic at age 15 years and/or completed a detailed questionnaire at age 16 years. Multiple imputation was used to account for missing data.

Primary outcome measures: Capped General Certificate of Secondary Education (GCSE) score and odds of attaining five or more GCSE examinations at grades A*-C. Both outcome measures come from the National Pupil Database (NPD) and were linked to the ALSPAC data.

Results: Engagement in MRB was strongly associated with poorer educational attainment. Each additional risk equated to -6.31 (95% CI -7.03, -5.58, p<0.001) in capped GCSE score, equivalent to a one grade reduction, or reduced odds of attaining five or more A*-C grades of 23% (OR 0·77, 95% CI: 0·74-0·81, p<0.001). The average cohort member engaged in 3·24 MRB and therefore have an associated reduction in GCSE score equivalent to three and a half grades in one examination, or reduced odds of attaining five or more A*-C grades of 75%.

Conclusion: Engagement in adolescent MRB is strongly associated with poorer educational attainment at 16 years. Preventing MRB could improve educational attainment and thereby directly and indirectly improve longer term health.

Keywords: multiple risk behaviours, adolescence, educational attainment, ALSPAC, UK birth cohort study

For peer review only - http://bmjopen.bmj.com/site/about/guidelines.xhtml

Strengths and limitations of this study

- This is the first longitudinal study in the UK to examine the association between multiple health risk behaviours and educational attainment.
- Owing to the scale and richness of the ALSPAC data, we have been able to control for a wide range of confounders, including socio-economic measures, previous educational attainment, and prior ability.
- The risk behaviours were all reduced to binary variables in order to construct the total MRB score, which leads to a loss of information.
- By summing the risk behaviours to create a measure of total MRBs, we attribute equal weight to each behaviour, however, we have found no strong evidence for an alternative classification of MRB in terms of clusters of specific MRBs or latent classes. [1]
- There is considerable missing data for the exposure and confounder variables which reduces power and may introduce bias, however, there is no missing data on either outcome measure, and although missingness is related to MRB our imputed analyses are similar to the complete case analysis.

Sources of funding

ALSPAC receives core support from the UK Medical Research Council and the Wellcome Trust (grant reference 092731) and the University of Bristol. This work was undertaken with the support of The Centre for the Development and Evaluation of Complex Interventions for Public Health Improvement (DECIPHer), a UKCRC Public Health Research Centre of Excellence. Joint funding (MR/KO232331/1) from the British Heart Foundation, Cancer Research UK, Economic and Social Research Council, Medical Research Council, the Welsh Government and the Wellcome Trust, under the auspices of the UK Clinical Research Collaboration, is gratefully acknowledged.

For peer review only - http://bmjopen.bmj.com/site/about/guidelines.xhtml

Competing interests

The authors declare that there are no competing interests.

Introduction

Health risk behaviours such as smoking, alcohol consumption, physical inactivity, and unhealthy eating are prevalent during adolescence [2,3] and have also been shown to co-occur during this period. [4-6] A growing body of evidence suggests that these behaviours are strongly associated, some causally, with adverse health outcomes in later life, including chronic health conditions, morbidity, and premature mortality. [6-8]

Successful completion of compulsory education is important to an individual's well-being and lifelong opportunities. Those with lower educational attainment are more likely to smoke,[9] be overweight, and have poor physical and mental health outcomes.[10] They also experience reduced employment opportunities and earning potential,[10-12] while successful completion of compulsory education is strongly associated with increased aspirations and life satisfaction[13] and those with college degrees or higher are the most likely to engage in healthy behaviours.[14]

Many studies consider the effects of single health risk behaviours and educational outcomes: obesity,[4,15] smoking tobacco,[5,16,17] using cannabis,[16-18] drinking alcohol,[5,17] self-harm,[19] physical inactivity,[20] and screen based behaviours involving TV, internet, or computer games[21] are all associated with poorer educational outcomes. Less studies consider multiple risk behaviours simultaneously,[2,22-24] fewer consider a large number of heterogeneous risk behaviours concurrently.[25-27] However, no UK studies have examined engagement in multiple risk behaviours and educational outcomes. Using data from the ALSPAC cohort in England, we aimed to investigate the association of multiple risk behaviours during adolescence and educational attainment at age 16.

For peer review only - http://bmjopen.bmj.com/site/about/guidelines.xhtml

Methods

Sample

Data were drawn from the Avon Longitudinal Study of Parents and Children (ALSPAC), an ongoing prospective observational population-based study investigating the effects of a wide range of influences on health and development across the life course. Pregnant women residing in the old administrative county of Avon, who had an estimated date of delivery between 1 April 1991 and 31 December 1992, were invited to participate. The initial study cohort consisted of 14,062 live born children of whom 13,988 singletons or twins were still alive at 12 months of age. A small number of participants withdrew from the study (n=24).[28] Those who were neither enrolled nor part of the original core ALSPAC sample were excluded from our analyses, along with any triplets or quadruplets whose identity, because of their rarity, would be compromised. Compared to the 1991 UK National Census data, the sample showed a slightly higher proportion of house owner-occupiers and a smaller proportion of mothers from ethnic minorities. Further, children enrolled in ALSPAC were on average more educated at 16 compared to the national average, more likely to be White (reflecting the ethnic composition of the area from which the sample was drawn) and less likely to claim eligibility for free school meals (an indicator of low income in the UK).[28] The study website contains details of all the data that are available through a fully searchable data dictionary: http://www.bris.ac.uk/alspac/researchers/data-access/data-dictionary/.[29] Ethical approval for the study was obtained from the ALSPAC Ethics and Law Committee and local Research Ethics Committees.

Linkage between ALSPAC and National Pupil Database

The National Pupil Database (NPD) is a pupil level data source which matches pupil and school characteristic data to pupil level attainment data in England. The Fischer Trust completed the linkage between the NPD and ALSPAC data in 2002. It is only mandatory for

For peer review only - http://bmjopen.bmj.com/site/about/guidelines.xhtml

schools following the National Curriculum to contribute to the NPD. Independent schools may provide attainment data on a voluntary basis.

Exposure measure

Multiple risk behaviours at age 16

Measures of participation in thirteen distinct risk behaviours at the ages of 15 and 16 years were derived from participants' responses at two ALSPAC data collections during their late teens (see Table 1). The first was a self-completed questionnaire issued during a clinic attended at age 15 (median age 15 years and 5 months) and the second comprised responses to a postal questionnaire administered at age 16 (median age 16 years and 7 months). These behaviours represent a breadth of domains of social and health risk including: sexual health, substance use, self-harm, vehicle related injury risk, criminal and antisocial behaviour (ASB), and physical inactivity. The derivation of each behaviour is discussed in more detail in an earlier paper.[22] For the purposes of the analyses reported here a total number of risk-behaviours score from 0 to 13 was derived for each participant.

Outcome measures

Educational attainment at age 16

Pupils in England aged between 14 and 16 complete compulsory schooling and take their GCSE (or equivalent) examinations at key stage four (KS4). At the time, the ALSPAC cohort were in school, UK law stated that pupils were to remain in compulsory education until the age of 16, so unlike A-Levels, GCSEs are one of very few occasions in a young person's life when their educational attainment is assessed along with most of their peers. Two outcomes relating to KS4 educational attainment were used in the analysis. Achieving five or more A*-C grades at GCSE was chosen because it is a minimum requirement for many post-16 education and training courses and as such represents an important threshold for young people to exceed. The second outcome takes the individual scores for each GCSE, which are

For peer review only - http://bmjopen.bmj.com/site/about/guidelines.xhtml

calculated as A*=58 through to G=16 and U=0. This general attainment score is then capped by choosing a pupil's eight best grades. Referred to as the capped GCSE score it is seen as preferable to a total GCSE score because: (i) it represents the same measure that is used in the published value-added school league tables, which have become an important measure of the quality of education provision; and (ii) this measure is considered fairer than the total (uncapped) score since it moderates the scores of pupils who score highly merely by taking more examinations.

Possible Confounders

We adjusted for a number of potential confounders: sex, month of birth, parental social class, maternal education, household income, housing tenure during pregnancy and claiming eligibility for a free school meal. We additionally controlled for IQ score at age eight years and key stage two educational attainment in order to account for reverse causality between early educational performance and engagement in MRBs. Analysis of confounders and both the exposure and outcomes variables was conducted and can be found in the supplementary material.

Missing data

Of the starting sample of 13,954 subjects (enrolled cohort, singletons and twins alive at one year): 2,618 (18·8%) did not have a linked education record for KS4 and were excluded from the analysis on that basis. There are a number of reasons for this type of missingness: they may not have the linked data from the national pupil database (NPD), the participant may have withheld consent, or the participant may have been attending a school that does not follow the National Curriculum i.e. an independent school. Independent school education is of particular interest in this case because of its prominence in Bristol and Avon. Between 2006 and 2009 (when the ALSPAC cohort would have taken their GCSE examinations), the percentage of pupils educated in independent schools in England remained stable at

For peer review only - http://bmjopen.bmj.com/site/about/guidelines.xhtml

approximately 7%. In Bristol, it ranged between 15·1% in 2006/07 and 13·4% in 2008/09. However, with no way of confirming that those with missing attainment and school type data were independently educated, and no alternative identifier of independent school status, we were unable to conduct a sensitivity analysis with this respect. Our sample analysis is therefore less representative of Bristol at the time, but more generalizable to the overall population, where independent schooling is less common. Of the 11,336 subjects with education outcome data, 5,401 had some or all of their MRB information and this became the imputation sample upon which our analysis is based. Additional analysis regarding those with complete data (n=1,617) is available in the supplementary material (see Figure 1).

Multivariate imputation by chained equations was carried out using the 'ice' routine in Stata version 14. This approach is based on the Missing At Random (MAR) assumption, i.e. that any differences between the missing and observed values, can be explained by differences in the observed data[30]. Indicators of socio-economic status, sex and prior measures of criminal and anti-social activity, substance use, and educational attainment were included in the imputation routine as auxiliary variables to reduce bias by improving the precision of the imputation model. Monte Carlo errors were used to compare the results obtained when imputing 25, 100, 250, and 500 data sets.[31] Imputed results shown have been pooled across the 500 data sets.

Sample descriptive statistics

Compared to the imputation sample the complete case sample had better educational outcomes, a higher mean IQ score and a lower mean total MRB score. The percentage of females, and all indicators of advantageous socio-economic position were over-represented in the complete case sample when compared with the imputation sample (see Table 2).

For peer review only - http://bmjopen.bmj.com/site/about/guidelines.xhtml

Associations between confounders and exposure variables

We found that lower maternal education, lower parental social class, living in privately rented or subsidised housing, having a lower income, and claiming eligibility for free school meals (FSM) were all positively associated with the total number of multiple risk behaviours at age 16. IQ at age 8 and higher previous educational attainment at key stage two were negatively associated with total MRB score. There was no association between sex, season of birth, or SEN and MRB score.

Lower maternal education, lower parental social class, living in privately rented or subsidised housing, having a lower income, and claiming eligibility for free school meals (FSM) were all associated with lower educational attainment at age 16. This was true for both educational outcomes. Being born in the spring or summer compared with the autumn was associated with lower capped GCSE score, but had no effect on gaining five or more A*-C grades at GCSE. Having higher previous attainment levels at key stage two and a higher IQ at age 8 were associated with better educational outcomes at age 16 and being female was associated with an improved grade point average equivalent to more than one and a half grades. Details of these analyses can be found in the supplementary material.

Statistical analysis

Linear regression was used to examine associations between total MRB and the continuous outcome, capped GCSE score and logistic regression was used for the binary outcome, gaining five or more GCSE grades A*-C. Analyses were conducted on the complete case and imputed data the latter of which constituted the main analysis. We ran unadjusted models for both outcomes followed by a sequence of models adjusted for: (i) sex and season of birth, (ii) sex, season of birth, maternal education, parental social class, household income, tenure and FSM, and (iii) sex, season of birth maternal education, parental social class, household income, tenure, FSM, IQ, and previous educational attainment. We tested for non-linearity

For peer review only - http://bmjopen.bmj.com/site/about/guidelines.xhtml

between exposure and outcome using the 'nlcheck' function in Stata, however, found no evidence that the linearity assumption had been violated. We also tested for interactions between sex and MRB; and each of the socio-economic indicators and MRB, in terms of their effect on educational outcomes, but found no strong evidence of any associations (p values range from 0·047 for housing tenure to 0·827 for FSM). All analyses were conducted in Stata version 14.

Results

Associations between total MRB and educational outcomes

In the ALSPAC cohort at age 16 years, the total number of multiple risk behaviours engaged in was strongly associated with lower educational attainment (see Table 3). In the unadjusted models, for every additional risk behaviour a young person engaged in, the capped GCSE score decreased on average 9·17 points, equivalent to a grade and a half in one GCSE examination. Similarly, the odds of attaining five or more A*-C grades were reduced by 18% for each additional risk behaviour.

The association between multiple risk behaviours and capped GCSE score did not change markedly with the inclusion of sex and season of birth in the models. However, a more substantial reduction in the association was seen with the inclusion of the socio-economic indicators (maternal education, parental social class, household income, housing tenure, and free school meal status). The final and fully adjusted model, which additionally included IQ at age 8 and previous educational attainment, shows that for each additional multiple risk behaviour, the participant will see a reduction in capped GCSE score of 6·31 points, which is equivalent to just more than one grade in one GCSE examination. The mean number of risk behaviours engaged in was 3·24, which means that on average young people from this cohort have a reduced GCSE score of 20·44 points, associated with their risk taking. This is

For peer review only

For peer review only - http://bmjopen.bmj.com/site/about/guidelines.xhtml

equivalent to a reduction of nearly three and a half grades in one GCSE examination, or one grade lower in three examinations.

The negative association between engaging in MRBs and gaining five or more GCSEs between A*-C, as demonstrated in the preliminary analysis, remained large in the adjusted models. Indeed, after adjusting for all the confounders, the magnitude of this effect increased and the odds of attaining five or more A*-C grades was reduced by 23% for each additional risk behaviour engaged in, although it should be noted that the confidence intervals do overlap with the previous model.

There were contrasting patterns of association between the separate risk behaviours and educational attainment. Smoking had the largest deleterious association and those who smoked scored an average of 57·40 capped GCSE points less than those who did not smoke. This would be equivalent to getting an ungraded U classification instead of an A* in one GCSE examination, or put differently getting one grade lower in nine separate GCSE examinations. Smokers were also 70% less likely to gain five or more A*-C grades at GCSE compared to non-smokers at age 16. Not wearing a cycle helmet, hazardous alcohol use, and physical inactivity had no association with educational attainment. Details of these analyses can be found in the supplementary material.

Discussion

In our analysis of the ALSPAC cohort, adolescents with a greater number of health risk behaviours had poorer educational outcomes at age 16. While the fully adjusted models for both the complete case and the imputed datasets showed some attenuation in the estimates for MRB with respect capped GCSE score, the effect remained strong despite adjusting for a wide range of confounders. The fully adjusted model showed a reduction in the association between capped GCSE score of 6.31 points, which would be equivalent to a reduction of

For peer review only - http://bmjopen.bmj.com/site/about/guidelines.xhtml
For peer review only

more than one grade in one GCSE examination, for each additional MRB engaged in. A similarly adverse association with a young person's odds of gaining five or more GCSEs between A*-C was also observed with the odds of attaining five or more A*-C grades at GCSE reduced by 23% for each additional risk behaviour. We also corroborated associations between a wide range of individual MRB and educational outcomes at age 16.

This is the first longitudinal study in the UK to examine the association between a multiple health risk behaviours and educational attainment. Current research in this area often considers single risk behaviours or small 'clusters' of risks, but none to our knowledge consider a large number, of heterogeneous risks simultaneously. Owing to the scale and richness of the ALSPAC data, we have been able to control for a wide range of confounders, including socio-economic measures, previous educational attainment, and prior ability. However, there are several limitations to our analysis. Firstly, the risk behaviours were all reduced to binary variables in order to construct the total MRB score, which leads to a loss of information. It is possible that individual behaviours showing no relationship with educational attainment in this study would show associations if examined using a different classification of risk behaviour (e.g. hazardous alcohol use and physical inactivity), but we think it unlikely to have an impact on relationship between number of MRB and outcomes. Secondly, by summing the risk behaviours to create a measure of total MRBs, we attribute equal weight to each behaviour, however, we have found no strong evidence for an alternative classification of MRB in terms of clusters of specific MRBs or latent classes. Thirdly, there is considerable missing data on confounders and MRB which reduces power and may introduce bias. However, there is no missing data on either outcome measure, and although missingness is related to MRB our imputed analyses are similar to the complete case analysis and we do not expect there to be substantial bias.

For peer review only - http://bmjopen.bmj.com/site/about/guidelines.xhtml

The findings from this research build on and are consistent with current studies which have shown positive associations between single risk behaviours or small numbers of similar risks and poorer educational attainment [4,5,15-21,32-34]. They also echo findings from a US study which found strong evidence of associations between higher educational attainment and membership to the most-healthy cluster of adolescents. Although that study did not show the same dose-response relationship that we have found [14].

Engagement in multiple health risk behaviours during adolescence is strongly associated with lower educational attainment at age 16 years. Although we have not shown a causal association between exposure and outcome measures, our findings demonstrate for the first time that multiple health risk behaviours act as an important predictor of adverse educational outcomes, over and above a wide range of confounders including intellectual ability (IQ) at age 8 years and previous educational attainment. This finding could aid the identification and targeting of young people at risk of under-achieving during their compulsory education. Further, by showing a dose-response relationship between the two, we have shown the importance of intervening in and reducing each and every risk behaviour. Preventing multiple risk behaviours during adolescence could improve educational attainment and thereby directly and indirectly improve longer term health outcomes.

This research has not been able to establish the direction of the association between MRB and educational attainment, which would provide a valuable focus for future work in this area. Future work with this cohort would enable investigation of repeated measures of MRB at different time points throughout childhood and adolescence, in order to explore if there is any difference in the association between MRB and educational outcomes according to timing of MRB. Further research is also required to identify the early-life antecedents that are associated with adolescent MRB, as this would facilitate effective early intervention of those with the highest risk of engaging in harmful MRB.

For peer review only - http://bmjopen.bmj.com/site/about/guidelines.xhtml

Contributorship Statement

CW, RK, MH, RC and JH conceived and designed the study. CW and JH carried out the study, including acquiring and analysing the data. CW and JH interpreted the data. CW drafted the manuscript. RK, MH, RC and JH critiqued the manuscript for important intellectual content. All authors have read and approved the final version of the manuscript. CW serves as guarantor. MH and RC are senior investigators for the National Institute for Health Research. CW and JH had full access to all of the data and can take responsibility for the integrity of the data and the accuracy of the data analysis. CW, RK, MH, RC and JH had access to the statistical outputs, reports, and tables. All researchers are independent of the funding bodies.

Data sharing statement

The ALSPAC policy on data sharing is available at www.bristol.ac.uk/alspac. To discuss access to ALSPAC data, please contact the ALSPAC executive team on alspac-exec@bristol.ac.uk.

Acknowledgements

We are extremely grateful to all the families who took part in this study, the midwives for their help in recruiting them, and the whole ALSPAC team, which includes interviewers, computer and laboratory technicians, clerical workers, research scientists, volunteers, managers, receptionists and nurses. The UK Medical Research Council and the Wellcome Trust (Grant ref: 102215/2/13/2) and the University of Bristol provide core support for ALSPAC.

For peer review only - http://bmjopen.bmj.com/site/about/guidelines.xhtml

REFERENCES

1. Heron J, Campbell R, Hickman M, Kipping RR. Latent class analysis of multiple risk behaviour in adolescents in the ALSPAC cohort. *BMC Public Health* under review.
2. de Winter AF, Leenke V, Verhulst FC, Vollebergh WAM, Reijneveld SA. Longitudinal patterns and predictors of multiple health risk behaviors among adolescents: The TRAILS study. *Prev Med* 2015; **84**: 76-82.
3. Mays D, Peshkin BN, Sharff ME, et al. Correlates of adherence to a telephone-based multiple health behavior change cancer preventive intervention for teens: the Healthy for Life Program (HELP). *Health Education and Behavior* 2012; **39**(1): 18-26.
4. Booth JN, Tomporowski PD, Boyle JME, et al. Obesity impairs academic attainment in adolescence: findings from ALSPAC, a UK cohort. *Int J Obes (Lond)* 2014; **38**(10): 1335-42.
5. Latvala A, Rose RJ, Pulkkinen L, Dick DM, Korhonen T, Kaprio J. Drinking, smoking, and educational achievement: cross-lagged associations from adolescence to adulthood. *Drug and Alcohol Dependence* 2014; **137**: 106-13.
6. McCullough ML, Patel AV, Kushi LH, et al. Following cancer prevention guidelines reduces risk of cancer, cardiovascular disease, and all-cause mortality. *Cancer Epidemiology, Biomarkers & Prevention* 2011; **20**(6): 1089-97.
7. Kvaavik E, Batty D, Ursin G, Huxley R, Gale C. Influence of Individual and Combined Health Behaviors on Total and Cause-Specific Mortality in Men and Women. *Archives of Internal Medicine* 2010; **170**(8): 711-8.
8. Connor J. Alcohol consumption as a cause of cancer. *Addiction* 2016; **112**(2): 222–8.
9. Gilman SE, Martin LT, Abrams DB, et al. Educational attainment and cigarette smoking: a causal association? *Int J Epidemiol* 2008; **37**(3): 615-24.
10. Marmot M. Fair society healthy lives full report. 2010.
11. Ward JL, Viner RM. Secondary Education and Health Outcomes in Young People from the Cape Area Panel Study (CAPS). *PLoS One* 2016; **11**(6): 1-14.
12. ONS. Full Report - Graduates in the UK Labour. 2013.
13. Education Df. Youth Cohort Study & Longitudinal study of young people in England: The Activities and Experiences of 19 year olds: England 2010. In: Education Df, editor.; 2011.
14. Skalamera J, Hummer RA. Educational attainment and the clustering of health-related behavior among U.S. young adults. *Prev Med* 2016; **84**: 83-9.
15. Caird J, Kavanagh J, Oliver K, et al. Childhood obesity and educational attainment a systematic review. London: EPPI-Centre, Social Science Research Unit, Institute of Education, University of London: Institute of Education, University of London, 2011.
16. Stiby AI, Hickman M, Munafo MR, Heron J, Yip VL, Macleod J. Adolescent cannabis and tobacco use and educational outcomes at age 16: birth cohort study. *Addiction* 2015; **110**(4): 658-68.
17. Silins E, Fergusson DM, Patton GC, et al. Adolescent substance use and educational attainment: An integrative data analysis comparing cannabis and alcohol from three Australasian cohorts. *Drug and Alcohol Dependence* 2015; **156**: 90-6.
18. Verweij KJ, Huizink AC, Agrawal A, Martin NG, Lynskey MT. Is the relationship between early-onset cannabis use and educational attainment causal or due to common liability? *Drug and Alcohol Dependence* 2013; **133**(2): 580-6.
19. Mars B, Heron J, Crane C, et al. Clinical and social outcomes of adolescent self harm: population based birth cohort study. *BMJ* 2014; **349**: 1-13.

For peer review only - http://bmjopen.bmj.com/site/about/guidelines.xhtml

20. Booth JN, Leary SD, Joinson C, et al. Associations between objectively measured physical activity and academic attainment in adolescents from a UK cohort. *British Journal of Sports Medicine* 2014; **48**(3): 265-70.

21. Corder K, Atkin AJ, Bamber DJ, et al. Revising on the run or studying on the sofa: prospective associations between physical activity, sedentary behaviour, and exam results in British adolescents. *International Journal of Behavioral Nutrition and Physical Activity* 2015; **12**: 106.

22. Kipping RR, Smith M, Heron J, Hickman M, Campbell R. Multiple risk behaviour in adolescence and socio-economic status: findings from a UK birth cohort. *European Journal of Public Health* 2015; **25**(1): 44-9.

23. Alzahrani SG, Watt RG, Sheiham A, Aresu M, Tsakos G. Patterns of clustering of six health-compromising behaviours in Saudi adolescents. *BMC Public Health* 2014; **14**: 1215.

24. de Looze M, Ter Bogt TFM, Raaijmakers QAW, Pickett W, Kuntsche E, Vollebergh WAM. Cross-national evidence for the clustering and psychosocial correlates of adolescent risk behaviours in 27 countries. *European Journal of Public Health* 2015; **25**(1): 50-6.

25. Busch V, Van Stel HF, Schrijvers AJP, de Leeuw JRJ. Clustering behaviours outcomes Dutch adolescents. *BMC Public Health* 2013; **13**: 1-11.

26. Noel H, Denny S, Farrant B, et al. Clustering of adolescent health concerns: a latent class analysis of school students in New Zealand. *Journal of Paediatrics and Child Health* 2013; **49**(11): 935-41.

27. van Nieuwenhuijzen M, Junger M, Velderman MK, et al. Clustering of health-compromising behavior and delinquency in adolescents and adults in the Dutch population. *Prev Med* 2009; **48**(6): 572-8.

28. Boyd A, Golding J, Macleod J, et al. Cohort Profile: the 'children of the 90s'-the index offspring of the Avon Longitudinal Study of Parents and Children. *Int J Epidemiol* 2013; **42**(1): 111-27.

29. ALSPAC. Avon Longitudinal Study of Parents and Children. ALSPAC: Data Dictionary. 2016.

30. Sterne J, White J, Carlin J, et al. Multiple imputation for missing data in epidemiological and clinical research: potential and pitfalls. *BMJ* 2009; **338**: b2393.

31. White IR, Royston P, Wood AM. Multiple imputation using chained equations: Issues and guidance for practice. *Stat Med* 2011; **30**(4): 377-99.

32. Grant JD, Scherrer JF, Lynskey MT, et al. Associations of alcohol, nicotine, cannabis, and drug use/dependence with educational attainment: evidence from cotwin-control analyses. *Alcohol Clin Exp Res* 2012; **36**(8): 1412-20.

33. Horwood LJ, Fergusson DM, Hayatbakhsh MR, et al. Cannabis use and educational achievement: findings from three Australasian cohort studies. *Drug Alcohol Depend* 2010; **110**(3): 247-53.

34. Staff J, Patrick ME, Loken E, Maggs J. Teenage Alcohol Use and Educational Attainment. *Journal of Studies on Alcohol and Drugs* 2008; **69**(6): 848-58.

For peer review only - http://bmjopen.bmj.com/site/about/guidelines.xhtml

Table 1: Multiple risk behaviours (MRB) and their derivation

**Health risk behaviour**	**Definition / how derived**
Physical inactivity	Young person (YP) has typically over the past year exercised <5 times per week.
TV viewing	YP spent 3 or more hours watching television on average per day across the week.
Car passenger risk	YP had been in a car passenger at least once in their lifetime where the driver (a) had consumed alcohol or (b) did not have a valid licence, or (c) the YP chose not to wear a seat belt last time travelled in a car, van, or taxi.
Cycle helmet use	If the YP reported that they had last ridden a bicycle within the previous four weeks and they had not worn a helmet on the most recent occasion.
Scooter risk	YP has driven a motorbike/ scooter off road, or without a licence on a public road at least once.
Criminal/Antisocial behaviour	YP reported that at least once in the past year they had undertaken at least one of the following 7 offences: carried a weapon; physically hurt someone on purpose; stolen something; sold illicit substances to another person; damaged property belonging to someone else either by using graffiti, setting fire to it or destroying or damaging it in another fashion; subjected someone to verbal or physical racial abuse; or been rude/rowdy in a public place.
Hazardous alcohol consumption	In the past year had scored eight or more on the Alcohol Use Disorders Identification Test (AUDIT) indicating hazardous alcohol consumption.
Regular tobacco smoking	Has ever smoked and is regularly smoking by currently smoking at least one cigarette per week.

For peer review only

For peer review only - http://bmjopen.bmj.com/site/about/guidelines.xhtml

Cannabis use	Those who reported using cannabis "sometimes but less often than once a week" or more regular use were classified as occasional users.
Illicit drug/solvent use	In the year since their 15th birthday, YP had either been a regular user (i.e. used five or more times) of one or more illicit drugs (excluding cannabis) including amphetamines, ecstasy, LSD, cocaine, ketamine or inhalants including aerosols, gas, solvents and poppers.
Self-harm	Young people who said they had purposely hurt themselves in some way in their lifetime.
Penetrative sex before age 16	YP reported having had penetrative sex in the preceding year and that they were under 16 at the time.
Unprotected sex	Penetrative sex without the use of contraception on the last occasion they had had sex in the past year

Sources of information:

Age 15 years clinic: criminal and antisocial behaviour, penetrative sex prior to age 16, and unprotected sex.

Age 16 years questionnaire: physical inactivity, TV viewing, car passenger risk, cycle helmet use, scooter risk, hazardous alcohol drinking, regular smoking, illicit drug/solvent use, and self-harm.

For peer review only

For peer review only - http://bmjopen.bmj.com/site/about/guidelines.xhtml

Table 2: Sample descriptive statistics

		Imputation sample	Complete case sample
		**5,401**	**1,617**
		**% (SE)**	**n (%)**
Educational Outcomes	n=5,401		
Capped GCSE score: Mean (SE)		350 (1·01)	376 (1·31)
Five or more A*-C grades at GCSE		74·4% (SE=0·6)	1420 (87·8%)
Four or less A*-C grades at GCSE		25·6% (SE=0·6)	197 (12·2%)
Exposure	n=2,264		
MRB total: Mean (SE)		3.24 (0·31)	3.01 (0·05)
Sex	n=5,401		
Female		55·5% (SE=0·7)	975 (60·3%)
Male		44·5% (SE=0·7)	642 (39·7%
Season of birth	n=5,401		
Autumn		33·9% (SE=0·6)	542 (33·5%)
Winter		14·8% (SE=0·5)	222 (13·7%)
Spring		23·4% (SE=0·6)	382 (23·6%)
Summer		27·9% (SE=0·6)	471 (29·1%)
Maternal education	n=5,214		
Degree		14·9% (SE=0·5)	311 (19·2%)
A level		25·9% (SE=0·6)	479 (29·6%)
O level		34·3% (SE=0·7)	596 (36·9%)
< O level		21·5% (SE=0·6)	231 (14·3%)
Parental socio-economic position	n=4,970		
Professional		14·1% (SE=0·5)	291 (18·0%)
Managerial and technical		41·2% (SE=0·7)	761 (47·1%)

For peer review only - http://bmjopen.bmj.com/site/about/guidelines.xhtml

Skilled non-man		23·6% (SE=0·6)	398 (24·6%)
Skilled man, part or unskilled		13·2% (SE=0·5)	167 (10·3%)
Housing tenure	n=5,227		
Mortgage/owned		84·7% (SE=0·5)	1.444 (89·3%)
Private rent		7·3% (SE=0·4)	88 (5·4%)
Subsidised rent		8·6% (SE=0·4)	85 (5·3%)
Income	n=4,809		
High		20.86% (SE=0·6)	364 (22·5%)
Mid high		21.96% (SE=0·6)	424 (26·2%)
Middle		21.34% (SE=0·6)	364 (22·5%)
Mid low		19.25% (SE=0·6)	292 (18·1%)
Low		16.58% (SE=0·5)	173 (10·7%)
Free School Meals (FSM)	n=5,401		
Ever FSM		7·7% (SE=0·4)	65 (4·0%)
Never FSM		92·3% (SE=0·4)	1,552 (96·0%)
Special Educational Needs (SEN)	n=5,075		
No SEN		84·7% (SE=0·4)	1,522 (94·1%)
School Action		6·0% (SE=0·4)	68 (4·2%)
School Action Plus		1·9% (SE=0·2)	18 (1·1%)
Statement of SEN		1·4% (SE=0·2)	9 (0·6%)
Previous educational attainment/ability			
IQ at age 8: Mean (SE)	n=4,370	105 (0·24)	109 (0·37)
KS2 educational attainment: Mean (SE)	n=4,753	830 (2·76)	901 (3·94)

For peer review only

Table 3: Associations between total MRB score and capped GCSE score and odds of gaining five or more GCSEs at grade A*-C

	Unadjusted (n=5,401)	Model 1 (n=5,401)	Model 2 (n=5,401)	Model 3 (n=5,401)
**Capped GCSE**	-9·17 (-10·25, -8·10) p<0·001	-9·12 (-10·19, -8·05) p<0·001	-6·90 (-7·86, -5·94) p<0·001	-6·31 (-7·03, -5·58) p<0·001
**5 A*-C**	0·82 (0·79-0·85) p<0·001	0·82 (0·79-0·85) p<0·001	0·84 (0·81-0·87) p<0·001	0·77 (0·74-0·81) p<0·001

Model 1: adjusted for sex and season of birth

Model 2: adjusted for sex, season of birth maternal education, parental social class, household income, tenure, and FSM.

Model 3: adjusted for sex, season of birth maternal education, parental social class, household income, tenure, FSM, IQ, and previous educational attainment.

For peer review only - http://bmjopen.bmj.com/site/about/guidelines.xhtml

Dear Hemali,

Many thanks for your email.

We received three suggestions for revisions from JECH, as outlined below:

Major revisions:
1. The association between MRB and capped GSCE score is assumed to be a monotonic linear function, but have the authors considered a polynomial regression to test for non-linear association? I suggest performing an analysis that includes a quadratic function of the MRB variable to assess for this.

2. Several potential confounders associate with both the exposure and outcome. Is there any evidence for interaction between these confounders and the exposure?

Minor revisions:
1. Please include the sequence of regression models in the statistical analysis section of Methods (ie: an unadjusted model followed by sequential adjustment for covariates)

In our view there was no theoretical justification for the inclusion of either a non-linear term in the model (between exposure and outcome) or for the testing of interactions between the confounders and the exposure, which is why neither were included in the original analysis.

However, we have subsequently tested for non-linearity between exposure and outcome using the nlcheck function in Stata and found no evidence that the linearity assumption had been violated (see track changes on page 9 and 10 of attached document).

We also subsequently tested for interactions between sex and MRB; and each of the socio-economic indicators (parental social class, maternal education, household income, housing tenure during pregnancy and claiming eligibility for a free school meal) and MRB, but again, found no evidence for any interactions - this is described on page 10 as part of the statistical analysis, but is not shown as a track change, because we had already incorporated this change into the paper.

Finally, we have included the sequence of regression models in the statistical analysis section, which can now be found as a track change on page 9.

I hope this is all clear, but please do let me know if you should have any queries.

With very best wishes,

Caroline.

Dr Caroline Wright
Research Associate

Population Health Sciences
Bristol Medical School
University of Bristol

BF4, Barley House
Oakfield Grove
Bristol, BS8 2BN
Tel: +44(0)117 3314011
email: caroline.wright@bristol.ac.uk

For peer review only

Advice Leading to Public Health Advancement
ALPHA
DECIPHer's research advisory group of young people

DECIPHer
Development and Evaluation of Complex Interventions for Public Health Improvement
A UKCRC Public Health Research Centre of Excellence

ALPHA Registration and Consent

This form requests consent for young people to be part of ALPHA, to use information and digital media resulting from ALPHA work, and to obtain an emergency contact.

What is ALPHA?

ALPHA - Advice Leading to Public Health Advancement is an advisory group of young people aged 14-22. As a member of ALPHA young people will receive training on public health and social care research to advise on research projects. Their involvement will mean young people will be expected to travel to meetings/events (costs covered by DECIPHer) when needed and a residential will be held once a year, which young people will be informed of well in advance.

What information and digital media do we collect?

We collect information and digital media from and about the young people in ALPHA. We use this information internally to continually improve the functioning of the group, and externally to promote the group and report to funders. Young people will not be named or identifiable in any outputs. This includes:

- Number of meetings and other events young people attend.
- Any other work the young people have completed through ALPHA such as blog writing and attending conferences.
- Requests young people make to staff - for example references for jobs.
- Written reports of the meetings.
- Young people's assessments of the quality of the sessions with researchers.
- Audio recordings, photographs and video of ALPHA work.

This information is kept secure in DECIPHer. Electronic data such as the audio files are kept on a computer in a password protected file that only staff associated with ALPHA have access to. Any data on paper, such as consent forms, are kept in a locked cupboard at DECIPHer which again only staff associated with ALPHA have access to. Any information used will retain the anonymity of young people, so they will not be named or be able to be identified.

ALPHA member registration

Young Person's name ……………………………….Date of Birth…………………

Home Address ……………………………………………………………………………

……………………………………………………………………………………………

Contact number for young person …………………………………………………

E-mail address for young person ……………………………………………………

For peer review only

Advice Leading to Public Health Advancement
ALPHA
DECIPHer's research advisory group of young people

DECIPHer
Development and Evaluation of Complex Interventions for Public Health Improvement
A UKCRC Public Health Research Centre of Excellence

School /College University (if applicable)……………………………………………

Please specify how we should contact the young person
- □ Letter via Post
- □ Text Message
- □ E-mail
- □ Facebook

Please report any allergies/conditions/additional needs you/your child has (including dietary as we organise lunch during the sessions).
………………………………………………………………………………………

………………………………………………………………………………………

Emergency Contact
Please note this information will only be used to advise the contact about an emergency situation. For those **under 16** this should be your next of kin. For those **16 or over** this contact can be a family member, friend or other as you deem appropriate.

Contact Name ……………………………………………………………………….

Relationship to young person ………………………………………………………

Contact number ……………………………………………………………………..

Home Address ………………………………………………………………………

………………………………………………………………………………………

Consent
If you are 16 or over you can sign the form yourself although we still need details of a person to contact in an emergency. If you are under 16 then a parent/guardian will need to sign on your behalf.

I hereby grant DECIPHer the right to use the information (detailed above) resulting from the ALPHA work, and any reproductions or adaptations of these materials for all general purposes in relation to DECIPHer's work including, without limitation, the right to use them in any publicity materials, books, newspapers and magazine articles whenever DECIPHer chooses to do so.

Signature………………………………………………………… Date…………..
If you have any questions about this consent from please feel free to contact Peter Gee, Public Involvement Officer for DECIPHer, Cardiff University, Tel: 029 20687213 or 07881514874 or e-mail: geep@cardiff.ac.uk

For peer review only

STROBE Statement—Checklist of items that should be included in reports of ***cohort studies***

	Item No	Recommendation
**Title and abstract**	1	(*a*) Indicate the study's design with a commonly used term in the title or the abstract
**Pages 1 &2**		(*b*) Provide in the abstract an informative and balanced summary of what was done and what was found
**Introduction**		
Background/rationale **Page 4**	2	Explain the scientific background and rationale for the investigation being reported
Objectives **Page 4**	3	State specific objectives, including any prespecified hypotheses
**Methods**		
Study design **Page 5**	4	Present key elements of study design early in the paper
Setting **Page 5**	5	Describe the setting, locations, and relevant dates, including periods of recruitment, exposure, follow-up, and data collection
Participants	6	(*a*) Give the eligibility criteria, and the sources and methods of selection of participants. Describe methods of follow-up
**Page 4**		(*b*) For matched studies, give matching criteria and number of exposed and unexposed
Variables **Page 6&7**	7	Clearly define all outcomes, exposures, predictors, potential confounders, and effect modifiers. Give diagnostic criteria, if applicable
Data sources/ measurement **Pages 5-7**	8*	For each variable of interest, give sources of data and details of methods of assessment (measurement). Describe comparability of assessment methods if there is more than one group
Bias **Pages 7&8**	9	Describe any efforts to address potential sources of bias
Study size **Page 7&8**	10	Explain how the study size was arrived at
Quantitative variables **Pages 6-8**	11	Explain how quantitative variables were handled in the analyses. If applicable, describe which groupings were chosen and why
Statistical methods	12	(*a*) Describe all statistical methods, including those used to control for confounding
		(*b*) Describe any methods used to examine subgroups and interactions
**Pages 5-9**		(*c*) Explain how missing data were addressed
		(*d*) If applicable, explain how loss to follow-up was addressed
		(*e*) Describe any sensitivity analyses
**Results**		
Participants	13*	(a) Report numbers of individuals at each stage of study—eg numbers potentially eligible, examined for eligibility, confirmed eligible, included in the study, completing follow-up, and analysed
**Pages 7&8**		(b) Give reasons for non-participation at each stage
		(c) Consider use of a flow diagram
Descriptive data	14*	(a) Give characteristics of study participants (eg demographic, clinical, social) and information on exposures and potential confounders
**Page 10**		(b) Indicate number of participants with missing data for each variable of interest
		(c) Summarise follow-up time (eg, average and total amount)
Outcome data **Pages 5, 7**	15*	Report numbers of outcome events or summary measures over time

For peer review only - http://bmjopen.bmj.com/site/about/guidelines.xhtml

Main results<br><br>**Pages 12-14**	16	(*a*) Give unadjusted estimates and, if applicable, confounder-adjusted estimates and their precision (eg, 95% confidence interval). Make clear which confounders were adjusted for and why they were included
		(*b*) Report category boundaries when continuous variables were categorized
		(*c*) If relevant, consider translating estimates of relative risk into absolute risk for a meaningful time period
Other analyses<br>**Pages 9**	17	Report other analyses done—eg analyses of subgroups and interactions, and sensitivity analyses
**Discussion**		
Key results<br>**Page 10&11**	18	Summarise key results with reference to study objectives
Limitations<br>**Pages 3 & 12**	19	Discuss limitations of the study, taking into account sources of potential bias or imprecision. Discuss both direction and magnitude of any potential bias
Interpretation<br>**Pages 11&12**	20	Give a cautious overall interpretation of results considering objectives, limitations, multiplicity of analyses, results from similar studies, and other relevant evidence
Generalisability<br>**Page 8**	21	Discuss the generalisability (external validity) of the study results
**Other information**		
Funding<br>**Page 3**	22	Give the source of funding and the role of the funders for the present study and, if applicable, for the original study on which the present article is based

*Give information separately for exposed and unexposed groups.

Note: An Explanation and Elaboration article discusses each checklist item and gives methodological background and published examples of transparent reporting. The STROBE checklist is best used in conjunction with this article (freely available on the Web sites of PLoS Medicine at http://www.plosmedicine.org/, Annals of Internal Medicine at http://www.annals.org/, and Epidemiology at http://www.epidem.com/). Information on the STROBE Initiative is available at http://www.strobe-statement.org.

For peer review only

Supplementary material table 1: Assessment for confounding, univariable associations with the exposure and outcome variables

	MRBs	Capped GCSE	Five or more GCSEs A*-C
**Gender ref: male**			
Female	-0.06 (-0.18, 0.06) p=0.343	10.83 (6.85, 14.81) p<0.001	1.32 (1.17, 1.49) p<0.001
**Season of birth ref: autumn**			
Winter	-0.04 (-0.23, 0.15) p=0.700	-4.18 (-10.35, 1.99) p=0.184	0.95 (0.79, 1.16) p=0.637
Spring	0.04 (-0.13, 0.20) p=0.674	-10.37 (-15.69, -5.05) p<0.001	0.78 (0.67, 0.92) p=0.004
Summer	0.26 (-0.13, 0.18) p=0.744	-13.34 (-18.40, -8.28) p<0.001	0.83 (0.71, 0.97) p=0.018
**Maternal education ref: degree**			
A level	0.17 (-0.02, 0.36) p=0.074	-35.19 (-41.13, -29.25) p<0.001	0.18 (0.12, 0.27) p<0.001
O level	0.41 (0.23, 0.59) p<0.001	-57.89 (-63.55, -52.23) p<0.001	0.09 (0.06, 0.14) p<0.001
< O level	0.49 (0.28, 0.69) p<0.001	-90.81 (-96.95, -84.68) p<0.001	0.04 (0.25, 0.06) p<0.001
**Parental socio-economic position ref: professional**			
Managerial and technical	0.43 (0.25, 0.61) p<0.001	-35.76 (-41.46, -30.05) p<0.001	0.29 (0.21, 0.39) p<0.001
Skilled non-man	0.47 (0.28, 0.67) p<0.001	-58.39 (-64.63, -52.15) p<0.001	0.15 (0.11, 0.21) p<0.001
Skilled man, part or unskilled	0.83 (0.60, 1.05) p<0.001	-88.71 (-95.76, -81.64) p<0.001	0.07 (0.05, 0.09) p<0.001

For peer review only

**Housing tenure ref: mortgage/owned**			
Private rent	0 .38 (0.14, 0.61) p=0.002	-20.07 (-27.50, -12.64) p<0.001	0.59 (0.47, 0.74) p<0.001
Subsidised rent	0 .75 (0.53, 0.97) p<0.001	-71.86 (-78.71, -65.01) p<0.001	0.18 (0.15, 0.74) p<0.001
**Income ref: high**			
Mid high	0.05 (-0.14, 0.24) p=0.615	-12.17 (-18.09, -6.26) p<0.001	0.62 (0.47, 0.80) p<0.001
Middle	0.17 (-0.02, 0.36) p=0.084	-32.02 (-37.98, -26.07) p<0.001	0.34 (0.26, 0.43) p<0.001
Mid low	0.35 (0.15, 0.55) p<0.001	-45.62 (-51.78, -39.46) p<0.001	0.23 (0.18, 0.29) p<0.001
Low	0.43 (0.23, 0.64) p<0.001	-74.31 (-80.75, -67.87) p<0.001	0.13 (0.10, 0.16) p<0.001
**FSM ref: never FSM**			
Ever FSM	0.97 (0.74, 1.21) p<0.001	-68.52 (-75.74, -61.30) p<0.001	0.22 (0.18, 0.27) p<0.001
**IQ at age 8**	-.0009 (-.0010, -.0006) p=0.009	2.69 (2.58, 2.79) p<0.001	1.09 (1.09, 1.10) p<0.001
**KS2 educational attainment**	-0.01 (-0.01, -0.01) p<0.001	0.26 (0.259, 0 .278) p<0.001	1.01 (1.01, 1.01) p<0.001
**SEN ref: no SEN**			
School Action	0.76 (-0.18, 0.33) p=0.555	-64.63 (-73.46, -55.80) p<0.001	0.19 (0.15, 0.25) p<0.001
School Action Plus	0.30 (-0.16, 0.77) p=0.201	-82.35 (-97.93, -66.78) p<0.001	0.13 (0.09, 0.21) p<0.001
Statement of SEN	-0.39 (-0.90, 0.12) p=0.134	-133.76 (-152.96, -114.56) p<0.001	0.11 (0.06, 0.19) p<0.001

For peer review only

Supplementary material table 2: Associations between total MRB score and educational outcomes, capped GCSE score and gaining five or more GCSEs at grade A*-C (complete case analysis)

	Complete Case Unadjusted (n=1,617)	Complete Case Adjusted[1] (n=1,617)	Complete Case Adjusted[2] (n=1,617)	Complete Case Adjusted[3] (n=1,617)
**Capped GCSE**	-6.12 (-7.42, -4.83) p<0.001	-6.13 (-7.42, -4.84) p<0.001	-4.82 (-6.00, -3.64) p<0.001	-4.48 (-5.31, -3.64) p<0.001
**5 A*-C**	0.81 (0.76–0.87) p<0.001	0.81 (0.75–0.87) p<0.001	0.84 (0.77-0.90) p<0.001	0.75 (0.68–0.83) p<0.001

[1] Adjusted for sex and season of birth.
[2] Adjusted for sex, season of birth maternal education, parental social class, household income, tenure and FSM.
[3] Adjusted for sex, season of birth maternal education, parental social class, household income, tenure FSM, IQ at age 8 and previous educational attainment at key stage 2.

For peer review only

Supplementary material table 3: Associations between separate MRB and educational outcomes, capped GCSE score and gaining five or more GCSEs at grade A*-C

	Capped GCSE score	Five or more GCSEs A*-C
**Car passenger risk**	-19.90 (-25.13, -14.67)<br>P<0.001	0.68 (0.58, 0.80)<br>P<0.001
**Moped risk**	-35.18 (-41.51, -28.85)<br>P<0.001	0.42 (0.35, 0.51)<br>P<0.001
**Cycle helmet risk**	-5.41 (-10.93, 0.12)<br>P=0.055	0.79 (0.68, 0.94)<br>P=0.007
**Drug/solvent use**	-23.65 (-36.29, -10.99)<br>P<0.001	0.81 (0.57, 1.14)<br>p=0.225
**Cannabis use**	-20.59 (-28.76, -12.42)<br>P<0.001	0.71 (0.56, 0.89)<br>P=0.003
**Tobacco smoking**	-57.40 (-63.86, -50.94)<br>P<0.001	0.30 (0.25, 0.36)<br>P<0.001
**Hazardous alcohol use**	-5.56 (-10.69, -0.43)<br>P=0.034	0.91 (0.77, 1.06)<br>P=0.228
**Combined sexual risk**	-28.57 (-34.42, -22.72)<br>P<0.001	0.55 (0.47, 0.65)<br>P<0.001
**Self-harm**	-12.24 (-18.17, -6.31)<br>P<0.001	0.83 (0.70, 0.99)<br>P=0.041

For peer review only

**Criminal/Antisocial behaviour**	-22.76 (-27.2, -18.32)	0.58 (0.50, 0.66)
	P<0.001	P<0.001
**Physical inactivity**	-0.24 (-5.80, 5.33)	1.10 (0.92, 1.31)
	P=0.934	P=0.284
**TV viewing**	-23.97 (-29.82, -18.11)	0.60 (0.50, 0.72)
	P<0.001	P<0.001

For peer review only

Figure 1: Deriving the sample

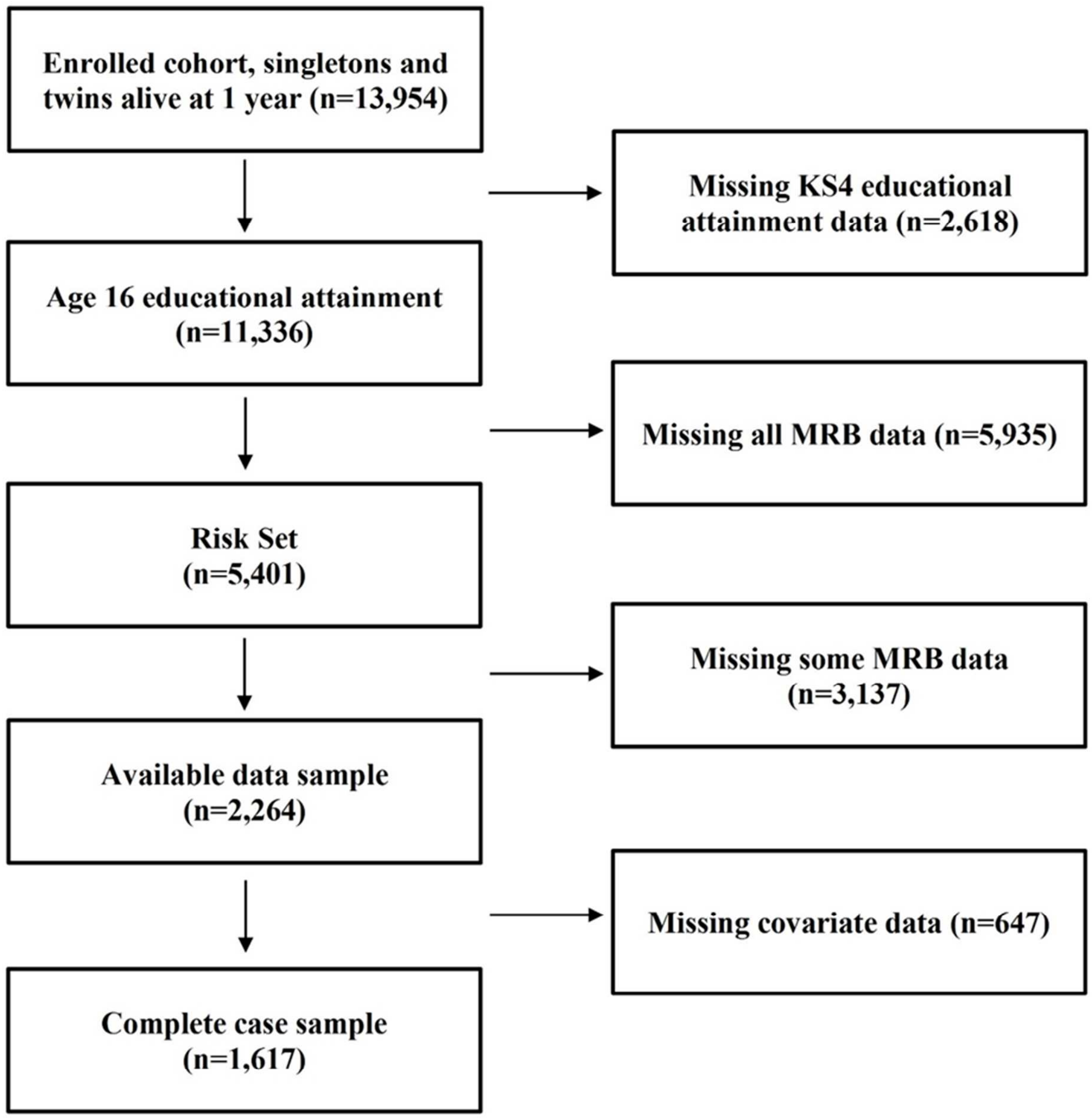

BMJ Open

The effect of multiple risk behaviours in adolescence on educational attainment at age 16 years: a UK birth cohort study

Journal:	*BMJ Open*
Manuscript ID	bmjopen-2017-020182.R1
Article Type:	Research
Date Submitted by the Author:	23-Mar-2018
Complete List of Authors:	Wright, Caroline; University of Bristol, Bristol Medical School<br>Kipping, Ruth; University of Bristol, Bristol Medical School<br>Hickman, M; University of Bristol, Bristol Medical School<br>Campbell, Rona; University of Bristol, Bristol Medical School<br>Heron, Jon; University of Bristol, Bristol Medical School
**Primary Subject Heading**:	Epidemiology
Secondary Subject Heading:	Public health
Keywords:	multiple risk behaviours, adolescence, educational attainment, ALSPAC, UK birth cohort study

SCHOLARONE™ Manuscripts

The effect of multiple risk behaviours in adolescence on educational attainment at age 16 years: a UK birth cohort study

Caroline Wright,[1] Ruth Kipping,[1] Matthew Hickman,[1] Rona Campbell,[1] and Jon Heron.[1]

Correspondence to: Caroline Wright, Population Health Sciences, Bristol Medical School, University of Bristol, BF4, Barley House, Oakfield Grove, Bristol, BS8 2BN

caroline.wright@bristol.ac.uk or +44 (0)117 3314011

For peer review only

[1] Population Health Sciences, Bristol Medical School, University of Bristol, Oakfield House, Bristol, BS82BN, UK.

For peer review only - http://bmjopen.bmj.com/site/about/guidelines.xhtml

Abstract

Objectives: To explore the association between adolescent multiple risk behaviours (MRB) and educational attainment.

Design: Prospective population-based UK birth cohort study.

Setting: Avon Longitudinal Study of Parents and Children (ALSPAC), a UK birth cohort of children born in 1991-92.

Participants: Data on some or all MRB measures were available for 5,401 ALSPAC participants who attended a clinic at age 15 years and/or completed a detailed questionnaire at age 16 years. Multiple imputation was used to account for missing data.

Primary outcome measures: Capped General Certificate of Secondary Education (GCSE) score and odds of attaining five or more GCSE examinations at grades A*-C. Both outcome measures come from the National Pupil Database (NPD) and were linked to the ALSPAC data.

Results: Engagement in MRB was strongly associated with poorer educational attainment. Each additional risk equated to -6.31 (95% CI -7.03, -5.58, p<0.001) in capped GCSE score, equivalent to a one grade reduction, or reduced odds of attaining five or more A*-C grades of 23% (OR 0·77, 95% CI: 0·74-0·81, p<0.001). The average cohort member engaged in 3·24 MRB and therefore have an associated reduction in GCSE score equivalent to three and a half grades in one examination, or reduced odds of attaining five or more A*-C grades of 75%.

Conclusion: Engagement in adolescent MRB is strongly associated with poorer educational attainment at 16 years. Preventing MRB could improve educational attainment and thereby directly and indirectly improve longer term health.

Keywords: multiple risk behaviours, adolescence, educational attainment, ALSPAC, UK birth cohort study

For peer review only - http://bmjopen.bmj.com/site/about/guidelines.xhtml

Strengths and limitations of this study

- This is the first longitudinal study in the UK to examine the association between multiple health risk behaviours and educational attainment.
- Owing to the scale and richness of the ALSPAC data, we have been able to control for a wide range of confounders, including socio-economic measures, previous educational attainment, and prior ability.
- The risk behaviours were all reduced to binary variables in order to construct the total MRB score, which leads to a loss of information.
- By summing the risk behaviours to create a measure of total MRBs, we attribute equal weight to each behaviour, however, we have found no strong evidence for an alternative classification of MRB in terms of clusters of specific MRBs or latent classes.
- There is considerable missing data for the exposure and confounder variables which reduces power and may introduce bias, however, there is no missing data on either outcome measure, and although missingness is related to MRB our imputed analyses are similar to the complete case analysis.

Sources of funding

ALSPAC receives core support from the UK Medical Research Council and the Wellcome Trust (grant reference 092731) and the University of Bristol. This work was undertaken with the support of The Centre for the Development and Evaluation of Complex Interventions for Public Health Improvement (DECIPHer), a UKCRC Public Health Research Centre of Excellence. Joint funding (MR/KO232331/1) from the British Heart Foundation, Cancer Research UK, Economic and Social Research Council, Medical Research Council, the Welsh Government and the Wellcome Trust, under the auspices of the UK Clinical Research Collaboration, is gratefully acknowledged.

For peer review only - http://bmjopen.bmj.com/site/about/guidelines.xhtml

Competing interests

The authors declare that there are no competing interests.

Introduction

Health risk behaviours such as smoking, alcohol consumption, physical inactivity, and unhealthy eating are prevalent during adolescence [1,2] and have also been shown to co-occur during this period. [3-5] A growing body of evidence suggests that these behaviours are strongly associated, some causally, with adverse health outcomes in later life, including chronic health conditions, morbidity, and premature mortality. [5-7] Further, evidence has shown that MRB are cumulatively associated with cardiovascular and all-cause mortality.[5,6] For example, having four healthy lifestyle behaviours is associated with a 4-fold difference in mortality compared to having none.[6] Similarly, a composite measure of MRB predicted preventable death, over and above the predictive value of single lifestyle behaviours.[8] It has also been posited that many lifestyle behaviours are underpinned by the same neural circuitry, and as such when intervening on one behaviour, changes in other behaviours can be expected.[9] Finally, interventions responding to multiple risks may be more efficient and better value for money, providing potentially huge benefits for considering multiple, rather than single risk behaviours.

Successful completion of compulsory education is important to an individual's well-being and lifelong opportunities. Those with lower educational attainment are more likely to smoke,[10] be overweight, and have poor physical and mental health outcomes.[11] They also experience reduced employment opportunities and earning potential,[11-13] while successful completion of compulsory education is strongly associated with increased aspirations and life satisfaction[14] and those with college degrees or higher are the most likely to engage in healthy behaviours.[15]

For peer review only - http://bmjopen.bmj.com/site/about/guidelines.xhtml

Many studies consider the effects of single health risk behaviours and educational outcomes: obesity,[3,16] smoking tobacco,[4,17,18] using cannabis,[17-19] drinking alcohol,[4,18] self-harm,[20] physical inactivity,[21] and screen based behaviours involving TV, internet, or computer games[22] are all associated with poorer educational outcomes. Less studies consider multiple risk behaviours simultaneously,[1,23-25] fewer consider a large number of heterogeneous risk behaviours concurrently.[26-28] It has been shown that those with no or intermediate qualifications are more likely to engage in MRB compared to those who attended higher education[15,29-31]. However, these studies relate to adult populations and have considered only a limited number or range of risk behaviours. However, no UK studies have examined engagement in multiple risk behaviours and educational outcomes. Using data from the ALSPAC cohort in England, we aimed to investigate the association of multiple risk behaviours during adolescence and educational attainment at age 16.

Methods

Sample

Data were drawn from the Avon Longitudinal Study of Parents and Children (ALSPAC), an ongoing prospective observational population-based study investigating the effects of a wide range of influences on health and development across the life course. Pregnant women residing in the old administrative county of Avon, who had an estimated date of delivery between 1 April 1991 and 31 December 1992, were invited to participate. The initial study cohort consisted of 14,062 live born children of whom 13,988 singletons or twins were still alive at 12 months of age. A small number of participants withdrew from the study (n=24).[32] Those who were neither enrolled nor part of the original core ALSPAC sample were excluded from our analyses, along with any triplets or quadruplets whose identity, because of their rarity, would be compromised. As Boyd et al. (2013) observe the ALSPAC 'enrolled sample' are more likely to be White (OR= 3.85 [95%CI: 3.50–4.24] p<0.001) and less likely

For peer review only - http://bmjopen.bmj.com/site/about/guidelines.xhtml

to be eligible for free school meals (OR= 0.46 [95%CI: 0.43–0.50] p<0.001) than the NPD KS4 GME national sample[2] and 'recent responders' to ALSPAC are more likely to be female (OR= 1.88 [95%CI: 1.74–2.03] p<0.001), White (OR=1.34 [95%CI: 1.10–1.62 p=0.004) and less likely to be eligible for free school meals (OR= 0.51 [95%CI: 0.44–0.60] p<0.001).[32] The study website contains details of all the data that are available through a fully searchable data dictionary: http://www.bris.ac.uk/alspac/researchers/data-access/data-dictionary/.[33] Ethical approval for the study was obtained from the ALSPAC Ethics and Law Committee and local Research Ethics Committees.

Linkage between ALSPAC and National Pupil Database

The National Pupil Database (NPD) is a pupil level data source which matches pupil and school characteristic data to pupil level attainment data in England. The Fischer Trust completed the linkage between the NPD and ALSPAC data in 2002. It is only mandatory for schools following the National Curriculum to contribute to the NPD. Independent schools may provide attainment data on a voluntary basis.

Exposure measure

Multiple risk behaviours at age 16

Measures of participation in thirteen distinct risk behaviours at the ages of 15 and 16 years were derived from participants' responses at two ALSPAC data collections during their late teens (see Table 1). The first was a self-completed questionnaire issued during a clinic attended at age 15 (median age 15 years and 5 months) and the second comprised responses to a postal questionnaire administered at age 16 (median age 16 years and 7 months). These behaviours represent a breadth of domains of social and health risk including: sexual health, substance use, self-harm, vehicle related injury risk, criminal and antisocial behaviour (ASB), and physical inactivity. The derivation of each behaviour is discussed in more detail in an

[2] Refers to all pupils, excluding those in ALSPAC, from English government-maintained establishments (GMEs) who sat their KS4 assessments during the same academic years as the ALSPAC cohort (academic years 2007–09).

For peer review only - http://bmjopen.bmj.com/site/about/guidelines.xhtml

earlier paper.[23] A previous analysis of these MRB data, using latent class analysis (LCA)[34], found that the model with the best fit showed that the resulting classes simply varied according to the number of risk behaviours, rather than demonstrating distinct risk profiles based on classes of behaviours. Therefore, there is no justification for the inclusion of analyses based on classes of behaviour and an MRB measure need only comprise a count of the number of risk behaviours. For the purposes of the analyses reported here a total number of risk-behaviours score from 0 to 13 was derived for each participant.

Outcome measures

Educational attainment at age 16

Pupils in England aged between 14 and 16 complete compulsory schooling and take their GCSE (or equivalent) examinations at key stage four (KS4). At the time, the ALSPAC cohort were in school, UK law stated that pupils were to remain in compulsory education until the age of 16, so unlike A-Levels, which are taken 2 years later and are optional, GCSEs are one of very few occasions in a young person's life when their educational attainment is assessed along with most of their peers. Two outcomes relating to KS4 educational attainment were used in the analysis. Achieving five or more A*-C grades at GCSE was chosen because it is a minimum requirement for many post-16 education and training courses and as such represents an important threshold for young people to exceed. The second outcome takes the individual scores for each GCSE, which are calculated as A*=58 through to G=16 and ungraded U=0 (unlike in North America where grades range from A-F). This general attainment score is then capped by choosing a pupil's eight best grades. Referred to as the capped GCSE score it is seen as preferable to a total GCSE score because: (i) it represents the same measure that is used in the published value-added school league tables, which have become an important measure of the quality of education provision; and (ii) this measure is

For peer review only - http://bmjopen.bmj.com/site/about/guidelines.xhtml

considered fairer than the total (uncapped) score since it moderates the scores of pupils who score highly merely by taking more examinations.

Possible Confounders

We adjusted for a number of known confounders: sex, season of birth, parent's highest social class (professional, managerial and technical, skilled non-manual or manual, part or unskilled managerial employment), mother's highest educational level (degree, A-level, O-level/GCSE and less than O-level/GCSE), household income (divided into quintiles of high to low income), housing tenure during pregnancy (mortgage or own property, privately rented property or subsidised rental property) and claiming eligibility for a free school meal. Season of birth has been shown to be an important predictor of educational attainment. In England, where the academic year runs from September 1st to August 31st, this means that children who are born in the autumn tend to outperform those who are born in the summer.[35] We additionally controlled for IQ score at age eight years and key stage two educational attainment in order to reduce the likelihood of reverse causality between early educational performance and engagement in MRBs. Analysis of confounders and both the exposure and outcomes variables was conducted and can be found in the supplementary material.

Missing data

Of the starting sample of 13,954 subjects (enrolled cohort, singletons and twins alive at one year): 2,618 (18·8%) did not have a linked education record for KS4 and were excluded from the analysis on that basis. There are a number of reasons for this type of missingness: they may not have the linked data from the national pupil database (NPD), the participant may have withheld consent, or the participant may have been attending a school that does not follow the National Curriculum i.e. an independent school. Independent school education is of particular interest in this case because of its prominence in Bristol and Avon. Between 2006 and 2009 (when the ALSPAC cohort would have taken their GCSE examinations), the

For peer review only - http://bmjopen.bmj.com/site/about/guidelines.xhtml

percentage of pupils educated in independent schools in England remained stable at approximately 7%. In Bristol, it ranged between 15·1% in 2006/07 and 13·4% in 2008/09. However, with no way of confirming that those with missing attainment and school type data were independently educated, and no alternative identifier of independent school status, we were unable to conduct a sensitivity analysis with this respect. Our sample analysis is therefore less representative of Bristol at the time, but more generalizable to the overall population, where independent schooling is less common. Of the 11,336 subjects with education outcome data, 8,398 (74.08%) were invited to the TF3 clinic and of those, 4,534 (53.99%) attended and 8,017 (70.72%) were sent the questionnaire and of those, data was available for 4,052 (50.54%) and 5,401 had some or all of their MRB information and this became the imputation sample upon which our analysis is based. Additional analysis regarding those with complete data (n=1,617) is available in the supplementary material (see Figure 1 for how we derived the sample).

Multivariate imputation by chained equations was carried out using the 'ice' routine in Stata version 14. This approach is based on the Missing At Random (MAR) assumption, i.e. that any differences between the missing and observed values, can be explained by differences in the observed data[36]. All variables used in the analyses including all measures of MRB, educational attainment and potential confounders are included in the imputation model, along with many other measures of the exposures, outcomes and confounders that had been collected at different time points. These are included in the imputation routine as auxiliary variables to reduce bias by improving the precision of the imputation model. Monte Carlo errors were used to compare the results obtained when imputing 25, 100, 250, and 500 data sets [37]. Imputed results shown have been pooled across the 500 data sets. Among this sample there was an average missingness of 18.23%.

Patient and Public Involvement

For peer review only - http://bmjopen.bmj.com/site/about/guidelines.xhtml

No patients were involved in the design of this study. However, our choice of risk behaviours was informed by discussion with two groups of adolescents through the DECIPHer ALPHA young person's research advisory group (http://decipher.uk.net/public-involvement/young-people/).

Sample descriptive statistics

Compared to the imputation sample the complete case sample had better educational outcomes, a higher mean IQ score and a lower mean total MRB score. The percentage of females, and all indicators of advantageous socio-economic position were over-represented in the complete case sample when compared with the imputation sample (see Table 2).

Associations between confounders and exposure variables

We found that lower maternal education, lower parental social class, living in privately rented or subsidised housing, having a lower income, and claiming eligibility for free school meals (FSM) were all positively associated with the total number of multiple risk behaviours at age 16. IQ at age 8 and higher previous educational attainment at key stage two were negatively associated with total MRB score. There was no association between sex, season of birth, or special educational needs (SEN) and MRB score.

Lower maternal education, lower parental social class, living in privately rented or subsidised housing, having a lower income, and claiming eligibility for free school meals (FSM) were all associated with lower educational attainment at age 16. This was true for both educational outcomes. Being born in the spring or summer compared with the autumn was associated with lower capped GCSE score, but had no effect on gaining five or more A*-C grades at GCSE. Having higher previous attainment levels at key stage two and a higher IQ at age 8 were associated with better educational outcomes at age 16 and being female was associated with an improved grade point average equivalent to more than one and a half grades. Details of these analyses can be found in the supplementary material.

For peer review only - http://bmjopen.bmj.com/site/about/guidelines.xhtml

Statistical analysis

Linear regression was used to examine associations between total MRB and the continuous outcome, capped GCSE score and logistic regression was used for the binary outcome, gaining five or more GCSE grades A*-C. Analyses were conducted on the complete case and imputed data the latter of which constituted the main analysis. We ran unadjusted models for both outcomes followed by a sequence of models adjusted for: (i) sex and season of birth, (ii) sex, season of birth, maternal education, parental social class, household income, tenure and FSM, and (iii) sex, season of birth maternal education, parental social class, household income, tenure, FSM, IQ, and previous educational attainment. We tested for non-linearity between exposure and outcome using the 'nlcheck' function in Stata, however, found no evidence that the linearity assumption had been violated. We also tested for interactions between sex and MRB; and each of the socio-economic indicators and MRB, in terms of their effect on educational outcomes, but found no strong evidence of any associations (p values range from 0·047 for housing tenure to 0·827 for FSM). All analyses were conducted in Stata version 14.

Results

Associations between total MRB and educational outcomes

In the ALSPAC cohort at age 16 years, the total number of multiple risk behaviours engaged in was strongly associated with lower educational attainment (see Table 3). In the unadjusted models, for every additional risk behaviour a young person engaged in, the capped GCSE score decreased on average 9·17 points, equivalent to a grade and a half in one GCSE examination. Similarly, the odds of attaining five or more A*-C grades were reduced by 18% for each additional risk behaviour.

The association between multiple risk behaviours and capped GCSE score did not change markedly with the inclusion of sex and season of birth in the models. However, a more

For peer review only - http://bmjopen.bmj.com/site/about/guidelines.xhtml

substantial reduction in the association was seen with the inclusion of the socio-economic indicators (maternal education, parental social class, household income, housing tenure, and free school meal status). The final and fully adjusted model, which additionally included IQ at age 8 and previous educational attainment, shows that for each additional multiple risk behaviour, the participant will see a reduction in capped GCSE score of 6·31 points, which is equivalent to just more than one grade in one GCSE examination. The mean number of risk behaviours engaged in was 3·24, which means that on average young people from this cohort have a reduced GCSE score of 20·44 points, associated with their risk taking. This is equivalent to a reduction of nearly three and a half grades in one GCSE examination, or one grade lower in three examinations.

The negative association between engaging in MRBs and gaining five or more GCSEs between A*-C, as demonstrated in the preliminary analysis, remained large in the adjusted models. Indeed, after adjusting for all the confounders, the magnitude of this effect increased and the odds of attaining five or more A*-C grades was reduced by 23% for each additional risk behaviour engaged in, although it should be noted that the confidence intervals do overlap with the previous model.

There were contrasting patterns of association between the separate risk behaviours and educational attainment. Smoking had the largest deleterious association and those who smoked scored an average of 57·40 capped GCSE points less than those who did not smoke. This would be equivalent to getting an ungraded U classification instead of an A* in one GCSE examination, or put differently getting one grade lower in nine separate GCSE examinations. Smokers were also 70% less likely to gain five or more A*-C grades at GCSE compared to non-smokers at age 16. Not wearing a cycle helmet, hazardous alcohol use, and physical inactivity were all negatively associated with educational attainment, but the

For peer review only

For peer review only - http://bmjopen.bmj.com/site/about/guidelines.xhtml

evidence was less compelling (with p values ranging from 0.007 to 0.934). Details of these analyses can be found in the supplementary material.

Discussion

In our analysis of the ALSPAC cohort, adolescents with a greater number of health risk behaviours had poorer educational outcomes at age 16. While the fully adjusted models for both the complete case and the imputed datasets showed some attenuation in the estimates for MRB with respect capped GCSE score, the effect remained strong despite adjusting for a wide range of confounders. The fully adjusted model showed a reduction in the association between capped GCSE score of 6.31 points, which would be equivalent to a reduction of more than one grade in one GCSE examination, for each additional MRB engaged in. A similarly adverse association with a young person's odds of gaining five or more GCSEs between A*-C was also observed with the odds of attaining five or more A*-C grades at GCSE reduced by 23% for each additional risk behaviour. We also corroborated associations between a wide range of individual MRB and educational outcomes at age 16.

This is the first longitudinal study in the UK to examine the association between a multiple health risk behaviours and educational attainment. Current research in this area often considers single risk behaviours or small 'clusters' of risks, but none to our knowledge consider a large number, of heterogeneous risks simultaneously. Owing to the scale and richness of the ALSPAC data, we have been able to control for a wide range of confounders, including socio-economic measures, previous educational attainment, and prior ability.

However, there are several limitations to our analysis. Firstly, the risk behaviours were all reduced to binary variables in order to construct the total MRB score, which leads to a loss of information. It is possible that individual behaviours showing no relationship with educational attainment in this study would show associations if examined using a different classification of risk behaviour (e.g. hazardous alcohol use and physical inactivity), but we

For peer review only - http://bmjopen.bmj.com/site/about/guidelines.xhtml

think it unlikely to have an impact on relationship between number of MRB and outcomes. Secondly, by summing the risk behaviours to create a measure of total MRBs, we attribute equal weight to each behaviour, however, we have found no strong evidence for an alternative classification of MRB in terms of clusters of specific MRBs or latent classes. Thirdly, there is considerable missing data on confounders and MRB which reduces power and may introduce bias, for example there may be selection bias the 5,401 are more likely to have female gender and higher IQ; and less likely to be from the lowest income quintile; to be living in privately or subsidised rental property; to have ever claimed eligibility for free school meals; and come from the lower parental social class ($p<0.001$). However, there is no missing data on either outcome measure, and although missingness is related to MRB our imputed analyses are similar to the complete case analysis and we do not expect there to be substantial bias. Fourth, some of the MRB were assessed using questionnaires, which have potential for recall bias and social desirability bias. Finally, we may not have controlled for all relevant confounders, for example, lone parent status and child maltreatment which are both be associated with poorer educational outcomes were not included in the analysis. However, we have included sufficient alternative confounders (parental social class, maternal education, housing tenure and claiming eligibility for free school meals), that are all themselves strongly associated with lone parent status and childhood maltreatment and as such, their omission will have a negligible effect.

The findings from this research build on and are consistent with current studies which have shown positive associations between single risk behaviours or small numbers of similar risks and poorer educational attainment [3,4,16-22,38-40]. They also echo findings from a US study which found strong evidence of associations between higher educational attainment and membership to the most-healthy cluster of adolescents. Although that study did not show the same dose-response relationship that we have found [15].

For peer review only - http://bmjopen.bmj.com/site/about/guidelines.xhtml

Engagement in multiple health risk behaviours during adolescence is strongly associated with lower educational attainment at age 16 years. Although we have not shown a causal association between exposure and outcome measures, our findings demonstrate for the first time that multiple health risk behaviours act as an important predictor of adverse educational outcomes, over and above a wide range of confounders including intellectual ability (IQ) at age 8 years and previous educational attainment. This finding could aid the identification and targeting of young people at risk of under-achieving during their compulsory education. Further, by showing a dose-response relationship between the two, we have shown the importance of intervening in and reducing each and every risk behaviour. Preventing multiple risk behaviours during adolescence could improve educational attainment and thereby directly and indirectly improve longer term health outcomes.

This research has not been able to establish the direction of the association between MRB and educational attainment, which would provide a valuable focus for future work in this area. Future work with this cohort would enable investigation of repeated measures of MRB at different time points throughout childhood and adolescence, in order to explore if there is any difference in the association between MRB and educational outcomes according to timing of MRB. Further research is also required to identify the early-life antecedents that are associated with adolescent MRB, as this would facilitate effective early intervention of those with the highest risk of engaging in harmful MRB.

Contributorship Statement

CW, RK, MH, RC and JH conceived and designed the study. CW and JH carried out the study, including acquiring and analysing the data. CW and JH interpreted the data. CW drafted the manuscript. RK, MH, RC and JH critiqued the manuscript for important intellectual content. All authors have read and approved the final version of the manuscript. CW serves as guarantor. MH and RC are senior investigators for the National Institute for

For peer review only - http://bmjopen.bmj.com/site/about/guidelines.xhtml

Health Research. CW and JH had full access to all of the data and can take responsibility for the integrity of the data and the accuracy of the data analysis. CW, RK, MH, RC and JH had access to the statistical outputs, reports, and tables. All researchers are independent of the funding bodies.

Data sharing statement

The ALSPAC policy on data sharing is available at www.bristol.ac.uk/alspac. To discuss access to ALSPAC data, please contact the ALSPAC executive team on alspac-exec@bristol.ac.uk.

Acknowledgements

We are extremely grateful to all the families who took part in this study, the midwives for their help in recruiting them, and the whole ALSPAC team, which includes interviewers, computer and laboratory technicians, clerical workers, research scientists, volunteers, managers, receptionists and nurses. The UK Medical Research Council and the Wellcome Trust (Grant ref: 102215/2/13/2) and the University of Bristol provide core support for ALSPAC.

For peer review only

For peer review only - http://bmjopen.bmj.com/site/about/guidelines.xhtml

REFERENCES

1. de Winter AF, Leenke V, Verhulst FC, Vollebergh WAM, Reijneveld SA. Longitudinal patterns and predictors of multiple health risk behaviors among adolescents: The TRAILS study. *Prev Med* 2015; **84**: 76-82.
2. Mays D, Peshkin BN, Sharff ME, et al. Correlates of adherence to a telephone-based multiple health behavior change cancer preventive intervention for teens: the Healthy for Life Program (HELP). *Health Education and Behavior* 2012; **39**(1): 18-26.
3. Booth JN, Tomporowski PD, Boyle JME, et al. Obesity impairs academic attainment in adolescence: findings from ALSPAC, a UK cohort. *Int J Obes (Lond)* 2014; **38**(10): 1335-42.
4. Latvala A, Rose RJ, Pulkkinen L, Dick DM, Korhonen T, Kaprio J. Drinking, smoking, and educational achievement: cross-lagged associations from adolescence to adulthood. *Drug and Alcohol Dependence* 2014; **137**: 106-13.
5. McCullough ML, Patel AV, Kushi LH, et al. Following cancer prevention guidelines reduces risk of cancer, cardiovascular disease, and all-cause mortality. *Cancer Epidemiology, Biomarkers & Prevention* 2011; **20**(6): 1089-97.
6. Kvaavik E, Batty D, Ursin G, Huxley R, Gale C. Influence of Individual and Combined Health Behaviors on Total and Cause-Specific Mortality in Men and Women. *Archives of Internal Medicine* 2010; **170**(8): 711-8.
7. Connor J. Alcohol consumption as a cause of cancer. *Addiction* 2016; **112**(2): 222–8.
8. Tamakoshi A, Tamakoshi K, Lin Y, Yagyu K, Kikuchi S, Group JS. Healthy lifestyle and preventable death: findings from the Japan Collaborative Cohort (JACC) Study. *Prev Med* 2009; **48**(5): 486-92.
9. Spring B, Schneider K, McFadden HG, et al. Make Better Choices (MBC): Study design of a randomized controlled trial testing optimal technology-supported change in multiple diet and physical activity risk behaviors. *BMC Public Health* 2010; **10**(1): 586.
10. Gilman SE, Martin LT, Abrams DB, et al. Educational attainment and cigarette smoking: a causal association? *Int J Epidemiol* 2008; **37**(3): 615-24.
11. Marmot M. Fair society healthy lives full report. 2010.
12. Ward JL, Viner RM. Secondary Education and Health Outcomes in Young People from the Cape Area Panel Study (CAPS). *PLoS One* 2016; **11**(6): 1-14.
13. ONS. Full Report - Graduates in the UK Labour. 2013.
14. Education Df. Youth Cohort Study & Longitudinal study of young people in England: The Activities and Experiences of 19 year olds: England 2010. In: Education Df, editor.; 2011.
15. Skalamera J, Hummer RA. Educational attainment and the clustering of health-related behavior among U.S. young adults. *Prev Med* 2016; **84**: 83-9.
16. Caird J, Kavanagh J, Oliver K, et al. Childhood obesity and educational attainment a systematic review. London: EPPI-Centre, Social Science Research Unit, Institute of Education, University of London: Institute of Education, University of London, 2011.
17. Stiby AI, Hickman M, Munafo MR, Heron J, Yip VL, Macleod J. Adolescent cannabis and tobacco use and educational outcomes at age 16: birth cohort study. *Addiction* 2015; **110**(4): 658-68.
18. Silins E, Fergusson DM, Patton GC, et al. Adolescent substance use and educational attainment: An integrative data analysis comparing cannabis and alcohol from three Australasian cohorts. *Drug and Alcohol Dependence* 2015; **156**: 90-6.

For peer review only - http://bmjopen.bmj.com/site/about/guidelines.xhtml

19. Verweij KJ, Huizink AC, Agrawal A, Martin NG, Lynskey MT. Is the relationship between early-onset cannabis use and educational attainment causal or due to common liability? *Drug and Alcohol Dependence* 2013; **133**(2): 580-6.
20. Mars B, Heron J, Crane C, et al. Clinical and social outcomes of adolescent self harm: population based birth cohort study. *BMJ* 2014; **349**: 1-13.
21. Booth JN, Leary SD, Joinson C, et al. Associations between objectively measured physical activity and academic attainment in adolescents from a UK cohort. *British Journal of Sports Medicine* 2014; **48**(3): 265-70.
22. Corder K, Atkin AJ, Bamber DJ, et al. Revising on the run or studying on the sofa: prospective associations between physical activity, sedentary behaviour, and exam results in British adolescents. *International Journal of Behavioral Nutrition and Physical Activity* 2015; **12**: 106.
23. Kipping RR, Smith M, Heron J, Hickman M, Campbell R. Multiple risk behaviour in adolescence and socio-economic status: findings from a UK birth cohort. *European Journal of Public Health* 2015; **25**(1): 44-9.
24. Alzahrani SG, Watt RG, Sheiham A, Aresu M, Tsakos G. Patterns of clustering of six health-compromising behaviours in Saudi adolescents. *BMC Public Health* 2014; **14**: 1215.
25. de Looze M, Ter Bogt TFM, Raaijmakers QAW, Pickett W, Kuntsche E, Vollebergh WAM. Cross-national evidence for the clustering and psychosocial correlates of adolescent risk behaviours in 27 countries. *European Journal of Public Health* 2015; **25**(1): 50-6.
26. Busch V, Van Stel HF, Schrijvers AJP, de Leeuw JRJ. Clustering behaviours outcomes Dutch adolescents. *BMC Public Health* 2013; **13**: 1-11.
27. Noel H, Denny S, Farrant B, et al. Clustering of adolescent health concerns: a latent class analysis of school students in New Zealand. *Journal of Paediatrics and Child Health* 2013; **49**(11): 935-41.
28. van Nieuwenhuijzen M, Junger M, Velderman MK, et al. Clustering of health-compromising behavior and delinquency in adolescents and adults in the Dutch population. *Prev Med* 2009; **48**(6): 572-8.
29. Buck D, F. F. Clustering of unhealthy behaviours over time: Implications for policy and practice. . London: Kings Fund, 2012.
30. Lawder R, Harding O, Stockton D, et al. Is the Scottish population living dangerously? Prevalence of multiple risk factors: the Scottish Health Survey 2003. *BMC Public Health* 2010; **10**(1): 330.
31. Shankar A, McMunn A, Steptoe A. Health-Related Behaviors in Older Adults: Relationships with Socioeconomic Status. *American Journal of Preventive Medicine* 2010; **38**(1): 39-46.
32. Boyd A, Golding J, Macleod J, et al. Cohort Profile: the 'children of the 90s'-the index offspring of the Avon Longitudinal Study of Parents and Children. *Int J Epidemiol* 2013; **42**(1): 111-27.
33. ALSPAC. Avon Longitudinal Study of Parents and Children. ALSPAC: Data Dictionary. 2016.
34. Heron J, Campbell R, Hickman M, Kipping RR. Latent class analysis of multiple risk behaviour in adolescents in the ALSPAC cohort. *BMC Public Health* under review.
35. Crawford C, Dearden L, Greaves E. The drivers of month-of-birth differences in children's cognitive and non-cognitive skills. *Journal of the Royal Statistical Society Series A, (Statistics in Society)* 2014; **177**(4): 829-60.
36. Sterne J, White J, Carlin J, et al. Multiple imputation for missing data in epidemiological and clinical research: potential and pitfalls. *BMJ* 2009; **338**: b2393.
37. White IR, Royston P, Wood AM. Multiple imputation using chained equations: Issues and guidance for practice. *Stat Med* 2011; **30**(4): 377-99.

For peer review only - http://bmjopen.bmj.com/site/about/guidelines.xhtml

38. Grant JD, Scherrer JF, Lynskey MT, et al. Associations of alcohol, nicotine, cannabis, and drug use/dependence with educational attainment: evidence from cotwin-control analyses. *Alcohol Clin Exp Res* 2012; **36**(8): 1412-20.
39. Horwood LJ, Fergusson DM, Hayatbakhsh MR, et al. Cannabis use and educational achievement: findings from three Australasian cohort studies. *Drug Alcohol Depend* 2010; **110**(3): 247-53.
40. Staff J, Patrick ME, Loken E, Maggs J. Teenage Alcohol Use and Educational Attainment. *Journal of Studies on Alcohol and Drugs* 2008; **69**(6): 848-58.

For peer review only

For peer review only - http://bmjopen.bmj.com/site/about/guidelines.xhtml

Table 1: Multiple risk behaviours (MRB) and their derivation

**Health risk behaviour**	**Definition / how derived**
Physical inactivity	Young person (YP) has typically over the past year exercised <5 times per week.
TV viewing	YP spent 3 or more hours watching television on average per day across the week.
Car passenger risk	YP had been in a car passenger at least once in their lifetime where the driver (a) had consumed alcohol or (b) did not have a valid licence, or (c) the YP chose not to wear a seat belt last time travelled in a car, van, or taxi.
Cycle helmet use	If the YP reported that they had last ridden a bicycle within the previous four weeks and they had not worn a helmet on the most recent occasion.
Scooter risk	YP has driven a motorbike/ scooter off road, or without a licence on a public road at least once.
Criminal/Antisocial behaviour	YP reported that at least once in the past year they had undertaken at least one of the following 7 offences: carried a weapon; physically hurt someone on purpose; stolen something; sold illicit substances to another person; damaged property belonging to someone else either by using graffiti, setting fire to it or destroying or damaging it in another fashion; subjected someone to verbal or physical racial abuse; or been rude/rowdy in a public place.
Hazardous alcohol consumption	In the past year had scored eight or more on the Alcohol Use Disorders Identification Test (AUDIT) indicating hazardous alcohol consumption.
Regular tobacco smoking	Has ever smoked and is regularly smoking by currently smoking at least one cigarette per week.
Cannabis use	Those who reported using cannabis "sometimes but less often than once

For peer review only - http://bmjopen.bmj.com/site/about/guidelines.xhtml

	a week" or more regular use were classified as occasional users.
Illicit drug/solvent use	In the year since their 15th birthday, YP had either been a regular user (i.e. used five or more times) of one or more illicit drugs (excluding cannabis) including amphetamines, ecstasy, LSD, cocaine, ketamine or inhalants including aerosols, gas, solvents and poppers.
Self-harm	Young people who said they had purposely hurt themselves in some way in their lifetime.
Penetrative sex before age 16	YP reported having had penetrative sex in the preceding year and that they were under 16 at the time.
Unprotected sex	Penetrative sex without the use of contraception on the last occasion they had had sex in the past year

Sources of information:

Age 15 years clinic: criminal and antisocial behaviour, penetrative sex prior to age 16, and unprotected sex.

Age 16 years questionnaire: physical inactivity, TV viewing, car passenger risk, cycle helmet use, scooter risk, hazardous alcohol drinking, regular smoking, illicit drug/solvent use, and self-harm.

For peer review only

For peer review only - http://bmjopen.bmj.com/site/about/guidelines.xhtml

Table 2: Sample descriptive statistics

		Imputation sample	Complete case sample
		**5,401**	**1,617**
		**% (SE)**	**n (%)**
Educational Outcomes	n=5,401		
Capped GCSE score: Mean (SE)		350 (1·01)	376 (1·31)
Five or more A*-C grades at GCSE		74·4% (SE=0·6)	1420 (87·8%)
Four or less A*-C grades at GCSE		25·6% (SE=0·6)	197 (12·2%)
Exposure	n=2,264		
MRB total: Mean (SE)		3.24 (0·31)	3.01 (0·05)
Sex	n=5,401		
Female		55·5% (SE=0·7)	975 (60·3%)
Male		44·5% (SE=0·7)	642 (39·7%
Season of birth	n=5,401		
Autumn		33·9% (SE=0·6)	542 (33·5%)
Winter		14·8% (SE=0·5)	222 (13·7%)
Spring		23·4% (SE=0·6)	382 (23·6%)
Summer		27·9% (SE=0·6)	471 (29·1%)
Maternal education	n=5,214		
Degree		14·9% (SE=0·5)	311 (19·2%)
A level		25·9% (SE=0·6)	479 (29·6%)
O level		34·3% (SE=0·7)	596 (36·9%)
< O level		21·5% (SE=0·6)	231 (14·3%)
Parental socio-economic position	n=4,970		
Professional		14·1% (SE=0·5)	291 (18·0%)
Managerial and technical		41·2% (SE=0·7)	761 (47·1%)

For peer review only

Skilled non-man		23·6% (SE=0·6)	398 (24·6%)
Skilled man, part or unskilled		13·2% (SE=0·5)	167 (10·3%)
Housing tenure	n=5,227		
Mortgage/owned		84·7% (SE=0·5)	1.444 (89·3%)
Private rent		7·3% (SE=0·4)	88 (5·4%)
Subsidised rent		8·6% (SE=0·4)	85 (5·3%)
Income	n=4,809		
High		20.86% (SE=0·6)	364 (22·5%)
Mid high		21.96% (SE=0·6)	424 (26·2%)
Middle		21.34% (SE=0·6)	364 (22·5%)
Mid low		19.25% (SE=0·6)	292 (18·1%)
Low		16.58% (SE=0·5)	173 (10·7%)
Free School Meals (FSM)	n=5,401		
Ever FSM		7·7% (SE=0·4)	65 (4·0%)
Never FSM		92·3% (SE=0·4)	1,552 (96·0%)
Special Educational Needs (SEN)	n=5,075		
No SEN		84·7% (SE=0·4)	1,522 (94·1%)
School Action		6·0% (SE=0·4)	68 (4·2%)
School Action Plus		1·9% (SE=0·2)	18 (1·1%)
Statement of SEN		1·4% (SE=0·2)	9 (0·6%)
Previous educational attainment/ability			
IQ at age 8: Mean (SE)	n=4,370	105 (0·24)	109 (0·37)
KS2 educational attainment: Mean (SE)	n=4,753	830 (2·76)	901 (3·94)

For peer review only

For peer review only - http://bmjopen.bmj.com/site/about/guidelines.xhtml

Table 3: Associations between total MRB score and capped GCSE score and odds of gaining five or more GCSEs at grade A*-C

	Unadjusted (n=5,401)	Model 1 (n=5,401)	Model 2 (n=5,401)	Model 3 (n=5,401)
**Capped GCSE**	-9·17 (-10·25, -8·10) p<0·001	-9·12 (-10·19, -8·05) p<0·001	-6·90 (-7·86, -5·94) p<0·001	-6·31 (-7·03, -5·58) p<0·001
**5 A*-C**	0·82 (0·79-0·85) p<0·001	0·82 (0·79-0·85) p<0·001	0·84 (0·81-0·87) p<0·001	0·77 (0·74-0·81) p<0·001

Model 1: adjusted for sex and season of birth

Model 2: adjusted for sex, season of birth maternal education, parental social class, household income, tenure, and FSM.

Model 3: adjusted for sex, season of birth maternal education, parental social class, household income, tenure, FSM, IQ, and previous educational attainment.

For peer review only - http://bmjopen.bmj.com/site/about/guidelines.xhtml

Figure 1: Deriving the sample

For peer review only

For peer review only - http://bmjopen.bmj.com/site/about/guidelines.xhtml

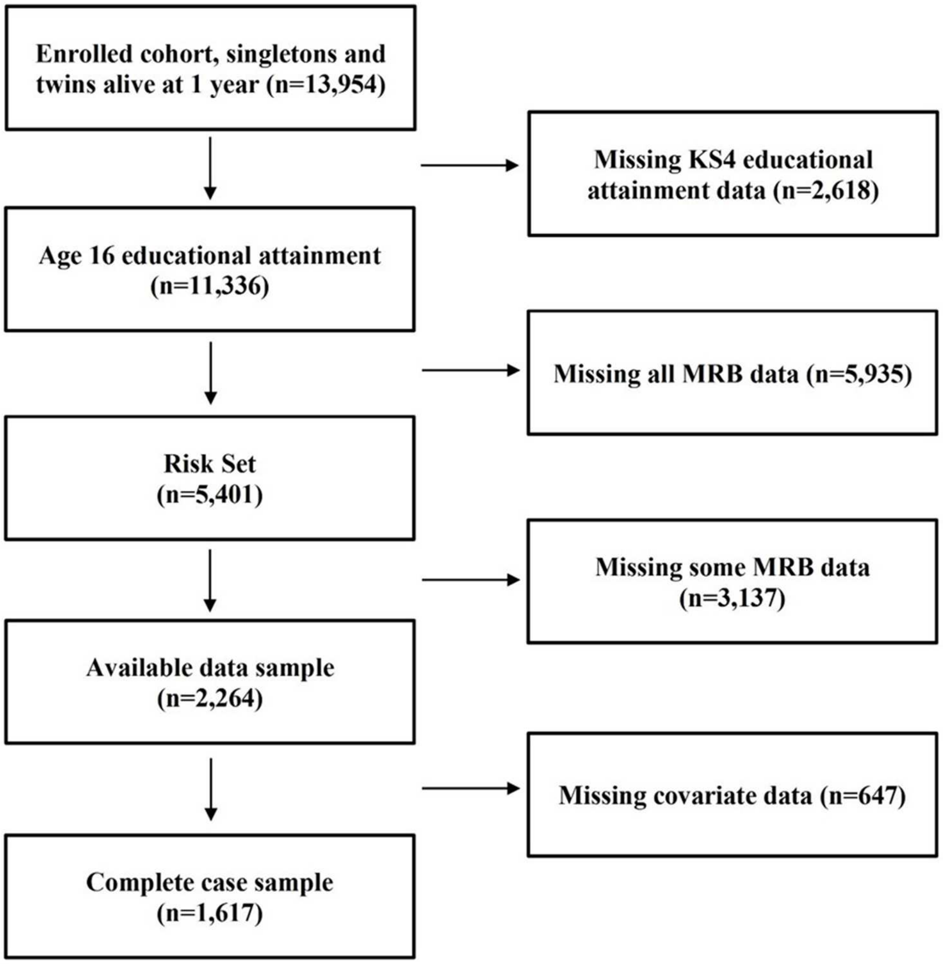

Figure 1: Deriving the sample

82x87mm (300 x 300 DPI)

Supplementary material table 1: Assessment for confounding, univariable associations with the exposure and outcome variables

	MRBs	Capped GCSE	Five or more GCSEs A*-C
**Gender ref: male**			
Female	-0.06 (-0.18, 0.06) p=0.343	10.83 (6.85, 14.81) p<0.001	1.32 (1.17, 1.49) p<0.001
**Season of birth ref: autumn**			
Winter	-0.04 (-0.23, 0.15) p=0.700	-4.18 (-10.35, 1.99) p=0.184	0.95 (0.79, 1.16) p=0.637
Spring	0.04 (-0.13, 0.20) p=0.674	-10.37 (-15.69, -5.05) p<0.001	0.78 (0.67, 0.92) p=0.004
Summer	0.26 (-0.13, 0.18) p=0.744	-13.34 (-18.40, -8.28) p<0.001	0.83 (0.71, 0.97) p=0.018
**Maternal education ref: degree**			
A level	0.17 (-0.02, 0.36) p=0.074	-35.19 (-41.13, -29.25) p<0.001	0.18 (0.12, 0.27) p<0.001
O level	0.41 (0.23, 0.59) p<0.001	-57.89 (-63.55, -52.23) p<0.001	0.09 (0.06, 0.14) p<0.001
< O level	0.49 (0.28, 0.69) p<0.001	-90.81 (-96.95, -84.68) p<0.001	0.04 (0.25, 0.06) p<0.001
**Parental socio-economic position ref: professional**			
Managerial and technical	0.43 (0.25, 0.61) p<0.001	-35.76 (-41.46, -30.05) p<0.001	0.29 (0.21, 0.39) p<0.001
Skilled non-man	0.47 (0.28, 0.67) p<0.001	-58.39 (-64.63, -52.15) p<0.001	0.15 (0.11, 0.21) p<0.001
Skilled man, part or unskilled	0.83 (0.60, 1.05) p<0.001	-88.71 (-95.76, -81.64) p<0.001	0.07 (0.05, 0.09) p<0.001

For peer review only

**Housing tenure ref: mortgage/owned**			
Private rent	0 .38 (0.14, 0.61) p=0.002	-20.07 (-27.50, -12.64) p<0.001	0.59 (0.47, 0.74) p<0.001
Subsidised rent	0 .75 (0.53, 0.97) p<0.001	-71.86 (-78.71, -65.01) p<0.001	0.18 (0.15, 0.74) p<0.001
**Income ref: high**			
Mid high	0.05 (-0.14, 0.24) p=0.615	-12.17 (-18.09, -6.26) p<0.001	0.62 (0.47, 0.80) p<0.001
Middle	0.17 (-0.02, 0.36) p=0.084	-32.02 (-37.98, -26.07) p<0.001	0.34 (0.26, 0.43) p<0.001
Mid low	0.35 (0.15, 0.55) p<0.001	-45.62 (-51.78, -39.46) p<0.001	0.23 (0.18, 0.29) p<0.001
Low	0.43 (0.23, 0.64) p<0.001	-74.31 (-80.75, -67.87) p<0.001	0.13 (0.10, 0.16) p<0.001
**FSM ref: never FSM**			
Ever FSM	0.97 (0.74, 1.21) p<0.001	-68.52 (-75.74, -61.30) p<0.001	0.22 (0.18, 0.27) p<0.001
**IQ at age 8**	-.0009 (-.0010, -.0006) p=0.009	2.69 (2.58, 2.79) p<0.001	1.09 (1.09, 1.10) p<0.001
**KS2 educational attainment**	-0.01 (-0.01, -0.01) p<0.001	0.26 (0.259, 0 .278) p<0.001	1.01 (1.01, 1.01) p<0.001
**SEN ref: no SEN**			
School Action	0.76 (-0.18, 0.33) p=0.555	-64.63 (-73.46, -55.80) p<0.001	0.19 (0.15, 0.25) p<0.001
School Action Plus	0.30 (-0.16, 0.77) p=0.201	-82.35 (-97.93, -66.78) p<0.001	0.13 (0.09, 0.21) p<0.001
Statement of SEN	-0.39 (-0.90, 0.12) p=0.134	-133.76 (-152.96, -114.56) p<0.001	0.11 (0.06, 0.19) p<0.001

For peer review only

Supplementary material table 2: Associations between total MRB score and educational outcomes, capped GCSE score and gaining five or more GCSEs at grade A*-C (complete case analysis)

	Complete Case Unadjusted (n=1,617)	Complete Case Adjusted[1] (n=1,617)	Complete Case Adjusted[2] (n=1,617)	Complete Case Adjusted[3] (n=1,617)
**Capped GCSE**	-6.12 (-7.42, -4.83) p<0.001	-6.13 (-7.42, -4.84) p<0.001	-4.82 (-6.00, -3.64) p<0.001	-4.48 (-5.31, -3.64) p<0.001
**5 A*-C**	0.81 (0.76–0.87) p<0.001	0.81 (0.75–0.87) p<0.001	0.84 (0.77-0.90) p<0.001	0.75 (0.68–0.83) p<0.001

[1] Adjusted for sex and season of birth.
[2] Adjusted for sex, season of birth maternal education, parental social class, household income, tenure and FSM.
[3] Adjusted for sex, season of birth maternal education, parental social class, household income, tenure FSM, IQ at age 8 and previous educational attainment at key stage 2.

For peer review only

Supplementary material table 3: Associations between separate MRB and educational outcomes, capped GCSE score and gaining five or more GCSEs at grade A*-C

	Capped GCSE score	Five or more GCSEs A*-C
**Car passenger risk**		
	-19.90 (-25.13, -14.67)	0.68 (0.58, 0.80)
	P<0.001	P<0.001
**Moped risk**	-35.18 (-41.51, -28.85)	0.42 (0.35, 0.51)
	P<0.001	P<0.001
**Cycle helmet risk**	-5.41 (-10.93, 0.12)	0.79 (0.68, 0.94)
	P=0.055	P=0.007
**Drug/solvent use**	-23.65 (-36.29, -10.99)	0.81 (0.57, 1.14)
	P<0.001	p=0.225
**Cannabis use**	-20.59 (-28.76, -12.42)	0.71 (0.56, 0.89)
	P<0.001	P=0.003
**Tobacco smoking**	-57.40 (-63.86, -50.94)	0.30 (0.25, 0.36)
	P<0.001	P<0.001
**Hazardous alcohol use**	-5.56 (-10.69, -0.43)	0.91 (0.77, 1.06)
	P=0.034	P=0.228
**Combined sexual risk**	-28.57 (-34.42, -22.72)	0.55 (0.47, 0.65)
	P<0.001	P<0.001
**Self-harm**	-12.24 (-18.17, -6.31)	0.83 (0.70, 0.99)
	P<0.001	P=0.041

For peer review only

**Criminal/Antisocial behaviour**	-22.76 (-27.2, -18.32)	0.58 (0.50, 0.66)
	P<0.001	P<0.001
**Physical inactivity**	-0.24 (-5.80, 5.33)	1.10 (0.92, 1.31)
	P=0.934	P=0.284
**TV viewing**	-23.97 (-29.82, -18.11)	0.60 (0.50, 0.72)
	P<0.001	P<0.001

For peer review only

STROBE Statement—Checklist of items that should be included in reports of ***cohort studies***

	Item No	Recommendation
**Title and abstract**	1	(*a*) Indicate the study's design with a commonly used term in the title or the abstract
**Pages 1 &2**		(*b*) Provide in the abstract an informative and balanced summary of what was done and what was found
**Introduction**		
Background/rationale **Page 4**	2	Explain the scientific background and rationale for the investigation being reported
Objectives **Page 4**	3	State specific objectives, including any prespecified hypotheses
**Methods**		
Study design **Page 5**	4	Present key elements of study design early in the paper
Setting **Page 5**	5	Describe the setting, locations, and relevant dates, including periods of recruitment, exposure, follow-up, and data collection
Participants	6	(*a*) Give the eligibility criteria, and the sources and methods of selection of participants. Describe methods of follow-up
**Page 4**		(*b*) For matched studies, give matching criteria and number of exposed and unexposed
Variables **Page 6&7**	7	Clearly define all outcomes, exposures, predictors, potential confounders, and effect modifiers. Give diagnostic criteria, if applicable
Data sources/ measurement **Pages 5-7**	8*	For each variable of interest, give sources of data and details of methods of assessment (measurement). Describe comparability of assessment methods if there is more than one group
Bias **Pages 7&8**	9	Describe any efforts to address potential sources of bias
Study size **Page 7&8**	10	Explain how the study size was arrived at
Quantitative variables **Pages 6-8**	11	Explain how quantitative variables were handled in the analyses. If applicable, describe which groupings were chosen and why
Statistical methods	12	(*a*) Describe all statistical methods, including those used to control for confounding
		(*b*) Describe any methods used to examine subgroups and interactions
**Pages 5-9**		(*c*) Explain how missing data were addressed
		(*d*) If applicable, explain how loss to follow-up was addressed
		(*e*) Describe any sensitivity analyses
**Results**		
Participants	13*	(a) Report numbers of individuals at each stage of study—eg numbers potentially eligible, examined for eligibility, confirmed eligible, included in the study, completing follow-up, and analysed
**Pages 7&8**		(b) Give reasons for non-participation at each stage
		(c) Consider use of a flow diagram
Descriptive data	14*	(a) Give characteristics of study participants (eg demographic, clinical, social) and information on exposures and potential confounders
**Page 10**		(b) Indicate number of participants with missing data for each variable of interest
		(c) Summarise follow-up time (eg, average and total amount)
Outcome data **Pages 5, 7**	15*	Report numbers of outcome events or summary measures over time

For peer review only

Main results<br><br>**Pages 12-14**	16	(*a*) Give unadjusted estimates and, if applicable, confounder-adjusted estimates and their precision (eg, 95% confidence interval). Make clear which confounders were adjusted for and why they were included
		(*b*) Report category boundaries when continuous variables were categorized
		(*c*) If relevant, consider translating estimates of relative risk into absolute risk for a meaningful time period
Other analyses<br>**Pages 9**	17	Report other analyses done—eg analyses of subgroups and interactions, and sensitivity analyses
**Discussion**		
Key results<br>**Page 10&11**	18	Summarise key results with reference to study objectives
Limitations<br>**Pages 3 & 12**	19	Discuss limitations of the study, taking into account sources of potential bias or imprecision. Discuss both direction and magnitude of any potential bias
Interpretation<br>**Pages 11&12**	20	Give a cautious overall interpretation of results considering objectives, limitations, multiplicity of analyses, results from similar studies, and other relevant evidence
Generalisability<br>**Page 8**	21	Discuss the generalisability (external validity) of the study results
**Other information**		
Funding<br>**Page 3**	22	Give the source of funding and the role of the funders for the present study and, if applicable, for the original study on which the present article is based

*Give information separately for exposed and unexposed groups.

Note: An Explanation and Elaboration article discusses each checklist item and gives methodological background and published examples of transparent reporting. The STROBE checklist is best used in conjunction with this article (freely available on the Web sites of PLoS Medicine at http://www.plosmedicine.org/, Annals of Internal Medicine at http://www.annals.org/, and Epidemiology at http://www.epidem.com/). Information on the STROBE Initiative is available at http://www.strobe-statement.org.

For peer review only

For peer review only - http://bmjopen.bmj.com/site/about/guidelines.xhtml

BMJ Open

The effect of multiple risk behaviours in adolescence on educational attainment at age 16 years: a UK birth cohort study

Journal:	*BMJ Open*
Manuscript ID	bmjopen-2017-020182.R2
Article Type:	Research
Date Submitted by the Author:	18-May-2018
Complete List of Authors:	Wright, Caroline; University of Bristol, Bristol Medical School<br>Kipping, Ruth; University of Bristol, Bristol Medical School<br>Hickman, M; University of Bristol, Bristol Medical School<br>Campbell, Rona; University of Bristol, Bristol Medical School<br>Heron, Jon; University of Bristol, Bristol Medical School
**Primary Subject Heading**:	Epidemiology
Secondary Subject Heading:	Public health
Keywords:	multiple risk behaviours, adolescence, educational attainment, ALSPAC, UK birth cohort study

SCHOLARONE™
Manuscripts

The effect of multiple risk behaviours in adolescence on educational attainment at age 16 years: a UK birth cohort study

Caroline Wright,[1] Ruth Kipping,[1] Matthew Hickman,[1] Rona Campbell,[1] and Jon Heron.[1]

Correspondence to: Caroline Wright, Population Health Sciences, Bristol Medical School, University of Bristol, BF4, Barley House, Oakfield Grove, Bristol, BS8 2BN

caroline.wright@bristol.ac.uk or +44 (0)117 3314011

For peer review only

[1] Population Health Sciences, Bristol Medical School, University of Bristol, Oakfield House, Bristol, BS82BN, UK.

For peer review only - http://bmjopen.bmj.com/site/about/guidelines.xhtml

Abstract

Objectives: To explore the association between adolescent multiple risk behaviours (MRB) and educational attainment.

Design: Prospective population-based UK birth cohort study.

Setting: Avon Longitudinal Study of Parents and Children (ALSPAC), a UK birth cohort of children born in 1991-92.

Participants: Data on some or all MRB measures were available for 5,401 ALSPAC participants who attended a clinic at age 15 years and/or completed a detailed questionnaire at age 16 years. Multiple imputation was used to account for missing data.

Primary outcome measures: Capped General Certificate of Secondary Education (GCSE) score and odds of attaining five or more GCSE examinations at grades A*-C. Both outcome measures come from the National Pupil Database (NPD) and were linked to the ALSPAC data.

Results: Engagement in MRB was strongly associated with poorer educational attainment. Each additional risk equated to -6.31 (95% CI -7.03, -5.58, p<0.001) in capped GCSE score, equivalent to a one grade reduction, or reduced odds of attaining five or more A*-C grades of 23% (OR 0·77, 95% CI: 0·74-0·81, p<0.001). The average cohort member engaged in 3·24 MRB and therefore have an associated reduction in GCSE score equivalent to three and a half grades in one examination, or reduced odds of attaining five or more A*-C grades of 75%.

Conclusion: Engagement in adolescent MRB is strongly associated with poorer educational attainment at 16 years. Preventing MRB could improve educational attainment and thereby directly and indirectly improve longer term health.

Keywords: multiple risk behaviours, adolescence, educational attainment, ALSPAC, UK birth cohort study

For peer review only - http://bmjopen.bmj.com/site/about/guidelines.xhtml

Strengths and limitations of this study

- This is the first longitudinal study in the UK to examine the association between multiple health risk behaviours and educational attainment.
- Owing to the scale and richness of the ALSPAC data, we have been able to control for a wide range of confounders, including socio-economic measures, previous educational attainment, and IQ.
- The risk behaviours were all reduced to binary variables in order to construct the total MRB score, which leads to a loss of information.
- By summing the risk behaviours to create a measure of total MRBs, we attribute equal weight to each behaviour, however, we found no strong evidence for an alternative classification of MRB in terms of clusters of specific MRBs or latent classes.
- There is considerable missing data for the exposure and confounder variables which reduces power and may introduce bias, however, there is no missing data on either outcome measure, and although missingness is related to MRB our imputed analyses are similar to the complete case analysis.

Sources of funding

ALSPAC receives core support from the UK Medical Research Council and the Wellcome Trust (grant reference 092731) and the University of Bristol. This work was undertaken with the support of The Centre for the Development and Evaluation of Complex Interventions for Public Health Improvement (DECIPHer), a UKCRC Public Health Research Centre of Excellence. Joint funding (MR/KO232331/1) from the British Heart Foundation, Cancer Research UK, Economic and Social Research Council, Medical Research Council, the Welsh Government and the Wellcome Trust, under the auspices of the UK Clinical Research Collaboration, is gratefully acknowledged.

Competing interests

For peer review only

For peer review only - http://bmjopen.bmj.com/site/about/guidelines.xhtml

The authors declare that there are no competing interests.

Introduction

Health risk behaviours such as smoking, alcohol consumption, physical inactivity, and unhealthy eating are prevalent during adolescence [1,2] and have also been shown to co-occur during this period. [3-5] A growing body of evidence suggests that these behaviours are strongly associated, some causally, with adverse health outcomes in later life, including chronic health conditions, morbidity, and premature mortality. [5-7] Further, evidence has shown that MRB are cumulatively associated with cardiovascular and all-cause mortality.[5,6] For example, having four healthy lifestyle behaviours is associated with a 4-fold difference in mortality compared to having none.[6] Similarly, a composite measure of MRB predicted preventable death, over and above the predictive value of single lifestyle behaviours.[8] It has been posited that many lifestyle behaviours are underpinned by the same neural circuitry, and as such when intervening on one behaviour, changes in other behaviours can be expected.[9] Finally, interventions responding to multiple risks may be more efficient and better value for money, providing potentially huge benefits for considering multiple, rather than single risk behaviours.

Successful completion of compulsory education is important to an individual's well-being and lifelong opportunities. Those with lower educational attainment are more likely to smoke,[10] be overweight, and have poor physical and mental health outcomes.[11] They also experience reduced employment opportunities and earning potential.[11-13] Successful completion of compulsory education is strongly associated with increased aspirations and life satisfaction[14] and those with college degrees or higher are the most likely to engage in healthy behaviours.[15]

Many studies consider the effects of single health risk behaviours and educational outcomes. Obesity,[3,16] smoking tobacco,[4,17,18] using cannabis,[17-19] drinking alcohol,[4,18] self-harm,[20]

For peer review only - http://bmjopen.bmj.com/site/about/guidelines.xhtml

physical inactivity,[21] and screen based behaviours involving TV, internet, or computer games[22] are all associated with poorer educational outcomes. Less studies consider multiple risk behaviours simultaneously,[1,23-25] fewer still consider a large number of heterogeneous risk behaviours concurrently.[26-28] Past research has shown that those with no or intermediate qualifications are more likely to engage in MRB compared to those who attended higher education. [15,29-31] However, these studies relate to adult populations and have considered only a limited number or range of risk behaviours. No UK studies, to our knowledge, have examined engagement in multiple risk behaviours and educational outcomes. Using data from the ALSPAC cohort in England, we aimed to investigate the association of multiple risk behaviours during adolescence and educational attainment at age 16.

Methods

Sample

Data were drawn from the Avon Longitudinal Study of Parents and Children (ALSPAC), an ongoing prospective observational population-based study investigating the effects of a wide range of influences on health and development across the life course. Pregnant women residing in the old administrative county of Avon, who had an estimated date of delivery between 1 April 1991 and 31 December 1992, were invited to participate. The initial study cohort consisted of 14,062 live born children of whom 13,988 singletons or twins were still alive at 12 months of age. A small number of participants withdrew from the study (n=24).[32] Those who were neither enrolled nor part of the original core ALSPAC sample were excluded from our analyses, along with any triplets or quadruplets whose identity, because of their rarity, would be compromised. As Boyd et al. (2013) observe the ALSPAC 'enrolled sample' are more likely to be White (OR= 3.85 [95%CI: 3.50–4.24] p<0.001) and less likely to be eligible for free school meals (OR= 0.46 [95%CI: 0.43–0.50] p<0.001) than the National Pupil Database key stage four government-maintained establishments national

For peer review only - http://bmjopen.bmj.com/site/about/guidelines.xhtml

sample[2] and 'recent responders' to ALSPAC are more likely to be female (OR= 1.88 [95%CI: 1.74–2.03] p<0.001), White (OR=1.34 [95%CI: 1.10–1.62 p=0.004) and less likely to be eligible for free school meals (OR= 0.51 [95%CI: 0.44–0.60] p<0.001).[32] The study website contains details of all the data that are available through a fully searchable data dictionary: http://www.bris.ac.uk/alspac/researchers/data-access/data-dictionary/.[33] Ethical approval for the study was obtained from the ALSPAC Ethics and Law Committee and local Research Ethics Committees.

Linkage between ALSPAC and National Pupil Database

The National Pupil Database (NPD) is a pupil level data source which matches pupil and school characteristic data to pupil level attainment data in England. The Fischer Trust completed the linkage between the NPD and ALSPAC data in 2002. It is only mandatory for schools following the National Curriculum to contribute to the NPD. Independent schools may provide attainment data on a voluntary basis.

Exposure measure

Multiple risk behaviours at age 16

Measures of participation in thirteen distinct risk behaviours at the ages of 15 and 16 years were derived from participants' responses at two ALSPAC data collections during their late teens (see Table 1). The first was a self-completed questionnaire issued during a clinic attended at age 15 (median age 15 years and 5 months) and the second comprised responses to a postal questionnaire administered at age 16 (median age 16 years and 7 months). The MRB measure was informed by the work of Hurrelmann and Richter (2006), who present an integrative model of risk behaviour. They argue that while inadequate coping processes are ubiquitous during adolescence, these processes can result in very different health risk behaviours among young people. However, despite variations in presentation (e.g. physically

[2] Refers to all pupils, excluding those in ALSPAC, from English government-maintained establishments (GMEs) who sat their KS4 assessments during the same academic years as the ALSPAC cohort (academic years 2007–09).

For peer review only - http://bmjopen.bmj.com/site/about/guidelines.xhtml

hurting someone on purpose, verses not wearing a seat belt), risk behaviours reflect the very similar dimensions of either externalising; internalising; or evasive forms. [34] Lending weight to this position, previous analyses using this measure of MRB have shown that health risk behaviours are patterned according to gender. For example, antisocial and criminal behaviours, cannabis use and vehicle-related risk behaviours are more prevalent among males, whilst tobacco smoking, self-harm and physical inactivity are more prevalent among females. However, despite the gendered patterning of single risk behaviours, females and males engaged in a similar number of risk behaviours. [35] Similarly, another previous analysis showed that while the associations between individual risk behaviours and measures of socioeconomic status (parental social class, maternal education and income quintile), were highly variable, a more consistent relationship was established between the MRB measure and socioeconomic status. When compared with the highest social class, maternal education or income quintile, the odds of engaging in a greater number of multiple risk behaviours increased for each incremental decrease in social position. [23] Finally, an analysis of these MRB data, using latent class analysis (LCA),[36] showed that the resulting classes simply varied according to the number of risk behaviours, rather than demonstrating distinct risk profiles based on classes of behaviours. Having found no strong evidence for employing an alternative classification of MRB based on classes of behaviour the MRB measure comprises a count of the number of risk behaviours representing a breadth of domains of social and health risk including: sexual health, substance use, self-harm, vehicle related injury risk, criminal and antisocial behaviour (ASB), and physical inactivity. The derivation of each behaviour is discussed in more detail in an earlier paper. [23] For the purposes of the analyses reported here a total number of risk-behaviours score from 0 to 13 was derived for each participant.

Outcome measures

For peer review only - http://bmjopen.bmj.com/site/about/guidelines.xhtml

Educational attainment at age 16

Pupils in England aged between 14 and 16 years complete compulsory schooling during school years 10 and 11 and take their GCSE (or equivalent) examinations, this is referred to as key stage four (KS4). At the time that the ALSPAC cohort were in school, UK law stated that pupils were to remain in compulsory education until the age of 16, so unlike A-Levels, which are taken 2 years later and are optional, GCSEs are one of very few occasions in a young person's life when their educational attainment is assessed along with most of their peers. Two outcomes relating to key stage four educational attainment were used in the analysis. Achieving five or more A*-C grades at GCSE was chosen because it is a minimum requirement for many post-16 education and training courses and as such represents an important threshold for young people to exceed. The second outcome takes the individual scores for each GCSE, which are calculated as A*=58 through to G=16 and ungraded U=0 (unlike in North America where grades range from A-F). This general attainment score is calculated by summing a pupil's eight best grades, referred to as the capped GCSE score. It is seen as preferable to a total GCSE score because it represents the same measure that is used in the published value-added school league tables, which have become an important measure of the quality of education provision. It is also considered fairer than the total (uncapped) score since it moderates the scores of pupils who score highly merely by taking more examinations.

Possible Confounders

We adjusted for a number of known confounders: sex, season of birth, parent's highest social class (professional; managerial and technical; skilled non-manual; and skilled manual, part or unskilled manual), mother's highest educational level (degree, A-level, O-level/GCSE and less than O-level/GCSE), household income (divided into quintiles of high to low income), housing tenure during pregnancy (mortgaged or own property, privately rented property or

For peer review only - http://bmjopen.bmj.com/site/about/guidelines.xhtml

subsidised rental property) and claiming eligibility for a free school meal. Season of birth has been shown to be an important predictor of educational attainment. In England, where the academic year runs from September 1st to August 31st, children who are born in the autumn tend to outperform those who are born in the summer.[37] We additionally controlled for IQ score at age eight years and key stage two[3] educational attainment in order to reduce the likelihood of reverse causality between early educational performance and engagement in MRBs. Analysis of confounders and both the exposure and outcomes variables was conducted and can be found in the supplementary material.

Missing data

Of the starting sample of 13,954 subjects (enrolled cohort, singletons and twins alive at one year): 2,618 (18·8%) did not have a linked education record for key stage four and were excluded from the analysis on that basis. There are a number of possible reasons for this type of missingness. Participants' may not have the linked data from the national pupil database (NPD), the participant may have withheld consent, or the participant may have been attending a school that does not follow the National Curriculum i.e. an independent school. Independent school education is of particular interest in this case because of its prominence in Bristol and Avon. Between 2006 and 2009 (when the ALSPAC cohort would have taken their GCSE examinations), the percentage of pupils educated in independent schools in England remained stable at approximately 7%. In Bristol, it ranged between 15·1% in 2006/07 and 13·4% in 2008/09. However, with no way of confirming that those with missing attainment and school type data were independently educated, and no alternative identifier of independent school status, we were unable to conduct a sensitivity analysis with this respect. Our sample analysis is therefore less representative of Bristol at the time, but more

[3] Key stage two is the legal term which refers to the four years of schooling in maintained schools in England and Wales when pupils are aged between 7 and 11 years. Key stage two culminates in standardized assessment tests (SATS) at age ~11 years, the results of which have been used here.

For peer review only - http://bmjopen.bmj.com/site/about/guidelines.xhtml

generalizable to the overall population, where independent schooling is less common. Of the 11,336 subjects with education outcome data, 8,398 (74.08%) were invited to the clinic and of those, 4,534 (53.99%) attended; and 8,017 (70.72%) were sent the questionnaire and of those, data was available for 4,052 (50.54%). Overall, 5,401 participants had some or all of their MRB information and this became the imputation sample upon which our analysis is based. Additional analysis regarding those with complete data (n=1,617) is available in the supplementary material (see Figure 1 for how we derived the sample).

Multivariate imputation by chained equations was carried out using the 'ice' routine in Stata version 14. This approach is based on the Missing At Random (MAR) assumption, i.e. that any differences between the missing and observed values, can be explained by differences in the observed data.[38] All variables used in the analyses including all measures of MRB, educational attainment and potential confounders were included in the imputation model, along with many other measures of the exposures, outcomes and confounders that had been collected at different time points. These are included in the imputation routine as auxiliary variables to reduce bias by improving the precision of the imputation model. Monte Carlo errors were used to compare the results obtained when imputing 25, 100, 250, and 500 data sets. [39] Imputed results shown have been pooled across the 500 data sets. Among this sample there was an average missingness of 18.23%.

Patient and Public Involvement

No patients were involved in the design of this study. However, our choice of risk behaviours was informed by discussions with two groups of adolescents through the DECIPHer ALPHA young person's research advisory group (http://decipher.uk.net/public-involvement/young-people/).

Sample descriptive statistics

For peer review only - http://bmjopen.bmj.com/site/about/guidelines.xhtml

Compared to the imputation sample the complete case sample had better educational outcomes, a higher mean IQ score and a lower mean total MRB score. The percentage of females, and all indicators of advantageous socio-economic position were over-represented in the complete case sample when compared with the imputation sample (see Table 2).

Associations between confounders and exposure variables

We found that lower maternal education, lower parental social class, living in privately rented or subsidised housing, having a lower income, and claiming eligibility for free school meals (FSM) were all positively associated with the total number of multiple risk behaviours at age 16 years. IQ at age 8 years and higher previous educational attainment at key stage two were negatively associated with total MRB score. There was no association between sex, season of birth, or special educational needs (SEN) and MRB score.

Lower maternal education, lower parental social class, living in privately rented or subsidised housing, having a lower income, and claiming eligibility for free school meals (FSM) were all associated with lower educational attainment at age 16 years. This was true for both educational outcomes. Being born in the spring or summer compared with the autumn was associated with lower capped GCSE score, but had no association with gaining five or more A*-C grades at GCSE. Having higher previous attainment levels at key stage two and a higher IQ at age 8 years were associated with better educational outcomes at age 16 years and being female was associated with an improved grade point average equivalent to more than one and a half grades. Details of these analyses can be found in the supplementary material.

Statistical analysis

Linear regression was used to examine associations between total MRB and the continuous outcome, capped GCSE score and logistic regression was used for the binary outcome, gaining five or more GCSE grades A*-C. Analyses were conducted on the complete case and imputed data the latter of which constitutes the main analysis. We ran unadjusted models for

For peer review only - http://bmjopen.bmj.com/site/about/guidelines.xhtml

both outcomes followed by a sequence of models adjusted for: (i) sex and season of birth, (ii) sex, season of birth, maternal education, parental social class, household income, housing tenure and free school meals (FSM), and (iii) sex, season of birth, maternal education, parental social class, household income, housing tenure, FSM, IQ, and previous educational attainment. We tested for non-linearity between exposure and outcome using the 'nlcheck' function in Stata. However, we found no evidence that the linearity assumption had been violated. We also tested for interactions between sex and MRB; and each of the socio-economic indicators and MRB, however, we found no strong evidence of any associations (p values ranged from 0·047 for housing tenure to 0·827 for FSM). All analyses were conducted in Stata version 14.

Results

Associations between total MRB and educational outcomes

In the ALSPAC cohort at age 16 years, we found that the total number of multiple risk behaviours engaged in was strongly associated with lower educational attainment (see Table 3). In the unadjusted models, for every additional risk behaviour a young person engaged in, the capped GCSE score decreased on average 9·17 points (95% CI: -10·25, -8·10 p<0.001), equivalent to a grade and a half in one GCSE examination. Similarly, the odds of attaining five or more A*-C grades were reduced by 18% (OR=0·82 [95% CI: 0·79-0·85] p<0.001) for each additional risk behaviour.

The association between multiple risk behaviours and capped GCSE score did not change markedly with the inclusion of sex and season of birth in the models. However, a more substantial reduction in the association was seen with the inclusion of the socio-economic indicators (maternal education, parental social class, household income, housing tenure, and free school meal status). The final and fully adjusted model, which additionally included IQ at age 8 years and previous educational attainment, shows that for each additional multiple

For peer review only - http://bmjopen.bmj.com/site/about/guidelines.xhtml

risk behaviour, the participant had a reduction in capped GCSE score of 6·31 points (95% CI -7.03, -5.58, p<0.001) which is equivalent to just more than one grade in one GCSE examination. The mean number of risk behaviours engaged in was 3·24, which means that on average young people from this cohort had a reduced GCSE score of 20·44 points, associated with their risk taking. This is equivalent to a reduction of nearly three and a half grades in one GCSE examination, or one grade lower in three examinations.

The negative association between engaging in MRBs and gaining five or more GCSEs between A*-C, as demonstrated in the preliminary analysis, remained large in the adjusted models. Indeed, after adjusting for all the confounders, the magnitude of this effect increased and the odds of attaining five or more A*-C grades was reduced by 23% (OR = 0·77 [95% CI: 0·74-0·81] p<0.001) for each additional risk behaviour engaged in (although it should be noted that the confidence intervals do overlap with the previous model).

Each of the separate risk behaviours were negatively associated with educational attainment. Smoking had the largest deleterious association and those who smoked scored an average of 57·40 capped GCSE points less than those who did not smoke. This would be equivalent to getting an ungraded U classification instead of an A* in one GCSE examination, or put differently getting one grade lower in nine separate GCSE examinations. Smokers were also 70% less likely to gain five or more A*-C grades at GCSE compared to non-smokers at age 16 years. Not wearing a cycle helmet, hazardous alcohol use, and physical inactivity were all negatively associated with educational attainment, however, the evidence was less compelling (with p values ranging from 0.007 to 0.934). Details of these analyses can be found in the supplementary material.

Discussion

In our analysis of the ALSPAC cohort, adolescents with a greater number of health risk behaviours had poorer educational outcomes at age 16 years. While the fully adjusted models

For peer review only

For peer review only - http://bmjopen.bmj.com/site/about/guidelines.xhtml

for both the complete case and the imputed datasets showed some attenuation in the estimates, the effect remained strong despite adjusting for a wide range of confounders. The fully adjusted model showed an associated reduction in capped GCSE score of 6.31 points, which would be equivalent to a reduction of more than one grade in one GCSE examination, for each additional MRB engaged in. A similarly adverse association with a young person's odds of gaining five or more GCSEs between A*-C was also observed with the odds of attaining five or more A*-C grades at GCSE reduced by 23% for each additional risk behaviour. We also corroborated associations between a wide range of individual MRB and educational outcomes at age 16 years.

This is the first longitudinal study in the UK to examine the association between multiple health risk behaviours and educational attainment. Current research in this area often considers single risk behaviours or small 'clusters' of risks, but none to our knowledge consider a large number of heterogeneous risks simultaneously. Owing to the scale and richness of the ALSPAC data, we have been able to control for a wide range of confounders, including socio-economic measures, previous educational attainment, and IQ.

However, there are several limitations to our analysis. Firstly, the risk behaviours were all reduced to binary variables in order to construct the total MRB score, which leads to a loss of information. While each of the individual behaviours showed a negative association with educational attainment, these associations would perhaps be more robust if examined using a different classification of risk behaviour (e.g. hazardous alcohol use and physical inactivity). However, we think it unlikely to have an impact on the relationship between number of MRB and educational attainment. Secondly, by summing the risk behaviours to create a measure of total MRBs, we attribute equal weight to each behaviour, however, we have found no strong evidence for an alternative classification of MRB in terms of clusters of specific MRBs or latent classes. Thirdly, there is considerable missing data on confounders and MRB which

For peer review only - http://bmjopen.bmj.com/site/about/guidelines.xhtml

reduces power and may introduce bias. Whilst our outcome variables, obtained through linkage, were observed for the majority of the participants, we opted to restrict our analyses (and our imputation) to the 5,401 providing information on at least one risk behaviour. This subsample of ALSPAC were more likely to be female and to have higher IQ; and less likely to be from the lowest income quintile; to be living in privately or subsidised rental property; to have ever claimed eligibility for free school meals; and have lower parental social class ($p<0.001$). The sample used is clearly not a random sample of those who enrolled, however for bias to be present in our multivariable models would require the dependent variable (educational attainment) to be conditionally related to whether participants are included or excluded from this analysis. The pattern of positive associations observed between various factors and selection might lead us to anticipate collider bias in the form of attenuated estimates for MRB and educational attainment. However, by conditioning on gender, IQ, income, free school meal eligibility, parental social class as well as multiple risk behaviour in the regression models, we propose that any residual association between educational attainment and selection should be minimal and hence so should any (attenuating) bias. Fourth, some of the MRB were assessed using questionnaires, which have potential for recall bias and social desirability bias. Finally, we may not have controlled for all relevant confounders, for example, lone parent status and child maltreatment which are both associated with poorer educational outcomes were not included in the analysis. We have included multiple alternative confounders (parental social class, maternal education, housing tenure and claiming eligibility for free school meals), that are all themselves strongly associated with lone parent status and childhood maltreatment, however their omission may still be understood as a significant limitation of the study.

The findings from this research build on and are consistent with other studies which have shown positive associations between single risk behaviours or small numbers of similar risks

For peer review only - http://bmjopen.bmj.com/site/about/guidelines.xhtml

and poorer educational attainment. [3,4,16-22,40-42] They also echo findings from a US study which found strong evidence of associations between higher educational attainment and membership to the most-healthy cluster of adolescents (although that study did not show the same dose-response relationship that we have found). [15]

Establishing the direction of the association between MRB and educational attainment would provide a valuable focus for future work in this area. Analysis of repeated measures of MRB at different time points throughout childhood and adolescence, would allow the exploration of any differences in the association between MRB and educational outcomes according to timing of MRB. Further research is also required to identify the early-life antecedents that are associated with adolescent MRB, as this would facilitate effective early intervention of those with the highest risk of engaging in harmful MRB.

Conclusions

Our findings demonstrate for the first time that multiple health risk behaviours act as an important predictor of adverse educational outcomes, over and above a wide range of confounders including IQ at age 8 years and previous educational attainment. This finding could aid the identification and targeting of young people at risk of under-achieving during their compulsory education. Further, by showing a dose-response relationship between the two, we have shown the importance of intervening in and reducing each and every risk behaviour. Preventing multiple risk behaviours during adolescence could improve educational attainment and thereby directly and indirectly improve longer term health outcomes.

Contributorship Statement

CW, RK, MH, RC and JH conceived and designed the study. CW and JH carried out the study, including acquiring and analysing the data. CW and JH interpreted the data. CW drafted the manuscript. RK, MH, RC and JH critiqued the manuscript for important

For peer review only - http://bmjopen.bmj.com/site/about/guidelines.xhtml

intellectual content. All authors have read and approved the final version of the manuscript. CW serves as guarantor. MH and RC are senior investigators for the National Institute for Health Research. CW and JH had full access to all of the data and can take responsibility for the integrity of the data and the accuracy of the data analysis. CW, RK, MH, RC and JH had access to the statistical outputs, reports, and tables. All researchers are independent of the funding bodies.

Data sharing statement

The ALSPAC policy on data sharing is available at www.bristol.ac.uk/alspac. To discuss access to ALSPAC data, please contact the ALSPAC executive team on alspac-exec@bristol.ac.uk.

Acknowledgements

We are extremely grateful to all the families who took part in this study, the midwives for their help in recruiting them, and the whole ALSPAC team, which includes interviewers, computer and laboratory technicians, clerical workers, research scientists, volunteers, managers, receptionists and nurses. The UK Medical Research Council and the Wellcome Trust (Grant ref: 102215/2/13/2) and the University of Bristol provide core support for ALSPAC.

For peer review only

For peer review only - http://bmjopen.bmj.com/site/about/guidelines.xhtml

REFERENCES

1. de Winter AF, Leenke V, Verhulst FC, Vollebergh WAM, Reijneveld SA. Longitudinal patterns and predictors of multiple health risk behaviors among adolescents: The TRAILS study. *Prev Med* 2015; **84**: 76-82.
2. Mays D, Peshkin BN, Sharff ME, et al. Correlates of adherence to a telephone-based multiple health behavior change cancer preventive intervention for teens: the Healthy for Life Program (HELP). *Health Education and Behavior* 2012; **39**(1): 18-26.
3. Booth JN, Tomporowski PD, Boyle JME, et al. Obesity impairs academic attainment in adolescence: findings from ALSPAC, a UK cohort. *Int J Obes (Lond)* 2014; **38**(10): 1335-42.
4. Latvala A, Rose RJ, Pulkkinen L, Dick DM, Korhonen T, Kaprio J. Drinking, smoking, and educational achievement: cross-lagged associations from adolescence to adulthood. *Drug and Alcohol Dependence* 2014; **137**: 106-13.
5. McCullough ML, Patel AV, Kushi LH, et al. Following cancer prevention guidelines reduces risk of cancer, cardiovascular disease, and all-cause mortality. *Cancer Epidemiology, Biomarkers & Prevention* 2011; **20**(6): 1089-97.
6. Kvaavik E, Batty D, Ursin G, Huxley R, Gale C. Influence of Individual and Combined Health Behaviors on Total and Cause-Specific Mortality in Men and Women. *Archives of Internal Medicine* 2010; **170**(8): 711-8.
7. Connor J. Alcohol consumption as a cause of cancer. *Addiction* 2016; **112**(2): 222–8.
8. Tamakoshi A, Tamakoshi K, Lin Y, Yagyu K, Kikuchi S, Group JS. Healthy lifestyle and preventable death: findings from the Japan Collaborative Cohort (JACC) Study. *Prev Med* 2009; **48**(5): 486-92.
9. Spring B, Schneider K, McFadden HG, et al. Make Better Choices (MBC): Study design of a randomized controlled trial testing optimal technology-supported change in multiple diet and physical activity risk behaviors. *BMC Public Health* 2010; **10**(1): 586.
10. Gilman SE, Martin LT, Abrams DB, et al. Educational attainment and cigarette smoking: a causal association? *Int J Epidemiol* 2008; **37**(3): 615-24.
11. Marmot M. Fair society healthy lives full report. 2010.
12. Ward JL, Viner RM. Secondary Education and Health Outcomes in Young People from the Cape Area Panel Study (CAPS). *PLoS One* 2016; **11**(6): 1-14.
13. ONS. Full Report - Graduates in the UK Labour. 2013.
14. Education Df. Youth Cohort Study & Longitudinal study of young people in England: The Activities and Experiences of 19 year olds: England 2010. In: Education Df, editor.; 2011.
15. Skalamera J, Hummer RA. Educational attainment and the clustering of health-related behavior among U.S. young adults. *Prev Med* 2016; **84**: 83-9.
16. Caird J, Kavanagh J, Oliver K, et al. Childhood obesity and educational attainment a systematic review. London: EPPI-Centre, Social Science Research Unit, Institute of Education, University of London: Institute of Education, University of London, 2011.
17. Stiby AI, Hickman M, Munafo MR, Heron J, Yip VL, Macleod J. Adolescent cannabis and tobacco use and educational outcomes at age 16: birth cohort study. *Addiction* 2015; **110**(4): 658-68.
18. Silins E, Fergusson DM, Patton GC, et al. Adolescent substance use and educational attainment: An integrative data analysis comparing cannabis and alcohol from three Australasian cohorts. *Drug and Alcohol Dependence* 2015; **156**: 90-6.

For peer review only - http://bmjopen.bmj.com/site/about/guidelines.xhtml

19. Verweij KJ, Huizink AC, Agrawal A, Martin NG, Lynskey MT. Is the relationship between early-onset cannabis use and educational attainment causal or due to common liability? *Drug and Alcohol Dependence* 2013; **133**(2): 580-6.
20. Mars B, Heron J, Crane C, et al. Clinical and social outcomes of adolescent self harm: population based birth cohort study. *BMJ* 2014; **349**: 1-13.
21. Booth JN, Leary SD, Joinson C, et al. Associations between objectively measured physical activity and academic attainment in adolescents from a UK cohort. *British Journal of Sports Medicine* 2014; **48**(3): 265-70.
22. Corder K, Atkin AJ, Bamber DJ, et al. Revising on the run or studying on the sofa: prospective associations between physical activity, sedentary behaviour, and exam results in British adolescents. *International Journal of Behavioral Nutrition and Physical Activity* 2015; **12**: 106.
23. Kipping RR, Smith M, Heron J, Hickman M, Campbell R. Multiple risk behaviour in adolescence and socio-economic status: findings from a UK birth cohort. *European Journal of Public Health* 2015; **25**(1): 44-9.
24. Alzahrani SG, Watt RG, Sheiham A, Aresu M, Tsakos G. Patterns of clustering of six health-compromising behaviours in Saudi adolescents. *BMC Public Health* 2014; **14**: 1215.
25. de Looze M, Ter Bogt TFM, Raaijmakers QAW, Pickett W, Kuntsche E, Vollebergh WAM. Cross-national evidence for the clustering and psychosocial correlates of adolescent risk behaviours in 27 countries. *European Journal of Public Health* 2015; **25**(1): 50-6.
26. Busch V, Van Stel HF, Schrijvers AJP, de Leeuw JRJ. Clustering behaviours outcomes Dutch adolescents. *BMC Public Health* 2013; **13**: 1-11.
27. Noel H, Denny S, Farrant B, et al. Clustering of adolescent health concerns: a latent class analysis of school students in New Zealand. *Journal of Paediatrics and Child Health* 2013; **49**(11): 935-41.
28. van Nieuwenhuijzen M, Junger M, Velderman MK, et al. Clustering of health-compromising behavior and delinquency in adolescents and adults in the Dutch population. *Prev Med* 2009; **48**(6): 572-8.
29. Buck D, F. F. Clustering of unhealthy behaviours over time: Implications for policy and practice. . London: Kings Fund, 2012.
30. Lawder R, Harding O, Stockton D, et al. Is the Scottish population living dangerously? Prevalence of multiple risk factors: the Scottish Health Survey 2003. *BMC Public Health* 2010; **10**(1): 330.
31. Shankar A, McMunn A, Steptoe A. Health-Related Behaviors in Older Adults: Relationships with Socioeconomic Status. *American Journal of Preventive Medicine* 2010; **38**(1): 39-46.
32. Boyd A, Golding J, Macleod J, et al. Cohort Profile: the 'children of the 90s'-the index offspring of the Avon Longitudinal Study of Parents and Children. *Int J Epidemiol* 2013; **42**(1): 111-27.
33. ALSPAC. Avon Longitudinal Study of Parents and Children. ALSPAC: Data Dictionary. 2016.
34. Hurrelmann K, Richter M. Risk behaviour in adolescence: the relationship between developmental and health problems. *J Public Health* 2006; **14**(1): 20-8.
35. MacArthur GJ, Smith MC, Melotti R, et al. Patterns of alcohol use and multiple risk behaviour by gender during early and late adolescence: the ALSPAC cohort. *J Public Health* 2012; **34**(suppl_1): i20-i30.
36. Heron J, Campbell R, Hickman M, Kipping RR. Latent class analysis of multiple risk behaviour in adolescents in the ALSPAC cohort. *BMC Public Health* under review.

For peer review only - http://bmjopen.bmj.com/site/about/guidelines.xhtml

37. Crawford C, Dearden L, Greaves E. The drivers of month-of-birth differences in children's cognitive and non-cognitive skills. *Journal of the Royal Statistical Society Series A, (Statistics in Society)* 2014; **177**(4): 829-60.
38. Sterne J, White J, Carlin J, et al. Multiple imputation for missing data in epidemiological and clinical research: potential and pitfalls. *BMJ* 2009; **338**: b2393.
39. White IR, Royston P, Wood AM. Multiple imputation using chained equations: Issues and guidance for practice. *Stat Med* 2011; **30**(4): 377-99.
40. Grant JD, Scherrer JF, Lynskey MT, et al. Associations of alcohol, nicotine, cannabis, and drug use/dependence with educational attainment: evidence from cotwin-control analyses. *Alcohol Clin Exp Res* 2012; **36**(8): 1412-20.
41. Horwood LJ, Fergusson DM, Hayatbakhsh MR, et al. Cannabis use and educational achievement: findings from three Australasian cohort studies. *Drug Alcohol Depend* 2010; **110**(3): 247-53.
42. Staff J, Patrick ME, Loken E, Maggs J. Teenage Alcohol Use and Educational Attainment. *Journal of Studies on Alcohol and Drugs* 2008; **69**(6): 848-58.

For peer review only

For peer review only - http://bmjopen.bmj.com/site/about/guidelines.xhtml

Table 1: Multiple risk behaviours (MRB) and their derivation

Health risk behaviour	Definition / how derived
Physical inactivity	Young person (YP) has typically over the past year exercised <5 times per week.
TV viewing	YP spent 3 or more hours watching television on average per day across the week.
Car passenger risk	YP had been in a car passenger at least once in their lifetime where the driver (a) had consumed alcohol or (b) did not have a valid licence, or (c) the YP chose not to wear a seat belt last time travelled in a car, van, or taxi.
Cycle helmet use	If the YP reported that they had last ridden a bicycle within the previous four weeks and they had not worn a helmet on the most recent occasion.
Scooter risk	YP has driven a motorbike/ scooter off road, or without a licence on a public road at least once.
Criminal/Antisocial behaviour	YP reported that at least once in the past year they had undertaken at least one of the following 7 offences: carried a weapon; physically hurt someone on purpose; stolen something; sold illicit substances to another person; damaged property belonging to someone else either by using graffiti, setting fire to it or destroying or damaging it in another fashion; subjected someone to verbal or physical racial abuse; or been rude/rowdy in a public place.
Hazardous alcohol consumption	In the past year had scored eight or more on the Alcohol Use Disorders Identification Test (AUDIT) indicating hazardous alcohol consumption.
Regular tobacco smoking	Has ever smoked and is regularly smoking by currently smoking at least one cigarette per week.
Cannabis use	Those who reported using cannabis “sometimes but less often than once

For peer review only - http://bmjopen.bmj.com/site/about/guidelines.xhtml

	a week" or more regular use were classified as occasional users.
Illicit drug/solvent use	In the year since their 15th birthday, YP had either been a regular user (i.e. used five or more times) of one or more illicit drugs (excluding cannabis) including amphetamines, ecstasy, LSD, cocaine, ketamine or inhalants including aerosols, gas, solvents and poppers.
Self-harm	Young people who said they had purposely hurt themselves in some way in their lifetime.
Penetrative sex before age 16	YP reported having had penetrative sex in the preceding year and that they were under 16 at the time.
Unprotected sex	Penetrative sex without the use of contraception on the last occasion they had had sex in the past year

Sources of information:

Age 15 years clinic: criminal and antisocial behaviour, penetrative sex prior to age 16, and unprotected sex.

Age 16 years questionnaire: physical inactivity, TV viewing, car passenger risk, cycle helmet use, scooter risk, hazardous alcohol drinking, regular smoking, illicit drug/solvent use, and self-harm.

For peer review only - http://bmjopen.bmj.com/site/about/guidelines.xhtml

Table 2: Sample descriptive statistics

		Imputation sample	Complete case sample
		**5,401**	**1,617**
		**% (SE)**	**n (%)**
Educational Outcomes	n=5,401		
Capped GCSE score: Mean (SE)		350 (1·01)	376 (1·31)
Five or more A*-C grades at GCSE		74·4% (SE=0·6)	1420 (87·8%)
Four or less A*-C grades at GCSE		25·6% (SE=0·6)	197 (12·2%)
Exposure	n=2,264		
MRB total: Mean (SE)		3.24 (0·31)	3.01 (0·05)
Sex	n=5,401		
Female		55·5% (SE=0·7)	975 (60·3%)
Male		44·5% (SE=0·7)	642 (39·7%
Season of birth	n=5,401		
Autumn		33·9% (SE=0·6)	542 (33·5%)
Winter		14·8% (SE=0·5)	222 (13·7%)
Spring		23·4% (SE=0·6)	382 (23·6%)
Summer		27·9% (SE=0·6)	471 (29·1%)
Maternal education	n=5,214		
Degree		14·9% (SE=0·5)	311 (19·2%)
A level		25·9% (SE=0·6)	479 (29·6%)
O level		34·3% (SE=0·7)	596 (36·9%)
< O level		21·5% (SE=0·6)	231 (14·3%)
Parental socio-economic position	n=4,970		
Professional		14·1% (SE=0·5)	291 (18·0%)
Managerial and technical		41·2% (SE=0·7)	761 (47·1%)

For peer review only - http://bmjopen.bmj.com/site/about/guidelines.xhtml
For peer review only

Skilled non-man		23·6% (SE=0·6)	398 (24·6%)
Skilled man, part or unskilled		13·2% (SE=0·5)	167 (10·3%)
Housing tenure	n=5,227		
Mortgage/owned		84·7% (SE=0·5)	1.444 (89·3%)
Private rent		7·3% (SE=0·4)	88 (5·4%)
Subsidised rent		8·6% (SE=0·4)	85 (5·3%)
Income	n=4,809		
High		20.86% (SE=0·6)	364 (22·5%)
Mid high		21.96% (SE=0·6)	424 (26·2%)
Middle		21.34% (SE=0·6)	364 (22·5%)
Mid low		19.25% (SE=0·6)	292 (18·1%)
Low		16.58% (SE=0·5)	173 (10·7%)
Free School Meals (FSM)	n=5,401		
Ever FSM		7·7% (SE=0·4)	65 (4·0%)
Never FSM		92·3% (SE=0·4)	1,552 (96·0%)
Special Educational Needs (SEN)	n=5,075		
No SEN		84·7% (SE=0·4)	1,522 (94·1%)
School Action		6·0% (SE=0·4)	68 (4·2%)
School Action Plus		1·9% (SE=0·2)	18 (1·1%)
Statement of SEN		1·4% (SE=0·2)	9 (0·6%)
Previous educational attainment/ability			
IQ at age 8: Mean (SE)	n=4,370	105 (0·24)	109 (0·37)
KS2 educational attainment: Mean (SE)	n=4,753	830 (2·76)	901 (3·94)

For peer review only - http://bmjopen.bmj.com/site/about/guidelines.xhtml

Table 3: Associations between total MRB score and capped GCSE score and odds of gaining five or more GCSEs at grade A*-C

	Unadjusted (n=5,401)	Model 1 (n=5,401)	Model 2 (n=5,401)	Model 3 (n=5,401)
**Capped GCSE**	-9·17 (-10·25, -8·10) p<0·001	-9·12 (-10·19, -8·05) p<0·001	-6·90 (-7·86, -5·94) p<0·001	-6·31 (-7·03, -5·58) p<0·001
**5 A*-C**	0·82 (0·79-0·85) p<0·001	0·82 (0·79-0·85) p<0·001	0·84 (0·81-0·87) p<0·001	0·77 (0·74-0·81) p<0·001

Model 1: adjusted for sex and season of birth

Model 2: adjusted for sex, season of birth, maternal education, parental social class, household income, housing tenure, and FSM.

Model 3: adjusted for sex, season of birth, maternal education, parental social class, household income, housing tenure, FSM, IQ, and previous educational attainment.

For peer review only - http://bmjopen.bmj.com/site/about/guidelines.xhtml

Figure 1: Deriving the sample

For peer review only

For peer review only - http://bmjopen.bmj.com/site/about/guidelines.xhtml

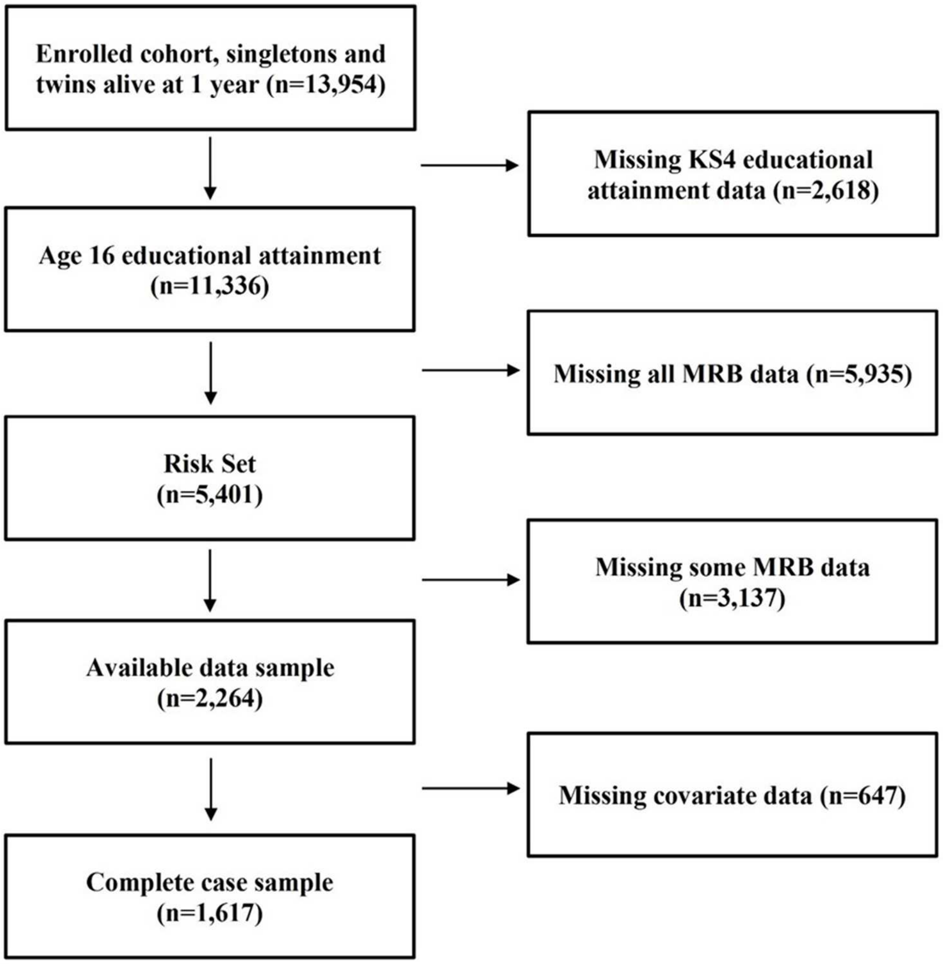

Figure 1: Deriving the sample

82x87mm (300 x 300 DPI)

Supplementary material table 1: Assessment for confounding, univariable associations with the exposure and outcome variables

	MRBs	Capped GCSE	Five or more GCSEs A*-C
**Gender ref: male**			
Female	-0.06 (-0.18, 0.06) p=0.343	10.83 (6.85, 14.81) p<0.001	1.32 (1.17, 1.49) p<0.001
**Season of birth ref: autumn**			
Winter	-0.04 (-0.23, 0.15) p=0.700	-4.18 (-10.35, 1.99) p=0.184	0.95 (0.79, 1.16) p=0.637
Spring	0.04 (-0.13, 0.20) p=0.674	-10.37 (-15.69, -5.05) p<0.001	0.78 (0.67, 0.92) p=0.004
Summer	0.26 (-0.13, 0.18) p=0.744	-13.34 (-18.40, -8.28) p<0.001	0.83 (0.71, 0.97) p=0.018
**Maternal education ref: degree**			
A level	0.17 (-0.02, 0.36) p=0.074	-35.19 (-41.13, -29.25) p<0.001	0.18 (0.12, 0.27) p<0.001
O level	0.41 (0.23, 0.59) p<0.001	-57.89 (-63.55, -52.23) p<0.001	0.09 (0.06, 0.14) p<0.001
< O level	0.49 (0.28, 0.69) p<0.001	-90.81 (-96.95, -84.68) p<0.001	0.04 (0.25, 0.06) p<0.001
**Parental socio-economic position ref: professional**			
Managerial and technical	0.43 (0.25, 0.61) p<0.001	-35.76 (-41.46, -30.05) p<0.001	0.29 (0.21, 0.39) p<0.001
Skilled non-man	0.47 (0.28, 0.67) p<0.001	-58.39 (-64.63, -52.15) p<0.001	0.15 (0.11, 0.21) p<0.001
Skilled man, part or unskilled	0.83 (0.60, 1.05) p<0.001	-88.71 (-95.76, -81.64) p<0.001	0.07 (0.05, 0.09) p<0.001

For peer review only

**Housing tenure ref: mortgage/owned**			
Private rent	0 .38 (0.14, 0.61) p=0.002	-20.07 (-27.50, -12.64) p<0.001	0.59 (0.47, 0.74) p<0.001
Subsidised rent	0 .75 (0.53, 0.97) p<0.001	-71.86 (-78.71, -65.01) p<0.001	0.18 (0.15, 0.74) p<0.001
**Income ref: high**			
Mid high	0.05 (-0.14, 0.24) p=0.615	-12.17 (-18.09, -6.26) p<0.001	0.62 (0.47, 0.80) p<0.001
Middle	0.17 (-0.02, 0.36) p=0.084	-32.02 (-37.98, -26.07) p<0.001	0.34 (0.26, 0.43) p<0.001
Mid low	0.35 (0.15, 0.55) p<0.001	-45.62 (-51.78, -39.46) p<0.001	0.23 (0.18, 0.29) p<0.001
Low	0.43 (0.23, 0.64) p<0.001	-74.31 (-80.75, -67.87) p<0.001	0.13 (0.10, 0.16) p<0.001
**FSM ref: never FSM**			
Ever FSM	0.97 (0.74, 1.21) p<0.001	-68.52 (-75.74, -61.30) p<0.001	0.22 (0.18, 0.27) p<0.001
**IQ at age 8**	-.0009 (-.0010, -.0006) p=0.009	2.69 (2.58, 2.79) p<0.001	1.09 (1.09, 1.10) p<0.001
**KS2 educational attainment**	-0.01 (-0.01, -0.01) p<0.001	0.26 (0.259, 0 .278) p<0.001	1.01 (1.01, 1.01) p<0.001
**SEN ref: no SEN**			
School Action	0.76 (-0.18, 0.33) p=0.555	-64.63 (-73.46, -55.80) p<0.001	0.19 (0.15, 0.25) p<0.001
School Action Plus	0.30 (-0.16, 0.77) p=0.201	-82.35 (-97.93, -66.78) p<0.001	0.13 (0.09, 0.21) p<0.001
Statement of SEN	-0.39 (-0.90, 0.12) p=0.134	-133.76 (-152.96, -114.56) p<0.001	0.11 (0.06, 0.19) p<0.001

For peer review only

Supplementary material table 2: Associations between total MRB score and educational outcomes, capped GCSE score and gaining five or more GCSEs at grade A*-C (complete case analysis)

	Complete Case Unadjusted (n=1,617)	Complete Case Adjusted[1] (n=1,617)	Complete Case Adjusted[2] (n=1,617)	Complete Case Adjusted[3] (n=1,617)
**Capped GCSE**	-6.12 (-7.42, -4.83)<br>p<0.001	-6.13 (-7.42, -4.84)<br>p<0.001	-4.82 (-6.00, -3.64)<br>p<0.001	-4.48 (-5.31, -3.64)<br>p<0.001
**5 A*-C**	0.81 (0.76–0.87)<br>p<0.001	0.81 (0.75–0.87)<br>p<0.001	0.84 (0.77-0.90)<br>p<0.001	0.75 (0.68–0.83)<br>p<0.001

[1] Adjusted for sex and season of birth.
[2] Adjusted for sex, season of birth maternal education, parental social class, household income, tenure and FSM.
[3] Adjusted for sex, season of birth maternal education, parental social class, household income, tenure FSM, IQ at age 8 and previous educational attainment at key stage 2.

For peer review only

Supplementary material table 3: Associations between separate MRB and educational outcomes, capped GCSE score and gaining five or more GCSEs at grade A*-C

	Capped GCSE score	Five or more GCSEs A*-C
**Car passenger risk**	-19.90 (-25.13, -14.67) P<0.001	0.68 (0.58, 0.80) P<0.001
**Moped risk**	-35.18 (-41.51, -28.85) P<0.001	0.42 (0.35, 0.51) P<0.001
**Cycle helmet risk**	-5.41 (-10.93, 0.12) P=0.055	0.79 (0.68, 0.94) P=0.007
**Drug/solvent use**	-23.65 (-36.29, -10.99) P<0.001	0.81 (0.57, 1.14) p=0.225
**Cannabis use**	-20.59 (-28.76, -12.42) P<0.001	0.71 (0.56, 0.89) P=0.003
**Tobacco smoking**	-57.40 (-63.86, -50.94) P<0.001	0.30 (0.25, 0.36) P<0.001
**Hazardous alcohol use**	-5.56 (-10.69, -0.43) P=0.034	0.91 (0.77, 1.06) P=0.228
**Combined sexual risk**	-28.57 (-34.42, -22.72) P<0.001	0.55 (0.47, 0.65) P<0.001
**Self-harm**	-12.24 (-18.17, -6.31) P<0.001	0.83 (0.70, 0.99) P=0.041

For peer review only

**Criminal/Antisocial behaviour**	-22.76 (-27.2, -18.32)	0.58 (0.50, 0.66)
	P<0.001	P<0.001
**Physical inactivity**	-0.24 (-5.80, 5.33)	1.10 (0.92, 1.31)
	P=0.934	P=0.284
**TV viewing**	-23.97 (-29.82, -18.11)	0.60 (0.50, 0.72)
	P<0.001	P<0.001

For peer review only

STROBE Statement—Checklist of items that should be included in reports of ***cohort studies***

	Item No	Recommendation
**Title and abstract**	1	(*a*) Indicate the study's design with a commonly used term in the title or the abstract
**Pages 1 &2**		(*b*) Provide in the abstract an informative and balanced summary of what was done and what was found
**Introduction**		
Background/rationale **Page 4**	2	Explain the scientific background and rationale for the investigation being reported
Objectives **Page 4**	3	State specific objectives, including any prespecified hypotheses
**Methods**		
Study design **Page 5**	4	Present key elements of study design early in the paper
Setting **Page 5**	5	Describe the setting, locations, and relevant dates, including periods of recruitment, exposure, follow-up, and data collection
Participants	6	(*a*) Give the eligibility criteria, and the sources and methods of selection of participants. Describe methods of follow-up
**Page 4**		(*b*) For matched studies, give matching criteria and number of exposed and unexposed
Variables **Page 6&7**	7	Clearly define all outcomes, exposures, predictors, potential confounders, and effect modifiers. Give diagnostic criteria, if applicable
Data sources/ measurement **Pages 5-7**	8*	For each variable of interest, give sources of data and details of methods of assessment (measurement). Describe comparability of assessment methods if there is more than one group
Bias **Pages 7&8**	9	Describe any efforts to address potential sources of bias
Study size **Page 7&8**	10	Explain how the study size was arrived at
Quantitative variables **Pages 6-8**	11	Explain how quantitative variables were handled in the analyses. If applicable, describe which groupings were chosen and why
Statistical methods	12	(*a*) Describe all statistical methods, including those used to control for confounding
		(*b*) Describe any methods used to examine subgroups and interactions
**Pages 5-9**		(*c*) Explain how missing data were addressed
		(*d*) If applicable, explain how loss to follow-up was addressed
		(*e*) Describe any sensitivity analyses
**Results**		
Participants	13*	(a) Report numbers of individuals at each stage of study—eg numbers potentially eligible, examined for eligibility, confirmed eligible, included in the study, completing follow-up, and analysed
**Pages 7&8**		(b) Give reasons for non-participation at each stage
		(c) Consider use of a flow diagram
Descriptive data	14*	(a) Give characteristics of study participants (eg demographic, clinical, social) and information on exposures and potential confounders
**Page 10**		(b) Indicate number of participants with missing data for each variable of interest
		(c) Summarise follow-up time (eg, average and total amount)
Outcome data **Pages 5, 7**	15*	Report numbers of outcome events or summary measures over time

For peer review only - http://bmjopen.bmj.com/site/about/guidelines.xhtml

Main results<br><br>**Pages 12-14**	16	(*a*) Give unadjusted estimates and, if applicable, confounder-adjusted estimates and their precision (eg, 95% confidence interval). Make clear which confounders were adjusted for and why they were included
		(*b*) Report category boundaries when continuous variables were categorized
		(*c*) If relevant, consider translating estimates of relative risk into absolute risk for a meaningful time period
Other analyses<br>**Pages 9**	17	Report other analyses done—eg analyses of subgroups and interactions, and sensitivity analyses
**Discussion**		
Key results<br>**Page 10&11**	18	Summarise key results with reference to study objectives
Limitations<br>**Pages 3 & 12**	19	Discuss limitations of the study, taking into account sources of potential bias or imprecision. Discuss both direction and magnitude of any potential bias
Interpretation<br>**Pages 11&12**	20	Give a cautious overall interpretation of results considering objectives, limitations, multiplicity of analyses, results from similar studies, and other relevant evidence
Generalisability<br>**Page 8**	21	Discuss the generalisability (external validity) of the study results
**Other information**		
Funding<br>**Page 3**	22	Give the source of funding and the role of the funders for the present study and, if applicable, for the original study on which the present article is based

*Give information separately for exposed and unexposed groups.

Note: An Explanation and Elaboration article discusses each checklist item and gives methodological background and published examples of transparent reporting. The STROBE checklist is best used in conjunction with this article (freely available on the Web sites of PLoS Medicine at http://www.plosmedicine.org/, Annals of Internal Medicine at http://www.annals.org/, and Epidemiology at http://www.epidem.com/). Information on the STROBE Initiative is available at http://www.strobe-statement.org.

For peer review only
